# Social vulnerability indices: a scoping review

**DOI:** 10.1186/s12889-023-16097-6

**Published:** 2023-06-28

**Authors:** Jasmine Cassy Mah, Jodie Lynn Penwarden, Henrique Pott, Olga Theou, Melissa Kathryn Andrew

**Affiliations:** 1 Department of Medicine, Dalhousie University, Halifax, NS Canada; 2 Geriatric Medicine, Dalhousie University and Nova Scotia Health, Halifax, NS Canada; 3 Department of Medicine, Federal University of São Carlos (UFSCar), São Carlos, SP Brazil; 4 School of Physiotherapy, Dalhousie University, Halifax, NS Canada; 5 Division of Geriatric Medicine, Dalhousie University, Halifax, NS Canada

**Keywords:** Social vulnerability, Social vulnerability index, Indices, Disaster planning, Environment, Climate, Tools

## Abstract

**Background:**

Social vulnerability occurs when the disadvantage conveyed by poor social conditions determines the degree to which one’s life and livelihood are at risk from a particular and identifiable event in health, nature, or society. A common way to estimate social vulnerability is through an index aggregating social factors. This scoping review broadly aimed to map the literature on social vulnerability indices. Our main objectives were to characterize social vulnerability indices, understand the composition of social vulnerability indices, and describe how these indices are utilized in the literature.

**Methods:**

A scoping review was conducted in six electronic databases to identify original research, published in English, French, Dutch, Spanish or Portuguese, and which addressed the development or use of a social vulnerability index (SVI). Titles, abstracts, and full texts were screened and assessed for eligibility. Data were extracted on the indices and simple descriptive statistics and counts were used to produce a narrative summary.

**Results:**

In total, 292 studies were included, of which 126 studies came from environmental, climate change or disaster planning fields of study and 156 studies were from the fields of health or medicine. The mean number of items per index was 19 (SD 10.5) and the most common source of data was from censuses. There were 122 distinct items in the composition of these indices, categorized into 29 domains. The top three domains included in the SVIs were: at risk populations (e.g., % older adults, children or dependents), education, and socioeconomic status. SVIs were used to predict outcomes in 47.9% of studies, and rate of Covid-19 infection or mortality was the most common outcome measured.

**Conclusions:**

We provide an overview of SVIs in the literature up to December 2021, providing a novel summary of commonly used variables for social vulnerability indices. We also demonstrate that SVIs are commonly used in several fields of research, especially since 2010. Whether in the field of disaster planning, environmental science or health sciences, the SVIs are composed of similar items and domains. SVIs can be used to predict diverse outcomes, with implications for future use as tools in interdisciplinary collaborations.

**Supplementary Information:**

The online version contains supplementary material available at 10.1186/s12889-023-16097-6.

## Background

There has been increased interest in understanding social vulnerability within medical sciences and medical practice. Social vulnerability in medicine is bi-directional; it contributes to the factors which increase risk of adverse health conditions and has practical implications for arranging supports after an adverse health event. Social vulnerability provides a way to understand how the broader conditions in which people are born, live, work and age can worsen an unfortunate event (e.g., a health crisis) into a veritable disaster [1, 2]. Reducing social vulnerability through modification of social conditions opens intervention opportunities to prevent or reduce suffering after a health event.

A better understanding of social vulnerability can be elucidated by examining interdisciplinary social vulnerability research. Social vulnerability has roots in a rich and evolving literature base involving various natural, health and social disciplines. For example, a review of social vulnerability in climate change helps make sense of the complexity of this concept [3]. Assessing social vulnerability enables the separation of the biophysical dimension from the human and social dimension of susceptibility to climate events [3]. Moreover, social vulnerability as a concept reflects both the capacity of a system to respond from an impact as well as an intrinsic lack of capability of individuals to cope with external stressors [3]. Another common working definition in disaster planning refers to the “characteristics of a person or group in terms of their capacity to anticipate, cope with, resist and recover from the impact of a natural hazard” [4], often compounded by the inability of the external system to respond. Similarly, adverse events, whether disaster or health-related, tend to expose, and make it possible to capture, the pre-existing social inadequacies that make individuals or communities disproportionately vulnerable. When we apply this to social vulnerability within medicine, it can be viewed as describing the non-health dimensions that keeps individuals incapacitated longer than expected (e.g., in hospital unable to return home) because of social circumstances close to the individual (e.g., marital status) but also because of social support systems that fail to respond or perpetuate vulnerability (e.g., lack of affordable housing for people with disabilities).

Regardless of field, social vulnerability research strives to understand the social environment not just as a descriptor but as a predictor of vulnerability relative to changes in the environment, social circumstances, disasters, or health status [1, 5–7]. To this end, estimating and quantifying social vulnerability is necessary. A common way to estimate social vulnerability is through an index aggregating social indicators. This approach has several benefits, including the opportunity to include variables from different categories of social factors (e.g., socioeconomic status, social engagement, social capital) instead of a one-at-a-time approach [1]. An index does not arbitrarily separate related factors into distinct categories for separate analysis. Moreover, it allows for gradations in social vulnerability (instead of binary or ordinal social variables) and for scaling to account for different units of analysis [1, 8]. In practical terms, an index overcomes the following dilemma. Two households with an average income below the poverty line may be classified as vulnerable in a study examining household income. Suppose one household comprises a recently graduated, working-age, married couple well integrated into the community with strong social ties, and the other is an older adult living off government assistance with no friends or family who could help in a time of need. Thus, there are two distinct tiers of social vulnerability not captured by examining household income alone.

One problem with an index approach is deciding which social factors to include in a social vulnerability index. Items to be included can be limitless or highly context dependent. Cutter and colleagues [9] have previously noted that there are no accepted sets of variables for vulnerability to climate change, but there is general consensus on using age, gender, race and socio-economic status; while necessary, these four factors are insufficient to give the full picture of social vulnerability. Furthermore, a worthwhile endeavour may be to create a social vulnerability index relevant to medical and health contexts, yet there have been only a few social vulnerability indices published specific to medicine [10–12]. Looking at the social factors composing these few indices relevant to medicine does not provide breath of social conditions if we understand social vulnerability to be an interdisciplinary concept encompassing both the individual’s and system’s inability to cope. Expanding this pool of commonly used variables to include in a social vulnerability index would provide additional benefit for future indices, and for indices relevant to medicine.

This scoping review broadly maps the literature on social vulnerability indices. Our three main objectives were: (1) to characterize social vulnerability indices, (2) to understand the composition of social vulnerability indices, and (3) to describe how these indices are utilized in the literature.

## Methods

This scoping review uses the Arksey and O’Malley framework refined by Levac, Colquohoun, and O’Brien [13, 14]. We also followed the PRISMA checklist for scoping reviews (see Additional file 1).

### Information sources

An electronic search was carried out to locate publications in the following databases: Medical Literature Analysis and Retrieval System Online (MEDLINE), Embase, Social Science Citation Index (SSCI), Cumulative Index to Nursing & Allied Health (CINAHL), Public Affairs Index, and Environment Complete from inception to December 1, 2021. No other search terms were included given the specificity of the term “social vulnerability index.mp” or “social vulnerability indic*.mp”. We used the web-based platform Covidence® as the primary screening tool.

### Eligibility criteria

The following inclusion criteria were adopted: (i) original research; (ii) published in English, French, Dutch, Spanish or Portuguese (languages spoken or read by the research team or affiliates); (iii) and which addressed the development or use of a social vulnerability index (hereafter called ‘SVI’). We excluded studies: (i) where a larger index incorporated an SVI and that larger index no longer focused on social vulnerability; (ii) analyzing social factors individually and not the index itself; and (iii) including non-human participants.

### Screening process

Titles and abstracts of search records were screened by two team members independently. We also worked independently to review the full texts of records deemed potentially eligible after the title and abstract screening phase, excluding publications that did not meet the inclusion criteria. Any disagreements were decided by consensus or judication by a third author.

### Data charting process

We extracted data using a piloted data collection form including general study information (reference, year, location), study objective, population, the field of study (we decided a priori to categorize this by: (1) environment, climate or disaster, (2) health or medicine, or (3) other), and composition of the social vulnerability index (items, calculations, scale of measurement, underlying theory). Here, SVI items were the individual questions or statistics (e.g., proportion of institutionalized individuals in a region) comprised in an index. Each SVI constituted one observation in the charting of the index composition and multiple studies using the same constructed SVI were linked. We hand searched reference lists and reported on the earliest publication of the original SVI. Complete information was extracted for studies describing an original SVI (defined by the review authors as the first published study that describes an SVI with least five different items/domains from a previous SVI and a 25% change in a previous SVI’s items). To answer objective 2 regarding the composition of the indices, we established this criterion to avoid overrepresentation of items/domains from frequently replicated SVIs (which may have been reproduced in other datasets with only a few items or domains added or dropped). For studies using a previously described index (hereafter called ‘replications’), we extracted only general information, population unit, field of study, study objectives and outcomes when the SVI was included in predictive modelling. We also emailed authors to get additional information when necessary.

### Synthesis methods

Simple descriptive statistics and counts synthesized the extracted data. We also documented when the SVI was used in an environmental, disaster management, or climate change-related field and when the SVI was used in a health or medicine-related field. To better understand the composition of the SVIs, we tallied each item and re-aggregated the items into domains. The domains were derived from a thematic aggregation of the items in an iterative and consensus-based approach. Finally, we report on a subset of studies that used an SVI to predict outcomes. If the purpose of the SVI was predictive, we recorded and counted the outcomes.

## Results

### Summary of search

**Fig. 1:**
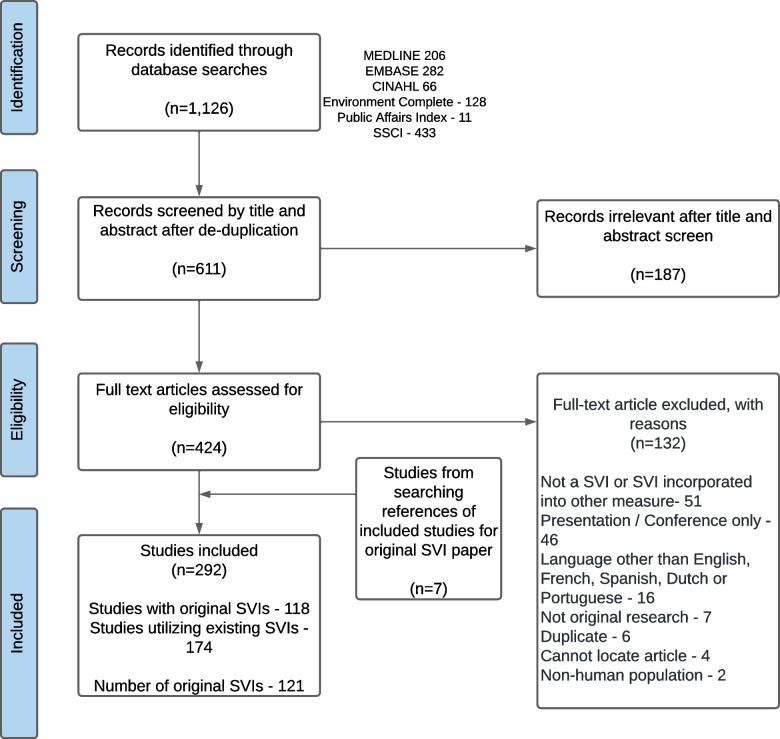
Study selection

The search retrieved 1,126 records of which 515 were duplicates. After screening of titles and abstracts, 187 records were excluded, and 424 full text articles were obtained (see Fig. 1). There are 292 studies included, of which 118 studies examined original SVIs and 174 studies examined replicated SVIs. Of the 118 studies which examined original SVIs, three of the studies examined two SVIs each, therefore the number of original SVIs is 121 (Additional file 2 provides complete references divided into original and replicated SVI studies).

### Study characteristics

The study characteristics are summarized in Table 1 with full details of each SVI available in Additional files 3 and 4. Overall, 53.4% of studies (156/292) reported on the SVI in relation to the fields of health or medicine. Among original SVIs, most were developed for an environmental or disaster planning field (90/118). Of the 292 included studies, 42.8% were conducted in the United States of America (USA) followed by Brazil (18.8%), and 49.7% of studies were conducted after 2019.

**Table 1:** Study characteristics

Articles	All		Environment, Climate or Disaster		Health or Medicine		Other^a^	
	292		126		156		10	
	n	%	n	%	n	%	n	%
Year								
Before 2000	1	0.3	0	0.0	1	0.6	0	0.0
2000–2004	4	1.4	2	1.6	2	1.3	0	0.0
2005–2009	8	2.7	4	3.2	3	1.9	1	10.0
2010–2014	39	13.4	22	17.5	17	10.9	0	0.0
2015–2019	95	32.5	59	46.8	33	21.2	3	30.0
After 2019	145	49.7	39	31.0	100	64.1	6	60.0
SVI								
Original	118	40.4	90	71.4	26	16.7	2	20.0
Replicate	174	59.6	36	28.6	130	83.3	8	80.0
Country								
USA	125	42.8	33	26.2	86	55.1	6	60.0
Brazil	55	18.8	5	4.0	47	30.1	3	30.0
China	22	7.5	22	17.5	0	0.0	0	0.0
Canada	8	2.7	1	0.8	7	4.5	0	0.0
Italy	5	1.7	4	3.2	1	0.6	0	0.0
Romania	5	1.7	5	4.0	0	0.0	0	0.0
India	4	1.4	3	2.4	1	0.6	0	0.0
South Africa	4	1.4	3	2.4	1	0.6	0	0.0
Australia	3	1.0	3	2.4	0	0.0	0	0.0
Indonesia	3	1.0	3	2.4	0	0.0	0	0.0
Multiple in Africa	3	1.0	2	1.6	1	0.6	0	0.0
Netherlands	3	1.0	2	1.6	1	0.6	0	0.0
Spain	3	1.0	3	2.4	0	0.0	0	0.0
Taiwan	3	1.0	2	1.6	1	0.6	0	0.0
Zimbabwe	3	1.0	3	2.4	0	0.0	0	0.0
Other^b^	38	13.0	30	23.8	7	4.2	1	10.0
Multiple countries	5	1.7	2	1.6	3	1.9	0	0.0

^a^Other means mix of fields or another field altogether (e.g., social work, urban design)

^b^Other countries (*n* < 3) included: Algeria, Argentina, Austria, Bangladesh, Barbados, Benin, Botswana, Chile, Colombia, Dominican Republic, France, Egypt, Ghana, Greece, Honduras, Hong Kong, China, Iran, Israel, Japan, Kenya, Lesotho, Liberia, Mexico, Nepal, Nigeria, Pakistan, Palestine, Peru, Philippines, Portugal, Samoa, South Korea, Sri Lanka, Vietnam, Zambia

### Replications

There were 174 studies that used existing SVIs in their research (replications). The most commonly used original SVIs were: the Centers for Disease Control and prevention / Agency for Toxic Substances and Disease Registry’s (CDC/ATSDR) SVI [15], the Social Vulnerability Index (SoVI) by Cutter et al. [9], the SVI by Nahas et al. [16], the Brazilian Social Vulnerability Atlas [17] and the Índice Paulista de Vulnerabilidade Social [18]. The CDC/ATSDR SVI was cited in 51.7% of studies. Most replications (83.3%) were used in papers related to health or medicine. Additional file 4 provides the frequency of replications for each original SVI.

### SVI composition

The mean and median number of items per SVI was 19.3 (SD 10.5) and 18, (IQR 16) ranging from indices with four items [19, 20] to 60 items [21]. SVIs primarily in environment, climate or disaster studies had a mean of 18.8 (SD 10.0) items compared to 21.0 (SD 12.5) items in SVIs in the health or medicine studies. Items were weighted in 43.8% of all SVIs. Almost all SVIs were numeric scales (98.3%).

**Fig. 2:**
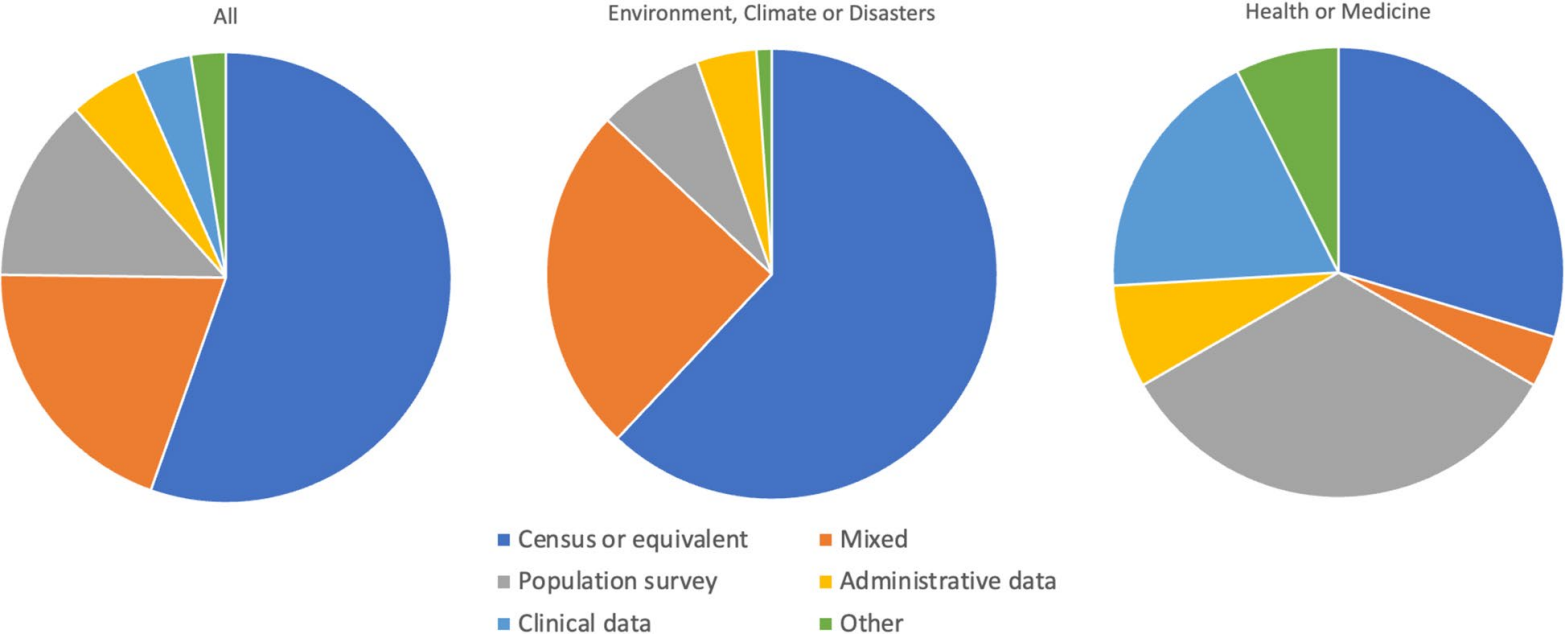
Sources of data for SVI items

As shown in Fig. 2, among all SVIs, 55.4% of the items came from census data, 13.2% had items from population surveys, 5.0% from administrative data, 4.1% from clinical datasets and 2.5% from other sources (e.g., data collected specifically for the SVI). Notably, 19.8% of SVIs were composed of items from at least two of the data types listed previously. Items from SVIs in fields of environment, climate or disaster were collected primarily from census repositories (62.0%) such as the United States Census of Population and Housing, Israeli National Census [20], Barbados’ national decennial census [22], etc., or national geographic data such as Taiwan’s National Geographic Information System [23]. In comparison, studies that included SVIs in health or medicine collected their items 29.6% of the time from census data and 33.3% of the time from population surveys such as the Survey of Health, Ageing and Retirement in Europe [11], Climate Change in the American Mind survey [24], Canadian National Population Health Survey [12], among many others. There were several unique ways to determine the items in an SVI. For example, in one study, items were initially identified through a round of qualitative interviews and a Delphi survey of local professionals and decision makers resulting in a household survey comprised of the items of interest [25]. In another study conducted in Kenya, the items and their coding arose solely from focus groups and qualitative work [26].

### Items and domains

In total, there were 122 distinct items identified. We categorized these items into 29 domains shown in Table 2. The top three domains included in the SVIs were: at risk populations, education and micro level socioeconomic status. Of the 121 original SVIs, 76.0% included an item in the domain of at-risk populations. More (87.0%) environment, climate or disaster SVIs included this domain than health or medicine SVIs (40.7%). Of the 92 SVIs which included an item within the domain of at-risk populations, an item about older adult populations (terms senior or elderly were often used) was most common in 84% of SVIs. In the health or medicine SVIs, the most common item for at risk populations was regarding dependent populations.

**Table 2:** All original SVI domains and items, in descending proportion

Domains and Items in > 50% of SVIs	Domains and Items in 20–50% of SVIs	Domains and Items in < 20% of SVIs
**At risk populations (76.0%)** Older Adults Children Dependents Institutionalized Child Laborers Teen Pregnancy Victims of Domestic Violence **Education (74.4%)** **Micro Level Socioeconomic Status (66.1%)** Income or Wealth Income Assistance Land Size Savings or Debt Food Insecurity Access to Banking **Household Composition (62.0%)** Size of Household Single Parent or Female-Headed Household Lives Alone Child-Headed Household **Employment (61.2%)** Unemployment Occupation **Housing (56.2%)** Housing Materials or Condition House Ownership House Without Necessities Housing Type Housing Price Housing Vacancy Group Housing Homelessness **Population Health Statistics (55.4%)** Migration Average Age Population Growth Total Population Birth Rate Mortality Rate Life Expectancy	**Gender or Sex (49.6%)** **Density (47.1%)** Population Density Urban or Rural Building Density **Micro Level Socioeconomic Status (42.1%)** Community Poverty or Standard of Living Gross Domestic Product or Community Finances Trade **Healthcare Infrastructure (40.5%)** Healthcare Facilities Medical Staff Health Insurance Public Health Basic Services Health Expenditure Avoidable Hospital Admissions **Transport (33.1%)** Transport Infrastructure Road Infrastructure Access to Railways, Roads or Transit (Community) Able to Get Places (Individual) **Ethnicity or Race (32.2%)** **Water and Waste (26.4%)** Water Infrastructure & Safety Waste Infrastructure and Collection **Social connection and capital (21.5%)** Relationships with Family Relationships with Friends General Relationships Emotional Support Available General Support Available to Help Relationships with Neighbours Telephone Use Ability to Give Specific Task Support Available Help Available in a Crisis Relationships with Children Community Social Support Loving Support Available Relationships with Community Relationships with Spouse **Individual Communication (20.7%)** Ability to Communicate (Oral or Written) Sensory Problems	**Disaster Preparedness (19.0%)** Access to Internet, Phone or Radio Community Disaster Resources First Responders **Marital Status (18.2%)** **Land Use (17.4%)** General Land Use Farming or Soil Use Forest Green Space Ecological Land Use **Social Engagement (15.7%)** Clubs or Community Centers Golf, Physical Leisure or Walking Church or Religion Amount of Social Engagement Volunteering Feelings Towards Social Engagement Activities Around the Home (e.g., gardening) Cards or Games Hobby, Project or Further Education Pets **Power Sources (15.7%)** Power and Electricity Infrastructure Biomass **Personal Attitudes and Expectations (10.7%)** Control Expectations of Self and Others Satisfaction with Life Attitude Towards Life Self Worth or Self Esteem Major Life Events Hope for the Future **Industry (10.7%)** Tourism or Hospitality Specific Industries (e.g. Cotton) General Industries (e.g. Primary) **Environment and Climate Events (10.7%)** Flood Extreme Weather Rainfall or Drought Landslides **Government Aptitude and Investments (7.4%)** School Infrastructure Capacity for Governance Corruption Research and Development Infrastructure **Isolation or Loneliness (6.6%)** **Health Conditions (6.6%)** Chronic Health Conditions or their Risk Factors HIV / AIDS Poor Mental Health Specific Disease Incidence Specific Disease after Flood Adherence to Medical Advice **Political Stability (5.0%)** Refugees Displaced Political Armed Conflict **Noise or Air Pollution (2.5%)**

Education was the second most common domain amongst all SVIs (74.4%) and equally prevalent in SVIs from all fields of study. The third most common domain was an item about individual level socioeconomic status. These items asked directly about income or wealth, sources of income, debt or savings, or food insecurity. This is different than macro level markers of socioeconomic status asked in 42.1% of SVIs where the questions focused on community poverty level, gross domestic product, or trade statistics per geographic region.

The least common domains were political instability and pollution. Only 6 SVIs included questions on displaced refugees or political armed conflict. Three SVIs inquired directly about noise or air pollution. Interestingly, a minority of SVIs (6.6%) included items about health conditions, most of which from SVIs used in environment, climate or disaster planning fields, including one item on diseases after a flood.

The full list of items and their frequencies are provided in Additional file 5 divided by field of study as there were differences in SVI composition across fields. For example, items about social connection and capital were more likely to be included in SVIs used in health or medicine (59.3%) than environment, climate, or disaster SVIs (10.9%). There were few SVIs in health or medicine which included items about safe water and waste disposal compared to 31.5% of environmental, climate or disaster SVIs. Education, socioeconomic status and transport were equally common domains among all SVIs.

### Outcomes

**Fig. 3:**
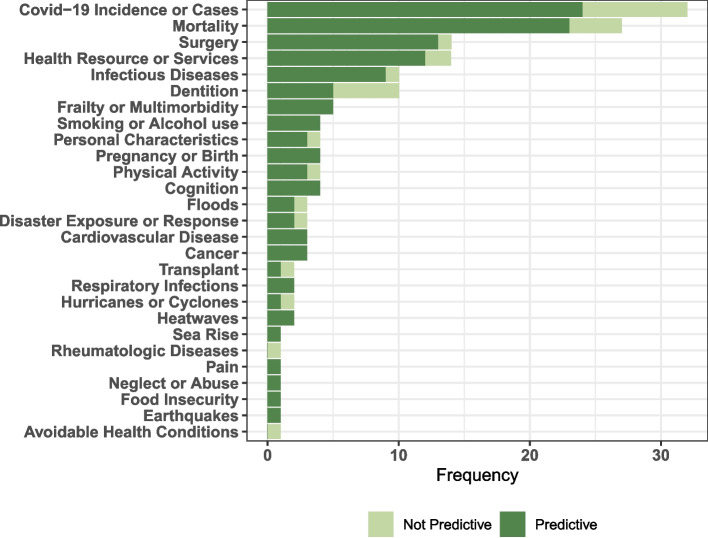
Association between SVI and outcomes among studies utilizing SVI in predictive modelling

SVIs were used to predict outcomes in 47.9% (140/292) of studies, more so in health or medicine studies (124/156) and in studies including replicated SVIs (121/174). As shown in Fig. 3, rate of Covid-19 infection or mortality was the most common outcome measure, evaluated 32 times. SVI was significantly associated with mortality in 85.1% of 27 cases. Other common outcomes studied in association with SVI were access to healthcare services or resources and surgical access or outcomes (14 times respectively). For all seven outcomes (Covid-19, mortality, surgery, healthcare services or resources, infectious disease incidence, dentition and frailty) with at least five studies, SVI significantly predicted direction of outcome in more than 75% of the studies except for the outcome of dentition.

## Discussion

In this scoping review, we provide an overview of SVIs in the literature with a focus on mapping out the composition of these indices and how they are used to predict outcomes. While there are few systematic or scoping reviews on indices of social vulnerability specifically, there are two grey literature narrative reviews on social vulnerability assessment tools by the United Nations Development Programme for climate change [27], and the US Corps of Engineers’ Institute for Water Resources [28]. These reviews have previously hypothesized that, despite heterogeneity in the indicators used, the methods for calculating SVIs are relatively similar in most situations. Our findings support that hypothesis and add a broadened scope by including SVIs in the fields of health and medicine.

We also mapped items and domains used in the composition of SVIs as one way of answering an often-debated question: which social factors should be included in an index to represent social conditions? Despite differing fields, purposes and applications of the 121 original SVIs included in our review, we found seven domains of social factors that were used in the composition of over half of the SVIs: at risk populations, education, micro (i.e., individual, family or household) level markers of socioeconomic status, household composition, employment, housing, and population health statistics. In these domains, there are items from both the individual level, the household level and population level. Individuals’ vulnerabilities cannot be separated from their systemically disadvantaged communities and our finding that one in five SVIs used mixed data sources suggest that future SVIs may be strengthened if composed of social factors reflective of vulnerabilities at the individual, family, neighbourhood and community levels.

Not mentioned in the domains above is gender or sex, despite previous consensus that this item should be included in a SVI [9]. Since it is well documented that gender and sex differences (biologically and socially) contribute to different experiences and outcomes in health [29] and disasters [30], we were surprised that less than 50% of SVIs included gender or sex. This may reflect a choice of the researchers because most items for SVIs came from census data or population-based surveys, so variables on gender or sex demographics are not readily available. There were no variables reflective of sexuality. The exclusion of sexuality as a determinant of social vulnerability can be problematic as it makes invisible the challenges faced by sexual minorities (e.g., perpetuating discrimination), masks disparities (e.g., in access to housing, healthcare access or social services) and fails to recognize the intersectionality whereby sexual minorities are often part of multiple vulnerable groups. Other commonly included SVI items that would likely be gendered depending on the context, include educational and occupational opportunities, and also marital status and living situation. We were surprised that items like single parent or female-headed household were less prevalent in the health and medicine SVIs (see Additional file 5) and overall, we had expected consideration of sex and gender-related items to be more common. While it is possible that gender or sex stratified analyses are being conducted instead of including specific items in the index, our findings suggest that many SVIs may be missing an important determinant of vulnerability. Researchers need to carefully consider how to construct their indices and choose data sources with information collected on sex and gender. The most frequent variable was a dichotomized proportion of sex or gender, reflecting previous literature describing how the dominant discourse in disaster management on sex and gender is binary, and does not account for gender minorities [30].

This paper adds to the literature in two key ways. First, our findings confirm that interest in measuring social vulnerability is increasing, especially in the health and medicine fields (Table 1). This growing trend seems to have been linked to researchers trying to understand the social and economic factors contributing to the differential impacts of the pandemic across various populations. The interest may also be tied to the rising importance of interdisciplinary research, the growing recognition of climate change’s impact on social and health inequities and the advances in available data in which to conduct social vulnerability research. We also demonstrate that when SVIs are used to measure an outcome, the outcome was overwhelming in the health and medicine fields, and the SVI was predictive. However, the SVIs in health or medicine related fields were  more often replicates than original SVIs, suggesting  health and medicine studies are employing SVIs developed for other fields of literature (e.g., SVI by the CDC/ASTAR). Social vulnerability is often context dependent and having more original indices with community specific data may be a better tool for measuring social vulnerability related to health. Second, we provide a scaffold for future researchers looking to create these original SVIs. There are many ways to choose social factors in an index from theory driven to data availability to community consultations; however there is no gold standard. Here, we provide another way of making this determination by summarizing what past SVIs have used, from most common to highly context specific (Additional file 5). A strength of our approach is that it includes items and domains that take into consideration individual capacity to recover from the impact of a hazard as well as the inability of the system to respond. Our approach also encompasses global items from literature in five different languages and incorporates items from several fields of research in keeping with the interdisciplinary nature of social vulnerability.

Whether we are evaluating risks from an adverse health event or disaster event, the social production of vulnerability should be given the same degree of importance dedicated to understanding and reducing the medical or environmental risk. Our findings show the social vulnerability index predicts many outcomes from mortality to frailty to disaster response. We also see that SVIs used globally. Unlike other measures which were developed and are more applicable to high income countries (e.g., the SVI by the CDC/ASTAR), the SVI appears adaptable and relevant to different contexts whereby original SVIs are emerging from all continents (except Antarctica). It also appears that one recent and frequent application of SVIs is for Covid-19. SVIs have been used as a research tool but also as a pragmatic policy tool to identify and support vulnerable communities through resource allocation [31]. Certain tools (i.e., the SVI by the CDC/ASTAR) that are free, easily accessible, and have complete data are most replicated and may facilitate researchers and policymakers taking an interest in social vulnerability [31]. If authors are creating SVIs, they should strive to use publicly available and free data and replicable with a simple methodology as this will reduce barriers to use of SVIs in broader research.

There remains many complexities and uncertainties for researchers hoping to employ SVIs, and our study has limitations which should inform interpretation of our findings. The choice to categorize indices into three broad categories (i.e., environment, climate or disaster, health or medicine, and other) may have resulted in loss of information or loss of opportunity to detect differences within fields. By excluding papers where social vulnerability indices were combined with other measures (i.e., weather indices), this review does miss out on other potential applications of the SVI. Furthermore, the search was very specific due to feasibility constraints of screening hundreds of full-text papers. There are undoubtedly many indices with the same underlying principle that are perhaps not called an SVI. Indices that may be made on social resilience factors were not part of the search, yet is one area of future exploration as it is unclear if an index of social strengths (i.e., a strengths-based resilience index) would yield comparable results to a social vulnerability index. Another consideration is that this review did not explicitly collect data on the methods authors used to determine inclusion of items (e.g., theory driven, data availability, factor analysis, community consultation, etc.). Nonetheless, to balance feasibility of the search, we still provide a review with a significant sample size with the inclusion of several languages.

There are several areas of future research on SVIs. First, validating and comparing different SVIs to understand their strengths and weaknesses and to identify the most appropriate indices for specific purposes is needed. Second, understanding trends in social vulnerability over time and determining what this means for building an index to represent social conditions at different stages of life is also needed. Finally, future studies should build on the recent pragmatic uses of SVIs during the Covid-19 pandemic, which used SVIs to plan and evaluate effectiveness of interventions designed to reduce social vulnerability.

## Conclusion

Identification of social vulnerability presents an opportunity to intervene to improve the lives of individuals and communities following an adverse health or disaster event. To identify social vulnerability, social vulnerability indices are commonly used. The social vulnerability indices presented here brings together multiple fields of literature and demonstrates growing interest in using these indices in health and medical literature. We also found that SVIs predicted Covid-19 cases, mortality, surgical access or outcomes and healthcare access or resources, among other outcomes. Since we predict the use of SVIs will continue to increase, we also provide a summary of domains and items common across SVIs in the literature, which provides an alternate method of constructing SVIs in the future. The social vulnerability indices presented here brings together literature from multiple fields of literature; whether in the field of disaster planning, environmental science or health care, the SVIs are composed of similar items reflecting interdisciplinary ways of thinking.

## Supplementary Information

**Additional file 1.** Preferred Reporting Items for Systematic reviews and Meta-Analyses extension for Scoping Reviews (PRISMA-ScR) Checklist.

**Additional file 2.** References of included studies.

**Additional file 3.** Characteristics, composition and outcomes of original social vulnerability indices (in white), and characteristics and outcomes replicated social vulnerability indices (in grey).

**Additional file 4.** a. Frequency and proportion of replications, in descending order. b. Geographic Distribution of SVIs.

**Additional file 5.** Items (proportion of the domain) and domains (proportion of all SVIs).

## Data Availability

The datasets used and/or analysed during the current study are available from the corresponding author on reasonable request.
