# Supplementary material for: Social vulnerability indices: a scoping review
Source: BMC Public Health. 2023 Jun 28;23:1253. doi: 10.1186/s12889-023-16097-6 (PMC10304642; doi:10.1186/s12889-023-16097-6)
Supplement: Supplementary file 2 — Additional file 2. References of included studies. [file 12889_2023_16097_MOESM2_ESM.docx]

## Additional File 2. References of included studies

**Originals**

1. Abeliansky AL, Erel D, Strulik H. Social vulnerability and aging of elderly people in the United States. SSM - Population Health. 2021 Dec;16:100924.

2. Adger WN, Vincent K. Uncertainty in adaptive capacity. Comptes Rendus Geoscience. 2005;337(4):399–410.

3. Aksha SK, Juran L, Resler LM, Zhang Y. An Analysis of Social Vulnerability to Natural Hazards in Nepal Using a Modified Social Vulnerability Index. Int J Disaster Risk Sci. 2019 Mar;10(1):103–16.

4. Andrew MK, Keefe JM. Social vulnerability from a social ecology perspective: a cohort study of older adults from the National Population Health Survey of Canada. BMC Geriatr. 2014 Dec;14(1):90.

5. Andrew MK, Mitnitski A, Rockwood K. Social Vulnerability, Frailty and Mortality in Elderly People. PLoS ONE. 2008;3(5).

6. Armaș I, Gavriș A. Social vulnerability assessment using spatial multi-criteria analysis (SEVI model) and the Social Vulnerability Index (SoVI model) – a case study for Bucharest, Romania. Nat Hazards Earth Syst Sci. 2013 Jun 18;13(6):1481–99.

7. Armstrong JJ, Andrew MK, Mitnitski A, Launer LJ, White LR, Rockwood K. Social vulnerability and survival across levels of frailty in the Honolulu-Asia Aging Study. Age and Ageing. 2015 Jul;44(4):709–12.

8. Aroca-Jimenez E, Bodoque JM, Garcia JA, Diez-Herrero A. Construction of an integrated social vulnerability index in urban areas prone to flash flooding. Nat Hazards Earth Syst Sci. 2017 Sep 15;17(9):1541–57.

9. Aroca-Jiménez E, Bodoque JM, García JA. How to construct and validate an Integrated Socio-Economic Vulnerability Index: Implementation at regional scale in urban areas prone to flash flooding. Science of The Total Environment. 2020 Dec;746:140905.

10. Badmos BK, Adenle AA, Agodzo SK, Villamor GB, Asare-Kyei DK, Amadou LM, et al. Micro-level social vulnerability assessment towards climate change adaptation in semi-arid Ghana, West Africa. Environ Dev Sustain. 2018 Oct;20(5):2261–79.

11. Ballesteros C, Esteves LS. Integrated Assessment of Coastal Exposure and Social Vulnerability to Coastal Hazards in East Africa. Estuaries and Coasts. 2021 Dec;44(8):2056–72.

12. Berrouet L, Villegas-Palacio C, Botero V. A social vulnerability index to changes in ecosystem services provision at local scale: A methodological approach. Environmental Science & Policy. 2019 Mar;93:158–71.

13. Bjarnadottir S, Li Y, Stewart MG. Social vulnerability index for coastal communities at risk to hurricane hazard and a changing climate. Nat Hazards. 2011 Nov;59(2):1055–75.

14. Bronfman NC, Repetto PB, Guerrero N, Castañeda JV, Cisternas PC. Temporal evolution in social vulnerability to natural hazards in Chile. Nat Hazards. 2021 Jun;107(2):1757–84.

15. Bunt S, Steverink N, Andrew MK, Schans CP van der, Hobbelen H. Cross-Cultural Adaptation of the Social Vulnerability Index for Use in the Dutch Context. IJERPH. 2017 Nov 14;14(11):1387.

16. Burton C, Cutter S. Levee Failures and Social Vulnerability in the Sacramento-San Joaquin Delta Area, California. Natural Hazards Review. 2008;9(3).

17. Cerami C, Canevelli M, Santi GC, Galandra C, Dodich A, Cappa SF, et al. Identifying Frail Populations for Disease Risk Prediction and Intervention Planning in the Covid-19 Era: A Focus on Social Isolation and Vulnerability. Front Psychiatry. 2021 Aug 20;12:626682.

18. Chakraborty J, Tobin GA, Montz BE. Population Evacuation: Assessing Spatial Variability in Geophysical Risk and Social Vulnerability to Natural Hazards. Nat Hazards Rev. 2005 Feb;6(1):23–33.

19. Chau PH, Gusmano MK, Cheng JOY, Cheung SH, Woo J. Social Vulnerability Index for the Older People—Hong Kong and New York City as Examples. J Urban Health. 2014 Dec;91(6):1048–64.

20. Chen W, Cutter SL, Emrich CT, Shi P. Measuring social vulnerability to natural hazards in the Yangtze River Delta region, China. Int J Disaster Risk Sci. 2013 Dec;4(4):169–81.

21. Chen Y, Ye Z, Liu H, Chen R, Liu Z, Liu H. A GIS-Based Approach for Flood Risk Zoning by Combining Social Vulnerability and Flood Susceptibility: A Case Study of Nanjing, China. IJERPH. 2021 Nov 4;18(21):11597.

22. Cumberbatch J, Drakes C, Mackey T, Nagdee M, Wood J, Degia AK, et al. Social Vulnerability Index: Barbados – A Case Study. Coastal Management. 2020 Sep 2;48(5):505–26.

23. Cutler MJ, Marlon JR, Howe PD, Leiserowitz A. The Influence of Political Ideology and Socioeconomic Vulnerability on Perceived Health Risks of Heat Waves in the Context of Climate Change. Weather, Climate, and Society. 2018 Oct 1;10(4):731–46.

24. Cutter SL, Boruff BJ, Shirley WL. Social Vulnerability to Environmental Hazards. Social Science Quarterly. 2003 Jun;84(2):242–61.

25. de Loyola Hummell BM, Cutter SL, Emrich CT. Social Vulnerability to Natural Hazards in Brazil. Int J Disaster Risk Sci. 2016 Jun;7(2):111–22.

26. de Medeiros MD, de Almeida LQ. VULNERABILIDADE SOCIOAMBIENTAL NO MUNICÍPIO DE NATAL, RN, BR. REDE-Revista Eletrônica do PRODEMA. 2016;9(2):15.

27. Denver GEA, Sciegaj M, Wade TE, Lofton TC. Creation of a social vulnerability index for justice in health planning. Fam Community Health. 1988;10(4):23–32.

28. Dintwa KF, Letamo G, Navaneetham K. Measuring social vulnerability to natural hazards at the district level in Botswana. Jàmbá Journal of Disaster Risk Studies [Internet]. 2019 May 6 [cited 2022 Mar 7];11(1). Available from: http://www.jamba.org.za/index.php/JAMBA/article/view/447

29. Dintwa KF, Letamo G, Navaneetham K. Quantifying social vulnerability to natural hazards in Botswana: An application of cutter model. International Journal of Disaster Risk Reduction. 2019 Jul;37:101189.

30. Dossou JF, Li XX, Kouhoundji NK, Vissin EW. Impact of Agriculture on the Oueme Basin in Benin. Water Air Soil Pollut. 2021 Dec;232(12):479.

31. Felsenstein D, Lichter M. Social and economic vulnerability of coastal communities to sea-level rise and extreme flooding. Nat Hazards. 2014 Mar;71(1):463–91.

32. Flanagan BE, Gregory EW, Hallisey EJ, Heitgerd JL, Lewis B. A Social Vulnerability Index for Disaster Management. Journal of Homeland Security and Emergency Management [Internet]. 2011 Jan 5 [cited 2022 Jan 26];8(1). Available from: https://www.degruyter.com/document/doi/10.2202/1547-7355.1792/html

33. Fraser T. Japanese social capital and social vulnerability indices: Measuring drivers of community resilience 2000–2017. International Journal of Disaster Risk Reduction. 2021 Jan;52:101965.

34. Frigerio I, Carnelli F, Cabinio M, De Amicis M. Spatiotemporal Pattern of Social Vulnerability in Italy. Int J Disaster Risk Sci. 2018 Jun;9(2):249–62.

35. Frigerio I, De Amicis M. Mapping social vulnerability to natural hazards in Italy: A suitable tool for risk mitigation strategies. Environmental Science & Policy. 2016 Sep;63:187–96.

36. Frigerio I, Zanini F, Mattavelli M, De Amicis M. Understanding the interacting factors that influence social vulnerability: a case study of the 2016 central Italy earthquake. Disasters. 2019 Oct;43(4):867–90.

37. Gautam D. Assessment of social vulnerability to natural hazards in Nepal. Nat Hazards Earth Syst Sci. 2017 Dec 15;17(12):2313–20.

38. Ge Y, Dou W, Dai J. A New Approach to Identify Social Vulnerability to Climate Change in the Yangtze River Delta. Sustainability. 2017 Dec 4;9(12):2236.

39. Ge Y, Dou W, Gu Z, Qian X, Wang J, Xu W, et al. Assessment of social vulnerability to natural hazards in the Yangtze River Delta, China. Stoch Environ Res Risk Assess. 2013 Dec;27(8):1899–908.

40. Ge Y, Dou W, Zhang H. A New Framework for Understanding Urban Social Vulnerability from a Network Perspective. Sustainability. 2017 Sep 26;9(10):1723.

41. Ge Y, Yang G, Chen Y, Dou W. Examining Social Vulnerability and Inequality: A Joint Analysis through a Connectivity Lens in the Urban Agglomerations of China. Sustainability. 2019 Feb 17;11(4):1042.

42. Giovene di Girasole E, Cannatella D. Social Vulnerability to Natural Hazards in Urban Systems. An Application in Santo Domingo (Dominican Republic). Sustainability. 2017 Nov 7;9(11):2043.

43. Godin J, Theou O, Black K, McNeil SA, Andrew MK. Long-Term Care Admissions Following Hospitalization: The Role of Social Vulnerability. Healthcare. 2019 Jul 15;7(3):91.

44. Grasso M, Moneo M, Arena M. Assessing social vulnerability to climate change in Samoa. Reg Environ Change. 2014 Aug;14(4):1329–41.

45. Gu H, Du S, Liao B, Wen J, Wang C, Chen R, et al. A hierarchical pattern of urban social vulnerability in Shanghai, China and its implications for risk management. Sustainable Cities and Society. 2018 Aug;41:170–9.

46. Guo Y, Chau PPH, Chang Q, Woo J, Wong M, Yip PSF. The geography of suicide in older adults in Hong Kong: An ecological study. Int J Geriatr Psychiatry. 2020 Jan;35(1):99–112.

47. Gupta AK, Negi M, Nandy S, Kumar M, Singh V, Valente D, et al. Mapping socio-environmental vulnerability to climate change in different altitude zones in the Indian Himalayas. Ecological Indicators. 2020 Feb;109:105787.

48. Hazards and Vulnerability Research Institute at the University of South Carolina. SoVI®: Social Vulnerability Index for the United States 2010–14 [Internet]. 2016 [cited 2022 Jan 27]. Available from: https://www.sc.edu/study/colleges_schools/artsandsciences/centers_and_institutes/hvri/data_and_resources/sovi/

49. Hofflinger A, Somos-Valenzuela MA, Vallejos-Romero A. Response time to flood events using a social vulnerability index (ReTSVI). Nat Hazards Earth Syst Sci. 2019 Jan 28;19(1):251–67.

50. Instituto de Pesquisa Econômica Aplicada. Atlas da Vulnerabilidade Social nos Municípios Brasileiros [Internet]. 2015. Available from: https://www.ipea.gov.br/portal/index.php?option=com_content&view=article&id=26118

51. Jagarnath M, Thambiran T, Gebreslasie M. Heat stress risk and vulnerability under climate change in Durban metropolitan, South Africa—identifying urban planning priorities for adaptation. Climatic Change. 2020 Nov;163(2):807–29.

52. Kamiohkawa S, Maruyama A, Sanyu Consultants Inc., 1-13-17 Kita-Otsuka, Toshima ward, Tokyo 170-0004, Japan, Buot I, Buot M. Index Assessment of Household Social Vulnerability to Climate Change: A Case Study of Laguna Province, Philippines. JESAM. 2021 Jun 30;24(1):68–76.

53. Karunarathne AY, Lee G. Developing a multi-facet social vulnerability measure for flood disasters at the micro-level assessment. International Journal of Disaster Risk Reduction. 2020 Oct;49:101679.

54. Kim J, Gim THT. Assessment of social vulnerability to floods on Java, Indonesia. Nat Hazards. 2020 May;102(1):101–14.

55. Kirby RH, Reams MA, Lam NSN, Zou L, Dekker GGJ, Fundter DQP. Assessing Social Vulnerability to Flood Hazards in the Dutch Province of Zeeland. Int J Disaster Risk Sci. 2019 Jun;10(2):233–43.

56. Koks EE, Jongman B, Husby TG, Botzen WJW. Combining hazard, exposure and social vulnerability to provide lessons for flood risk management. Environmental Science & Policy. 2015 Mar;47:42–52.

57. Kumar D, Kumar Bhattacharjya R. Study of Integrated Social Vulnerability Index SoVI _int_ of Hilly Region of Uttarakhand, India. Environmental and Climate Technologies. 2020 Jan 1;24(1):105–22.

58. Lawal O, Arokoyu SB. Modelling social vulnerability in sub-Saharan West Africa using a geographical information system. Jàmbá: Journal of Disaster Risk Studies. 2015 Feb 27;7(1):11 pages.

59. Lee YJ. Social vulnerability indicators as a sustainable planning tool. Environmental Impact Assessment Review. 2014 Jan;44:31–42.

60. Letsie MM, Grab SW. Assessment of Social Vulnerability to Natural Hazards in the Mountain Kingdom of Lesotho. Mountain Research and Development. 2015 May;35(2):115–25.

61. Lin WY, Hung CT. Applying spatial clustering analysis to a township-level social vulnerability assessment in Taiwan. Geomatics, Natural Hazards and Risk. 2016 Sep 2;7(5):1659–76.

62. Liu D, Li Y. Social vulnerability of rural households to flood hazards in western mountainous regions of Henan province, China. Nat Hazards Earth Syst Sci. 2016 May 13;16(5):1123–34.

63. Liu HY, Peng LN, Lee WJ, Chou MY, Liang CK, Hsiao FY, et al. Differential moderation effects of ApoE and 5-HTTLPR genotypes on social vulnerability in predicting mortality among community-dwelling middle-aged and older adults: a nationwide population-based study. Aging. 2021 Oct 15;13(19):23348–60.

64. Lixin Y, Ke C, Xiaoying C, Yueling S, Xiaoqing C, Ye H. Analysis of social vulnerability of residential community to hazards in Tianjin, China. Nat Hazards. 2017 Jun;87(2):1223–43.

65. Lixin Y, Xi Z, Lingling G, Dong Z. Analysis of social vulnerability to hazards in China. Environ Earth Sci. 2014 Apr;71(7):3109–17.

66. Maharani YN, Lee S. Assessment of social vulnerability to natural hazards in South Korea: case study for typhoon hazard. Spat Inf Res. 2017 Feb;25(1):99–116.

67. Martínez C, Cienfuegos R, Inzunza S, Urrutia A, Guerrero N. Worst-case tsunami scenario in Cartagena Bay, central Chile: Challenges for coastal risk management. Ocean & Coastal Management. 2020 Mar;185:105060.

68. Mavhura E, Manyena B, Collins AE. An approach for measuring social vulnerability in context: The case of flood hazards in Muzarabani district, Zimbabwe. Geoforum. 2017 Nov;86:103–17.

69. Mavromatidi A, Briche E, Claeys C. Mapping and analyzing socio-environmental vulnerability to coastal hazards induced by climate change: An application to coastal Mediterranean cities in France. Cities. 2018 Feb;72:189–200.

70. Moyano D, Forclaz Z, Chaparro RM, Hernández-Vásquez A, Perovic NR. Multilevel analysis concerning the relationship between social vulnerability and the healthy use of leisure time in children and adolescents in Argentina: A national population-based study. F1000Res. 2021 May 7;7:1619.

71. Nahas M, Ribeiro C, Esteves O, Moscovitch S, Martins V. The map of social exclusion in Belo Horizonte: Methodology of building an urban management tool. Cad Cienc Soc. 2000;7(75–88).

72. Nelson KS, Abkowitz MD, Camp JV. A method for creating high resolution maps of social vulnerability in the context of environmental hazards. Applied Geography. 2015 Sep;63:89–100.

73. Nguyen CV, Horne R, Fien J, Cheong F. Assessment of social vulnerability to climate change at the local scale: development and application of a Social Vulnerability Index. Climatic Change. 2017 Aug;143(3–4):355–70.

74. Nguyen T, Ngangue P, Bouhali T, Ryan B, Stewart M, Fortin M. Social Vulnerability in Patients with Multimorbidity: A Cross-Sectional Analysis. IJERPH. 2019 Apr 8;16(7):1244.

75. Nicholson D, Vanli OA, Jung S, Ozguven EE. A spatial regression and clustering method for developing place-specific social vulnerability indices using census and social media data. International Journal of Disaster Risk Reduction. 2019 Aug;38:101224.

76. Ogie R, Pradhan B. Social vulnerability to natural hazards in Wollongong: Comparing strength-based and traditional methods. The Australian Journal of Emergency Management. 2020;35(1):60–8.

77. Oulahen G, Mortsch L, Tang K, Harford D. Unequal Vulnerability to Flood Hazards: “Ground Truthing” a Social Vulnerability Index of Five Municipalities in Metro Vancouver, Canada. Annals of the Association of American Geographers. 2015 May 4;105(3):473–95.

78. Prabhu S, Wanje G, Oyaro B, Otieno F, Mandaliya K, Jaoko W, et al. Adaptation of a social vulnerability index for measuring social frailty among East African women. BMC Public Health. 2022 Dec;22(1):167.

79. Reckien D. What is in an index? Construction method, data metric, and weighting scheme determine the outcome of composite social vulnerability indices in New York City. Reg Environ Change. 2018 Jun;18(5):1439–51.

80. Rifat SAA, Liu W. One year into the pandemic: the impacts of social vulnerability on COVID-19 outcomes and urban–rural differences in the conterminous United States. International Journal of Environmental Health Research. 2022 Dec 2;32(12):2601–19.

81. Roder G, Sofia G, Wu Z, Tarolli P. Assessment of Social Vulnerability to Floods in the Floodplain of Northern Italy. Weather, Climate, and Society. 2017 Oct 1;9(4):717–37.

82. Rodriquez C, Monteiro R, Ceresa P. Assessing Seismic Social Vulnerability in Urban Centers — the Case-Study of Nablus, Palestine. International Journal of Architectural Heritage. 2018 Nov 17;12(7–8):1216–30.

83. Roncancio DJ, Cutter SL, Nardocci AC. Social vulnerability in Colombia. International Journal of Disaster Risk Reduction. 2020 Nov;50:101872.

84. Sánchez-Garrido N, Aguilar-Navarro SG, Ávila-Funes JA, Theou O, Andrew M, Pérez-Zepeda MU. The Social Vulnerability Index, Mortality and Disability in Mexican Middle-Aged and Older Adults. Geriatrics. 2021 Mar 8;6(1):24.

85. Schmitz M, Rudas S. Vienna vulnerability index: An index for establishing the degree of risk to the mentally ill. The European journal of psychiatry. 2991;15(2):117–27.

86. Shaji J. Evaluating social vulnerability of people inhabiting a tropical coast in Kerala, south west coast of India. International Journal of Disaster Risk Reduction. 2021 Apr;56:102130.

87. Sharma A, Woodruff S, Budhathoki M, Hamlet AF, Chen F, Fernando HJS. Role of green roofs in reducing heat stress in vulnerable urban communities—a multidisciplinary approach. Environ Res Lett. 2018 Sep 5;13(9):094011.

88. Sharma G, Patil GR. Public transit accessibility approach to understand the equity for public healthcare services: A case study of Greater Mumbai. Journal of Transport Geography. 2021 Jun;94:103123.

89. Shega JW, Andrew M, Hemmerich J, Cagney KA, Ersek M, Weiner DK, et al. The Relationship of Pain and Cognitive Impairment with Social Vulnerability—An Analysis of the Canadian Study of Health and Aging. Pain Med. 2012 Feb;13(2):190–7.

90. Siagian TH, Purhadi P, Suhartono S, Ritonga H. Social vulnerability to natural hazards in Indonesia: driving factors and policy implications. Nat Hazards. 2014 Jan;70(2):1603–17.

91. Snyder BF, Parks V. Spatial variation in socio-ecological vulnerability to Covid-19 in the contiguous United States. Health & Place. 2020 Nov;66:102471.

92. Solangaarachchi D, Griffin AL, Doherty MD. Social vulnerability in the context of bushfire risk at the urban-bush interface in Sydney: a case study of the Blue Mountains and Ku-ring-gai local council areas. Nat Hazards. 2012 Nov;64(2):1873–98.

93. Stanturf JA, Goodrick SL, Warren ML, Charnley S, Stegall CM. Social Vulnerability and Ebola Virus Disease in Rural Liberia. Sankoh OA, editor. PLoS ONE. 2015 Sep 1;10(9):e0137208.

94. Su S, Pi J, Wan C, Li H, Xiao R, Li B. Categorizing social vulnerability patterns in Chinese coastal cities. Ocean & Coastal Management. 2015 Nov;116:1–8.

95. Tanir T, de Lima A de S, de A. Coelho G, Uzun S, Cassalho F, Ferreira CM. Assessing the spatiotemporal socioeconomic flood vulnerability of agricultural communities in the Potomac River Watershed. Nat Hazards. 2021 Aug;108(1):225–51.

96. Tascón-González L, Ferrer-Julià M, Ruiz M, García-Meléndez E. Social Vulnerability Assessment for Flood Risk Analysis. Water. 2020 Feb 17;12(2):558.

97. Tasnuva A, Hossain MdR, Salam R, Islam ARMdT, Patwary MM, Ibrahim SM. Employing social vulnerability index to assess household social vulnerability of natural hazards: an evidence from southwest coastal Bangladesh. Environ Dev Sustain. 2021 Jul;23(7):10223–45.

98. Tate E, Strong A, Kraus T, Xiong H. Flood recovery and property acquisition in Cedar Rapids, Iowa. Nat Hazards. 2016 Feb;80(3):2055–79.

99. Toké NA, Boone CG, Arrowsmith JR. Fault zone regulation, seismic hazard, and social vulnerability in Los Angeles California: Hazard or urban amenity? Earth’s Future. 2014 Sep;2(9):440–57.

100. Török I. Assessment of social vulnerability to natural hazards in Romania. Carpathian Journal of Earth and Environmental Sciences. 2017;12(2):549–62.

101. Török I. Qualitative Assessment of Social Vulnerability to Flood Hazards in Romania. Sustainability. 2018 Oct 19;10(10):3780.

102. Török I, Croitoru AE, Man TC. Assessing the Impact of Extreme Temperature Conditions on Social Vulnerability. Sustainability. 2021 Jul 30;13(15):8510.

103. Tragaki A, Gallousi C, Karymbalis E. Coastal Hazard Vulnerability Assessment Based on Geomorphic, Oceanographic and Demographic Parameters: The Case of the Peloponnese (Southern Greece). Land. 2018 May 1;7(2):56.

104. Varughese RA, Theou O, Li Y, Huang X, Chowdhury N, Famure O, et al. Cumulative Deficits Frailty Index Predicts Outcomes for Solid Organ Transplant Candidates. Transplantation Direct. 2021 Feb 22;7(3):e677.

105. Vincent K. Creating an index of social vulnerability for Africa. Norwich, UK: Tyndall Centre for Climate Change Research, University of East Anglia; 2004. Report No.: Working Paper 56.

106. Wallace LMK, Theou O, Pena F, Rockwood K, Andrew MK. Social vulnerability as a predictor of mortality and disability: cross-country differences in the survey of health, aging, and retirement in Europe (SHARE). Aging Clin Exp Res. 2015 Jun;27(3):365–72.

107. Waly NM, Ayad HM, Saadallah DM. Assessment of spatiotemporal patterns of social vulnerability: A tool to resilient urban development Alexandria, Egypt. Ain Shams Engineering Journal. 2021 Mar;12(1):1059–72.

108. Ware LJ, Kim AW, Prioreschi A, Nyati LH, Taljaard W, Draper CE, et al. Social vulnerability, parity and food insecurity in urban South African young women: the healthy life trajectories initiative (HeLTI) study. J Public Health Pol. 2021 Sep;42(3):373–89.

109. Yang S, He S, Du J, Sun X. Screening of social vulnerability to natural hazards in China. Nat Hazards. 2015 Mar;76(1):1–18.

110. Yuan B, Wang Z, Li J. Social vulnerability and infant mortality in space dimension: an investigation of the world’s most underdeveloped West Africa coastal area. IJHRH. 2020 Jan 31;13(3):239–48.

111. Zarghami SA, Dumrak J. A system dynamics model for social vulnerability to natural disasters: Disaster risk assessment of an Australian city. International Journal of Disaster Risk Reduction. 2021 Jun;60:102258.

112. Zebardast E. Constructing a social vulnerability index to earthquake hazards using a hybrid factor analysis and analytic network process (F’ANP) model. Nat Hazards. 2013 Feb;65(3):1331–59.

113. Zhang N, Huang H. Social vulnerability for public safety: A case study of Beijing, China. Chin Sci Bull. 2013 Jul;58(19):2387–94.

114. Zhang W, Xu X, Chen X. Social vulnerability assessment of earthquake disaster based on the catastrophe progression method: A Sichuan Province case study. International Journal of Disaster Risk Reduction. 2017 Sep;24:361–72.

115. Zhang YL, You WJ. Social vulnerability to floods: a case study of Huaihe River Basin. Nat Hazards. 2014 Apr;71(3):2113–25.

116. Zhou Y, Li N, Wu W, Wu J, Shi P. Local Spatial and Temporal Factors Influencing Population and Societal Vulnerability to Natural Disasters: Population and Societal Vulnerability to Natural Disasters. Risk Analysis. 2014 Apr;34(4):614–39.

117. Zhu Q, Liu T, Lin H, Xiao J, Luo Y, Zeng W, et al. The spatial distribution of health vulnerability to heat waves in Guangdong Province, China. Global Health Action. 2014 Dec;7(1):25051.

118. São Paulo Índice Paulista de Vulnerabilidade Social. Fundação Seade. Distribuição da população, segundo grupos do IPVS. São Paulo: Fundação Seade; 2010.

**Replicates**

1. Abbas A, Madison Hyer J, Pawlik TM. Race/Ethnicity and County-Level Social Vulnerability Impact Hospice Utilization Among Patients Undergoing Cancer Surgery. Ann Surg Oncol. 2021 Apr;28(4):1918–26.

2. Alem D, Bonilla-Londono HF, Barbosa-Povoa AP, Relvas S, Ferreira D, Moreno A. Building disaster preparedness and response capacity in humanitarian supply chains using the Social Vulnerability Index. European Journal of Operational Research. 2021 Jul;292(1):250–75.

3. Almeida Andrade L, Silva da Paz W, Fontes Lima AGC, da Conceição Araújo D, Duque AM, Peixoto MVS, et al. Spatiotemporal Pattern of COVID-19–Related Mortality during the First Year of the Pandemic in Brazil: A Population-based Study in a Region of High Social Vulnerability. The American Journal of Tropical Medicine and Hygiene. 2022 Jan 5;106(1):132–41.

4. Alves YM, Berra TZ, Alves LS, de Assis IS, Arcoverde MAM, Ramos ACV, et al. Risk areas for tuberculosis among children and their inequalities in a city from Southeast Brazil. BMC Pediatr. 2020 Dec;20(1):462.

5. An R, Xiang X. Social Vulnerability and Leisure-time Physical Inactivity among US Adults. Am J Hlth Behav. 2015 Nov 1;39(6):751–60.

6. Anderson CC, Hagenlocher M, Renaud FG, Sebesvari Z, Cutter SL, Emrich CT. Comparing index-based vulnerability assessments in the Mississippi Delta: Implications of contrasting theories, indicators, and aggregation methodologies. International Journal of Disaster Risk Reduction. 2019 Oct;39:101128.

7. Andrade AWF, Souza CDF, Carmo RF. Temporal and spatial trends in human visceral leishmaniasis in an endemic area in Northeast Brazil and their association with social vulnerability. Transactions of The Royal Society of Tropical Medicine and Hygiene. 2022 May 2;116(5):469–78.

8. Andrew MK, Rockwood K. Social vulnerability predicts cognitive decline in a prospective cohort of older Canadians. Alzheimer’s & Dementia. 2010 Jul;6(4):319-325.e1.

9. Angelidou A, Sullivan K, Melvin PR, Shui JE, Goldfarb IT, Bartolome R, et al. Association of Maternal Perinatal SARS-CoV-2 Infection With Neonatal Outcomes During the COVID-19 Pandemic in Massachusetts. JAMA Netw Open. 2021 Apr 23;4(4):e217523.

10. Arling G, Blaser M, Cailas M, Canar JR, Cooper B, Flax-Hatch J, et al. A Data Driven Approach for Prioritizing COVID-19 Vaccinations in the Midwestern United States. OJPHI [Internet]. 2021 Mar 21 [cited 2022 Dec 22];13(1). Available from: https://journals.uic.edu/ojs/index.php/ojphi/article/view/11621

11. Armaş I, Gavriş A. Census-based Social Vulnerability Assessment for Bucharest. Procedia Environmental Sciences. 2016;32:138–46.

12. Armstrong JJ, Mitnitski A, Andrew MK, Launer LJ, White LR, Rockwood K. Cumulative impact of health deficits, social vulnerabilities, and protective factors on cognitive dynamics in late life: a multistate modeling approach. Alz Res Therapy. 2015 Dec;7(1):38.

13. Arroyo LH, Yamamura M, Protti-Zanatta ST, Fusco APB, Palha PF, Ramos ACV, et al. Identificação de áreas de risco para a transmissão da tuberculose no município de São Carlos, São Paulo, 2008 a 2013*. Epidemiologia e Serviços de Saúde. 2017 Jul;26(3):525–34.

14. Azap RA, Diaz A, Hyer JM, Tsilimigras DI, Mirdad RS, Ejaz A, et al. Impact of Race/Ethnicity and County-Level Vulnerability on Receipt of Surgery Among Older Medicare Beneficiaries With the Diagnosis of Early Pancreatic Cancer. Ann Surg Oncol. 2021 Oct;28(11):6309–16.

15. Azap RA, Hyer JM, Diaz A, Paredes AZ, Pawlik TM. Association of County-Level Vulnerability, Patient-Level Race/Ethnicity, and Receipt of Surgery for Early-Stage Hepatocellular Carcinoma. JAMA Surg. 2021 Feb 1;156(2):197.

16. Azap RA, Paredes AZ, Diaz A, Hyer JM, Pawlik TM. The association of neighborhood social vulnerability with surgical textbook outcomes among patients undergoing hepatopancreatic surgery. Surgery. 2020 Nov;168(5):868–75.

17. Baggio JAO, Machado MF, Carmo RF do, Armstrong A da C, Santos AD dos, Souza CDF de. COVID-19 in Brazil: spatial risk, social vulnerability, human development, clinical manifestations and predictors of mortality – a retrospective study with data from 59 695 individuals. Epidemiol Infect. 2021;149:e100.

18. Baquero OS, Ferreira F, Robis M, Neto JSF, Onell JA. Bayesian spatial models of the association between interpersonal violence, animal abuse and social vulnerability in São Paulo, Brazil. Preventive Veterinary Medicine. 2018 Apr;152:48–55.

19. Barboza GE. The Geography of Child Maltreatment: A Spatiotemporal Analysis Using Bayesian Hierarchical Analysis With Integrated Nested Laplace Approximation. J Interpers Violence. 2019 Jan;34(1):50–80.

20. Barry V, Dasgupta S, Weller DL, Kriss JL, Cadwell BL, Rose C, et al. Patterns in COVID-19 Vaccination Coverage, by Social Vulnerability and Urbanicity — United States, December 14, 2020–May 1, 2021. MMWR Morb Mortal Wkly Rep. 2021 Jun 4;70(22):818–24.

21. Basile Ibrahim B, Barcelona V, Condon EM, Crusto CA, Taylor JY. The Association Between Neighborhood Social Vulnerability and Cardiovascular Health Risk Among Black/African American Women in the InterGEN Study. Nursing Research. 2021 Sep;70(5S):S3–12.

22. Bendo CB, Paiva SM, Torres CS, Oliveira AC, Goursand D, Pordeus IA, et al. Association between treated/untreated traumatic dental injuries and impact on quality of life of Brazilian schoolchildren. Health Qual Life Outcomes. 2010 Dec;8(1):114.

23. Bendo CB, Paiva SM, Oliveira AC, Goursand D, Torres CS, Pordeus IA, et al. Prevalence and associated factors of traumatic dental injuries in Brazilian schoolchildren: Associated factors of traumatic dental injuries. Journal of Public Health Dentistry. 2010 Sep;70(4):313–8.

24. Bendo C, Vale M, Figueiredo L, Pordeus I, Paiva S. Social Vulnerability and Traumatic Dental Injury among Brazilian Schoolchildren: A Population-Based Study. IJERPH. 2012 Nov 22;9(12):4278–91.

25. Benin AL, Soe MM, Edwards JR, Bagchi S, Link-Gelles R, Schrag SJ, et al. Ecological Analysis of the Decline in Incidence Rates of COVID-19 Among Nursing Home Residents Associated with Vaccination, United States, December 2020-January 2021. Journal of the American Medical Directors Association. 2021 Oct;22(10):2009–15.

26. Berra TZ, Queiroz AAR de, Yamamura M, Arroyo LH, Garcia MC da C, Popolin MP, et al. Spatial risk of tuberculosis mortality and social vulnerability in Northeast Brazil. Rev Soc Bras Med Trop. 2017 Sep;50(5):693–7.

27. Biggs EN, Maloney PM, Rung AL, Peters ES, Robinson WT. The Relationship Between Social Vulnerability and COVID-19 Incidence Among Louisiana Census Tracts. Front Public Health. 2021 Jan 20;8:617976.

28. Bilal U, Tabb LP, Barber S, Diez Roux AV. Spatial Inequities in COVID-19 Testing, Positivity, Confirmed Cases, and Mortality in 3 U.S. Cities: An Ecological Study. Ann Intern Med. 2021 Jul;174(7):936–44.

29. Bogart LM, Dong L, Gandhi P, Klein DJ, Smith TL, Ryan S, et al. COVID-19 Vaccine Intentions and Mistrust in a National Sample of Black Americans. Journal of the National Medical Association. 2022 Jan;113(6):599–611.

30. Bozorgi P, Eberth JM, Eidson JP, Porter DE. Facility Attractiveness and Social Vulnerability Impacts on Spatial Accessibility to Opioid Treatment Programs in South Carolina. IJERPH. 2021 Apr 16;18(8):4246.

31. Brito ACM, Bezerra IM, Cavalcante D de FB, Pereira AC, Vieira V, Montezuma MF, et al. Dental caries experience and associated factors in 12-year-old-children: a population based-study. Braz oral res. 2020;34:e010.

32. Bruckhaus AA, Abedi A, Salehi S, Pickering TA, Zhang Y, Martinez A, et al. COVID-19 Vaccination Dynamics in the US: Coverage Velocity and Carrying Capacity Based on Socio-demographic Vulnerability Indices in California. J Immigrant Minority Health. 2022 Feb;24(1):18–30.

33. Burton C. Social Vulnerability and Hurricane Impact Modeling. Natural Hazards Review. 2010;11(2).

34. Carmichael H, Moore A, Steward L, Velopulos CG. Using the Social Vulnerability Index to Examine Local Disparities in Emergent and Elective Cholecystectomy. Journal of Surgical Research. 2019 Nov;243:160–4.

35. Carmichael H, Moore A, Steward L, Velopulos CG. Disparities in Emergency Versus Elective Surgery: Comparing Measures of Neighborhood Social Vulnerability. Journal of Surgical Research. 2020 Dec;256:397–403.

36. Carter AJ, Reed RD, Kale AC, Qu H, Kumar V, Hanaway MJ, et al. Impact of Social Vulnerability on Access to Educational Programming Designed to Enhance Living Donation. Prog Transpl. 2021 Dec;31(4):305–13.

37. Chang JE, Lai AY, Gupta A, Nguyen AM, Berry CA, Shelley DR. Rapid Transition to Telehealth and the Digital Divide: Implications for Primary Care Access and Equity in a Post‐COVID Era. The Milbank Quarterly. 2021 Jun;99(2):340–68.

38. Crook HL, Zhao AT, Saunders RS. Analysis of Medicare Advantage Plans’ Supplemental Benefits and Variation by County. JAMA Netw Open. 2021 Jun 23;4(6):e2114359.

39. Cunningham S, Schuldt S, Chini C, Delorit J. A simulation–optimization framework for post-disaster allocation of mental health resources. Nat Hazards Earth Syst Sci. 2021 Dec 21;21(12):3843–62.

40. Curi RLC, Gasalla MA. Social Vulnerability and Human Development of Brazilian Coastal Populations. Front Ecol Evol. 2021 Jul 29;9:664272.

41. Cutter SL, Emrich CT, Morath DP, Dunning CM. Integrating social vulnerability into federal flood risk management planning: Social vulnerability and flood risk management planning. J Flood Risk Manage. 2013 Dec;6(4):332–44.

42. da Cunha IP, Pereira AC, Frias AC, Vieira V, de Castro Meneghim M, Batista MJ, et al. Social vulnerability and factors associated with oral impact on daily performance among adolescents. Health Qual Life Outcomes. 2017 Dec;15(1):173.

43. Dalmacy DM, Tsilimigras DI, Hyer JM, Paro A, Diaz A, Pawlik TM. Social vulnerability and fragmentation of postoperative surgical care among patients undergoing hepatopancreatic surgery. Surgery. 2022 Apr;171(4):1043–50.

44. Dargin JS, Li Q, Jawer G, Xiao X, Mostafavi A. Compound hazards: An examination of how hurricane protective actions could increase transmission risk of COVID-19. International Journal of Disaster Risk Reduction. 2021 Nov;65:102560.

45. Dasgupta S, Bowen VB, Leidner A, Fletcher K, Musial T, Rose C, et al. Association Between Social Vulnerability and a County’s Risk for Becoming a COVID-19 Hotspot — United States, June 1–July 25, 2020. MMWR Morb Mortal Wkly Rep. 2020 Oct 23;69(42):1535–41.

46. De Jesus MCS, Santos VS, Storti-Melo LM, De Souza CDF, Barreto ÍDDC, Paes MVC, et al. Impact of a twelve-year rotavirus vaccine program on acute diarrhea mortality and hospitalization in Brazil: 2006-2018. Expert Review of Vaccines. 2020 Jun 2;19(6):585–93.

47. de Oliveira Mendes JM. Social vulnerability indexes as planning tools: beyond the preparedness paradigm. Journal of Risk Research. 2009 Jan;12(1):43–58.

48. de Souza CDF, Machado MF, Correia DS, do Carmo RF, Cuevas LE, Santos VS. Spatiotemporal clustering, social vulnerability and risk of congenital syphilis in northeast Brazil: an ecological study. Transactions of The Royal Society of Tropical Medicine and Hygiene. 2020 Sep 1;114(9):657–65.

49. Dekker PK, Bhardwaj P, Singh T, Bekeny JC, Kim KG, Steinberg JS, et al. Telemedicine in the Wake of the COVID-19 Pandemic: Increasing Access to Surgical Care. Plastic and Reconstructive Surgery - Global Open. 2021 Jan;9(1):e3228.

50. Delanois RE, Tarazi JM, Wilkie WA, Remily E, Salem HS, Mohamed NS, et al. Social determinants of health in total knee arthroplasty: are social factors associated with increased 30-day post-discharge cost of care and length of stay? The Bone & Joint Journal. 2021 Jun 1;103-B(6 Supple A):113–8.

51. Diaz A, Barmash E, Azap R, Paredes AZ, Hyer JM, Pawlik TM. Association of County-Level Social Vulnerability with Elective Versus Non-elective Colorectal Surgery. J Gastrointest Surg. 2021 Mar;25(3):786–94.

52. Diaz A, Beane JD, Hyer JM, Tsilimigras D, Pawlik TM. Impact of hospital quality on surgical outcomes in patients with high social vulnerability: Association of textbook outcomes and social vulnerability by hospital quality. Surgery. 2022 Jun;171(6):1612–8.

53. Diaz A, Chavarin D, Paredes AZ, Tsilimigras DI, Pawlik TM. Association of Neighborhood Characteristics with Utilization of High-Volume Hospitals Among Patients Undergoing High-Risk Cancer Surgery. Ann Surg Oncol. 2021 Feb;28(2):617–31.

54. Diaz A, Dalmacy D, Hyer JM, Tsilimigras D, Pawlik TM. Intersection of social vulnerability and residential diversity: Postoperative outcomes following resection of lung and colon cancer. Journal of Surgical Oncology. 2021 Oct;124(5):886–93.

55. Diaz A, Hyer JM, Azap R, Tsilimigras D, Pawlik TM. Association of social vulnerability with the use of high-volume and Magnet recognition hospitals for hepatopancreatic cancer surgery. Surgery. 2021 Aug;170(2):571–8.

56. Diaz A, Hyer JM, Barmash E, Azap R, Paredes AZ, Pawlik TM. County-level Social Vulnerability is Associated With Worse Surgical Outcomes Especially Among Minority Patients. Annals of Surgery. 2021 Dec;274(6):881–91.

57. Ebert A, Kerle N, Stein A. Urban social vulnerability assessment with physical proxies and spatial metrics derived from air- and spaceborne imagery and GIS data. Nat Hazards. 2009 Feb;48(2):275–94.

58. Eid M, El-adaway I. Integrating the Social Vulnerability of Host Communities and the Objective Functions of Associated Stakeholders during Disaster Recovery Processes Using Agent-Based Modeling. Journal of Computing in Civil Engineering. 2017;31(4).

59. Estrella JB, Carmichael H, Myers QWO, Lee S, Velopulos CG. Making it Complicated: Does Disparity in Access to Care Lead to More Perforated Appendicitis? Journal of Surgical Research. 2021 Oct;266:405–12.

60. Fergen JT, Bergstrom RD. Social Vulnerability across the Great Lakes Basin: A County-Level Comparative and Spatial Analysis. Sustainability. 2021 Jun 29;13(13):7274.

61. Fernandes Bolina A, Rodrigues RAP, Tavares DM dos S, Haas VJ. Factors associated with the social, individual and programmatic vulnerability of older adults living at home. Rev esc enferm USP. 2019;53:e03429.

62. Franco JV, Garcia MT, Canella DS, Louzada I da R, Bógus CM. Ambiente alimentar de estações de metrô: um estudo no município de São Paulo, Brasil. Ciênc saúde coletiva. 2021 Aug;26(8):3187–98.

63. Freese KE, Vega A, Lawrence JJ, Documet PI. Social Vulnerability Is Associated with Risk of COVID-19 Related Mortality in U.S. Counties with Confirmed Cases. Journal of Health Care for the Poor and Underserved. 2021;32(1):245–57.

64. Freire-Maia FB, Auad SM, Abreu MHNG de, Sardenberg F, Martins MT, Paiva SM, et al. Oral Health-Related Quality of Life and Traumatic Dental Injuries in Young Permanent Incisors in Brazilian Schoolchildren: A Multilevel Approach. leblebicioglu B, editor. PLoS ONE. 2015 Aug 19;10(8):e0135369.

65. Freitas ICM de, Moraes SA de. O efeito da vulnerabilidade social sobre indicadores antropométricos de obesidade: resultados de estudo epidemiológico de base populacional. Rev bras epidemiol. 2016 Jun;19(2):433–50.

66. Fu X, Zhai W. Examining the spatial and temporal relationship between social vulnerability and stay-at-home behaviors in New York City during the COVID-19 pandemic. Sustainable Cities and Society. 2021 Apr;67:102757.

67. Gay JL, Robb SW, Benson KM, White A. Can the Social Vulnerability Index Be Used for More Than Emergency Preparedness? An Examination Using Youth Physical Fitness Data. Journal of Physical Activity and Health. 2016 Feb;13(2):121–30.

68. Ge Y, Dou W, Liu N. Planning Resilient and Sustainable Cities: Identifying and Targeting Social Vulnerability to Climate Change. Sustainability. 2017 Aug 7;9(8):1394.

69. Ge Y, Dou W, Wang X, Chen Y, Zhang Z. Identifying urban–rural differences in social vulnerability to natural hazards: a case study of China. Nat Hazards. 2021 Sep;108(3):2629–51.

70. Georgantopoulos P, Eberth JM, Cai B, Emrich C, Rao G, Bennett CL, et al. Patient- and area-level predictors of prostate cancer among South Carolina veterans: a spatial analysis. Cancer Causes Control. 2020 Mar;31(3):209–20.

71. Gharpure R, Yi SH, Li R, Jacobs Slifka KM, Tippins A, Jaffe A, et al. COVID-19 Vaccine Uptake Among Residents and Staff Members of Assisted Living and Residential Care Communities—Pharmacy Partnership for Long-Term Care Program, December 2020–April 2021. Journal of the American Medical Directors Association. 2021 Oct;22(10):2016-2020.e2.

72. Givens M, Teal EN, Patel V, Manuck TA. Preterm birth among pregnant women living in areas with high social vulnerability. American Journal of Obstetrics & Gynecology MFM. 2021 Sep;3(5):100414.

73. Godfrey EM, Thayer EK, Fiastro AE, Aiken ARA, Gomperts R. Family medicine provision of online medication abortion in three US states during COVID-19. Contraception. 2021 Jul;104(1):54–60.

74. Godin J, Armstrong JJ, Wallace L, Rockwood K, Andrew MK. The impact of frailty and cognitive impairment on quality of life: employment and social context matter. Int Psychogeriatr. 2019 Jun;31(06):789–97.

75. Grunwell JR, Opolka C, Mason C, Fitzpatrick AM. Geospatial Analysis of Social Determinants of Health Identifies Neighborhood Hot Spots Associated With Pediatric Intensive Care Use for Life-Threatening Asthma. The Journal of Allergy and Clinical Immunology: In Practice. 2022 Apr;10(4):981-991.e1.

76. Harrison NE, Ehrman RR, Curtin A, Gorelick D, Hill AB, Brennan E, et al. Factors Associated With Voluntary Refusal of Emergency Medical System Transport for Emergency Care in Detroit During the Early Phase of the COVID-19 Pandemic. JAMA Netw Open. 2021 Aug 20;4(8):e2120728.

77. Hathaway ED. American Indian and Alaska Native People: Social Vulnerability and COVID‐19. The Journal of Rural Health. 2021 Jan;37(1):256–9.

78. Hou J, Lv J, Chen X, Yu S. China’s regional social vulnerability to geological disasters: evaluation and spatial characteristics analysis. Nat Hazards. 2016 Nov;84(S1):97–111.

79. Huang J, Su F, Zhang P. Measuring social vulnerability to natural hazards in Beijing-Tianjin-Hebei Region, China. Chin Geogr Sci. 2015 Aug;25(4):472–85.

80. Hughes MM, Wang A, Grossman MK, Pun E, Whiteman A, Deng L, et al. County-Level COVID-19 Vaccination Coverage and Social Vulnerability — United States, December 14, 2020–March 1, 2021. MMWR Morb Mortal Wkly Rep. 2021 Mar 26;70(12):431–6.

81. Hyer JM, Tsilimigras DI, Diaz A, Dalmacy D, Paro A, Pawlik TM. Patient Social Vulnerability and Hospital Community Racial/Ethnic Integration: Do All Patients Undergoing Pancreatectomy Receive the Same Care Across Hospitals? Annals of Surgery. 2021 Sep;274(3):508–15.

82. Hyer MJ, Tsilimigras DI, Diaz A, Mirdad RS, Azap RA, Cloyd J, et al. High Social Vulnerability and “Textbook Outcomes” after Cancer Operation. Journal of the American College of Surgeons. 2021 Apr;232(4):351–9.

83. Islam N, Lacey B, Shabnam S, Erzurumluoglu AM, Dambha-Miller H, Chowell G, et al. Social inequality and the syndemic of chronic disease and COVID-19: county-level analysis in the USA. J Epidemiol Community Health. 2021 Jun;75(6):496–500.

84. Islam SJ, Nayak A, Hu Y, Mehta A, Dieppa K, Almuwaqqat Z, et al. Temporal trends in the association of social vulnerability and race/ethnicity with county-level COVID-19 incidence and outcomes in the USA: an ecological analysis. BMJ Open. 2021 Jul;11(7):e048086.

85. Javalkar K, Robson VK, Gaffney L, Bohling AM, Arya P, Servattalab S, et al. Socioeconomic and Racial and/or Ethnic Disparities in Multisystem Inflammatory Syndrome. Pediatrics. 2021 May 1;147(5):e2020039933.

86. Jesus ITM de, Orlandi AA dos S, Zazzetta MS. Frailty and social support of the elderly in contexts of social vulnerability. Rev Rene. 2018 Nov 13;19:e32670.

87. Johnson DP, Ravi N, Braneon CV. Spatiotemporal Associations Between Social Vulnerability, Environmental Measurements, and COVID‐19 in the Conterminous United States. Geohealth [Internet]. 2021 Aug [cited 2022 Dec 22];5(8). Available from: https://onlinelibrary.wiley.com/doi/10.1029/2021GH000423

88. Johnson ES, Bell JM, Coker D, Hertz E, LaBarge N, Blake G. A lifeline and social vulnerability analysis of sea level rise impacts on rural coastal communities. Shore and Beach. 2018;86(4):38–44.

89. Jones KK, Anderko L, Davies-Cole J. Neighborhood Environment and Asthma Exacerbation in Washington, DC. Annu Rev Nurs Res. 2019 Dec 23;38(1):53–72.

90. Jorge KO, Oliveira Filho PM, Ferreira EF, Oliveira AC, Vale MP, Zarzar PM. Prevalence and association of dental injuries with socioeconomic conditions and alcohol/drug use in adolescents between 15 and 19 years of age: Dental trauma and associated factors. Dental Traumatology. 2012 Apr;28(2):136–41.

91. Jorge KO, Cota LO, Ferreira EF e, Vale MP do, Kawachi I, Zarzar PM. Tobacco use and friendship networks: a cross-sectional study among Brazilian adolescents. Ciênc saúde coletiva. 2015 May;20(5):1415–24.

92. Jorge KO, Moysés SJ, e Ferreira EF, Ramos-Jorge ML, de Araújo Zarzar PMP. Prevalence and factors associated to dental trauma in infants 1-3 years of age. Dental Traumatology. 2009 Apr;25(2):185–9.

93. Jorge KO, Paiva PCP, Ferreira EF e, Vale MP do, Kawachi I, Zarzar PM. Alcohol intake among adolescent students and association with social capital and socioeconomic status. Ciênc saúde coletiva. 2018 Mar;23(3):741–50.

94. Karaye IM, Horney JA. The Impact of Social Vulnerability on COVID-19 in the U.S.: An Analysis of Spatially Varying Relationships. American Journal of Preventive Medicine. 2020 Sep;59(3):317–25.

95. Karmakar M, Lantz PM, Tipirneni R. Association of Social and Demographic Factors With COVID-19 Incidence and Death Rates in the US. JAMA Netw Open. 2021 Jan 29;4(1):e2036462.

96. Khan SU, Javed Z, Lone AN, Dani SS, Amin Z, Al-Kindi SG, et al. Social Vulnerability and Premature Cardiovascular Mortality Among US Counties, 2014 to 2018. Circulation. 2021 Oct 19;144(16):1272–9.

97. Khazanchi R, Beiter ER, Gondi S, Beckman AL, Bilinski A, Ganguli I. County-Level Association of Social Vulnerability with COVID-19 Cases and Deaths in the USA. J GEN INTERN MED. 2020 Sep;35(9):2784–7.

98. Killian AC, Carter AJ, Reed RD, Shelton BA, Qu H, McLeod MC, et al. Greater community vulnerability is associated with poor living donor navigator program fidelity. Surgery. 2022;172(3):997–1004.

99. Killian AC, Shelton B, MacLennan P, McLeod MC, Carter A, Reed R, et al. Evaluation of Community-Level Vulnerability and Racial Disparities in Living Donor Kidney Transplant. JAMA Surg. 2021 Dec 1;156(12):1120.

100. Lai K, Lane PA. Social Vulnerability Is Associated with Emergency Department Dependency in Pediatric Sickle Cell Disease Patients. Blood. 2019 Nov 13;134(Supplement_1):4680–4680.

101. Lavoie A, Sparks K, Kasperski S, Himes-Cornell A, Hoelting K, Maguire C. Ground-truthing social vulnerability indices of Alaska fishing communities. Coastal Management. 2018 Sep 3;46(5):359–87.

102. LeRose JJ, Merlo C, Duong P, Harden K, Rush R, Artzberger A, et al. The role of the social vulnerability index in personal protective equipment shortages, number of cases, and associated mortality during the coronavirus disease 2019 (COVID-19) pandemic in Michigan skilled nursing facilities. Infect Control Hosp Epidemiol. 2021 Jul;42(7):877–80.

103. Lopes L de MN, Acurcio F de A, Diniz SD, Coelho TL, Andrade EIG. (Un)Equitable distribution of health resources and the judicialization of healthcare: 10 years of experience in Brazil. Int J Equity Health. 2019 Dec;18(1):10.

104. Lotfata A, Ambinakudige S. Natural Disaster and Vulnerability: An Analysis of the 2016 Flooding in Louisiana. Southeastern Geographer. 59(2):130–52.

105. Lottering SJ, Mafongoya P, Lottering RT. Assessing the social vulnerability of small-scale farmer’s to drought in uMsinga, KwaZulu-Natal. International Journal of Disaster Risk Reduction. 2021 Nov;65:102568.

106. Macedo Y, de Almeida L, Camara Maciel A, Troleis A. vulnerabilidade socioambiental em escala de detalhe (Socio-environmental vulnerability in detail scale). GEOSABERES. 2015;6:145–57.

107. Maharani YN, Lee S, Ki SJ. Social vulnerability at a local level around the Merapi volcano. International Journal of Disaster Risk Reduction. 2016 Dec;20:63–77.

108. Martinez EZ, Roza DL da, Caccia-Bava M do CGG, Achcar JA, Dal-Fabbro AL. Gravidez na adolescência e características socioeconômicas dos municípios do Estado de São Paulo, Brasil: análise espacial. Cad Saúde Pública. 2011 May;27(5):855–67.

109. Martinich J, Neumann J, Ludwig L, Jantarasami L. Risks of sea level rise to disadvantaged communities in the United States. Mitig Adapt Strateg Glob Change. 2013 Feb;18(2):169–85.

110. Martins LP, Bittencourt JM, Bendo CB, Vale MP, Paiva SM. Má oclusão e vulnerabilidade social: estudo representativo de adolescentes de Belo Horizonte, Brasil. Ciênc saúde coletiva. 2019 Feb;24(2):393–400.

111. Martins MT, Sardenberg F, Vale MP, Paiva SM, Pordeus IA. Dental caries and social factors: impact on quality of life in Brazilian children. Braz oral res [Internet]. 2015 [cited 2022 Dec 23];29(1). Available from: http://www.scielo.br/scielo.php?script=sci_arttext&pid=S1806-83242015000100310&lng=en&tlng=en

112. Martins M, Sardenberg F, Abreu M, Vale M, Paiva S, Pordeus I. Factors associated with dental caries in Brazilian children: a multilevel approach. Community Dent Oral Epidemiol. 2014 Aug;42(4):289–99.

113. Martins-Filho PR, Quintans-Júnior LJ, de Souza Araújo AA, Sposato KB, Souza Tavares CS, Gurgel RQ, et al. Socio-economic inequalities and COVID-19 incidence and mortality in Brazilian children: a nationwide register-based study. Public Health. 2021 Jan;190:4–6.

114. Martins-Oliveira JG, Jorge KO, Ferreira RC, Ferreira EF e, Vale MP, Zarzar PM. Risk of alcohol dependence: prevalence, related problems and socioeconomic factors. Ciênc saúde coletiva. 2016 Jan;21(1):17–26.

115. Mavhura E, Manyangadze T. A comprehensive spatial analysis of social vulnerability to natural hazards in Zimbabwe: Driving factors and policy implications. International Journal of Disaster Risk Reduction. 2021 Apr;56:102139.

116. Mavhura E, Manyena B. Spatial quantification of community resilience in contexts where quantitative data are scarce: The case of Muzarabani district in Zimbabwe. Geo: Geography and Environment [Internet]. 2018 Jul [cited 2022 Dec 23];5(2). Available from: https://onlinelibrary.wiley.com/doi/10.1002/geo2.65

117. McAlarnen LA, Tsaih SW, Aliani R, Simske NM, Hopp EE. Virtual visits among gynecologic oncology patients during the COVID-19 pandemic are accessible across the social vulnerability spectrum. Gynecologic Oncology. 2021 Jul;162(1):4–11.

118. Mengal A, Goda K, Ashraf M, Murtaza G. Social vulnerability to seismic-tsunami hazards in district Gwadar, Balochistan, Pakistan. Nat Hazards. 2021 Aug;108(1):1159–81.

119. Mock J, Meyer C, Mau LW, Nguyen C, Arora P, Heron C, et al. Barriers to Access to Hematopoietic Cell Transplantation among Patients with Acute Myeloid Leukemia in Virginia. Transplantation and Cellular Therapy. 2021 Oct;27(10):869.e1-869.e9.

120. Morgan ME, Horst MA, Vernon TM, Fallat ME, Rogers AT, Bradburn EH, et al. An analysis of pediatric social vulnerability in the Pennsylvania trauma system. Journal of Pediatric Surgery. 2020 Dec;55(12):2746–51.

121. Muyambo F, Jordaan AJ, Bahta YT. Assessing social vulnerability to drought in South Africa: Policy implication for drought risk reduction. Jàmbá [Internet]. 2017 Jan 31 [cited 2022 Dec 22];9(1). Available from: https://jamba.org.za/index.php/jamba/article/view/326

122. Nafeh AMB, Beldjoudi H, Yelles AK, Monteiro R. Development of a seismic social vulnerability model for northern Algeria. International Journal of Disaster Risk Reduction. 2020 Nov;50:101821.

123. Nakamura PM, Teixeira IP, Hino AAF, Kerr J, Kokubun E. Association between private and public places and practice of physical activity in adults. Rev Bras Cineantropom Desempenho Hum. 2016 Jul 14;18(3):297.

124. Nascimento LFC. CONGENITAL SYPHILIS IN THE PARAÍBA VALLEY USING A SPATIAL APPROACH. Rev paul pediatr. 2020;38:e2018395.

125. Nayak A, Islam SJ, Mehta A, Ko YA, Patel SA, Goyal A, et al. Impact of Social Vulnerability on COVID-19 Incidence and Outcomes in the United States [Internet]. Public and Global Health; 2020 Apr [cited 2022 Dec 22]. Available from: http://medrxiv.org/lookup/doi/10.1101/2020.04.10.20060962

126. Neelon B, Mutiso F, Mueller NT, Pearce JL, Benjamin-Neelon SE. Spatial and temporal trends in social vulnerability and COVID-19 incidence and death rates in the United States. Goldfarb SS, editor. PLoS ONE. 2021 Mar 24;16(3):e0248702.

127. Oates GR, Juarez LD, Horswell R, Chu S, Miele L, Fouad MN, et al. The Association Between Neighborhood Social Vulnerability and COVID-19 Testing, Positivity, and Incidence in Alabama and Louisiana. J Community Health. 2021 Dec;46(6):1115–23.

128. Ouvrard C, Avila-Funes JA, Dartigues JF, Amieva H, Tabue-Teguo M. The Social Vulnerability Index: Assessing Replicability in Predicting Mortality Over 27 Years: Letter to the Editor. J Am Geriatr Soc. 2019 Jun;67(6):1305–6.

129. Papageorge MV, Woods AP, de Geus SWL, Ng SC, Paasche-Orlow MK, Segev D, et al. Beyond insurance status: the impact of Medicaid expansion on the diagnosis of Hepatocellular Carcinoma. HPB. 2022 Aug;24(8):1271–9.

130. Paro A, Hyer JM, Diaz A, Tsilimigras DI, Pawlik TM. Profiles in social vulnerability: The association of social determinants of health with postoperative surgical outcomes. Surgery. 2021 Dec;170(6):1777–84.

131. Pascom ARP, Meireles MV, Benzaken AS. Sociodemographic determinants of attrition in the HIV continuum of care in Brazil, in 2016. Medicine. 2018 May;97(1S):S69–74.

132. Phelos HM, Deeb AP, Brown JB. Can social vulnerability indices predict county trauma fatality rates? J Trauma Acute Care Surg. 2021 Aug;91(2):399–405.

133. Phelos HM, Deeb AP, Brown JB. Can social vulnerability indices predict county trauma fatality rates? J Trauma Acute Care Surg. 2021 Aug;91(2):399–405.

134. Puvvula J, Bartelt-Hunt SL, Ouattara BS, Kolok AS, Bell JE, Rogan EG. Association between Aqueous Atrazine and Pediatric Cancer in Nebraska. Water. 2021 Oct 2;13(19):2727.

135. Ratnapradip D, McDaniel JT, Barger A. Social vulnerability and Lyme disease incidence: a regional analysis of the United States, 2000-2014. Epidemiology, Biostatistics and Public Health. 2017;14(2).

136. Regmi MR, Tandan N, Parajuli P, Bhattarai M, Maini R, Kulkarni A, et al. Social Vulnerability Indices as a Risk Factor for Heart Failure Readmissions. Clin Med Res. 2021 Sep;19(3):116–22.

137. Ribeiro CJN, dos Santos AD, Lima SVMA, da Silva ER, Ribeiro BVS, Duque AM, et al. Space-time risk cluster of visceral leishmaniasis in Brazilian endemic region with high social vulnerability: An ecological time series study. Ajjampur SS, editor. PLoS Negl Trop Dis. 2021 Jan 19;15(1):e0009006.

138. Ribeiro KB, Andrade F, Andrade D, Pedro S, Yoshimura A, Geronymo B, et al. Do socioeconomic status and comorbidity affect survival in women with breast cancer living in the city of São Paulo, Brazil? JCO. 2017 May 20;35(15):e18150–e18150.

139. Rickless DS, Wilt GE, Sharpe JD, Molinari N, Stephens W, LeBlanc TT. Social Vulnerability and Access of Local Medical Care During Hurricane Harvey: A Spatial Analysis. Disaster med public health prep. 2021 Mar 15;1–9.

140. Rifat SAA, Senkbeil JC, Liu W. Assessing Influential Factors on Inland Property Damage from Gulf of Mexico Tropical Cyclones in the United States. IJGI. 2021 May 4;10(5):295.

141. Saia SM, Suttles KM, Cutts BB, Emanuel RE, Martin KL, Wear DN, et al. Applying Climate Change Risk Management Tools to Integrate Streamflow Projections and Social Vulnerability. Ecosystems. 2020 Jan;23(1):67–83.

142. Schmidtlein MC, Shafer JM, Berry M, Cutter SL. Modeled earthquake losses and social vulnerability in Charleston, South Carolina. Applied Geography. 2011 Jan;31(1):269–81.

143. Sedrez M, Martino J de. Amor SP Understanding socio-spatial emotions of urban poor in São Paulo. In Porto, Portugal: Editora Blucher; 2019 [cited 2022 Dec 23]. p. 829–36. Available from: http://www.proceedings.blucher.com.br/article-details/34332

144. Serra-Negra JM, Paiva SM, Seabra AP, Dorella C, Lemos BF, Pordeus IA. Prevalence of sleep bruxism in a group of Brazilian schoolchildren. Eur Arch Paediatr Dent. 2010 Aug;11(4):192–5.

145. Serra-Negra JM, Ramos-Jorge ML, Flores-Mendoza CE, Paiva SM, Pordeus IA. Influence of psychosocial factors on the development of sleep bruxism among children. International Journal of Paediatric Dentistry. 2009 Sep;19(5):309–17.

146. Serra-Negra JM, Paiva SM, Abreu MH, Flores-Mendoza CE, Pordeus IA. Relationship between Tasks Performed, Personality Traits, and Sleep Bruxism in Brazilian School Children - A Population-Based Cross-Sectional Study. Xia Y, editor. PLoS ONE. 2013 Nov 14;8(11):e80075.

147. Serra-Negra J, Paiva SM, Oliveira M, Ferreira E, Freire-Maia F, Pordeus I. Self-Reported Dental Fear among Dental Students and Their Patients. IJERPH. 2011 Dec 29;9(1):44–54.

148. Sharareh N, Hess R, White S, Dunn A, Singer PM, Cochran J. A vulnerability assessment for the HCV infections associated with injection drug use. Preventive Medicine. 2020 May;134:106040.

149. Silva-Oliveira F, Jorge KO, Ferreira EF e, Vale MP, Kawachi I, Zarzar PM. The prevalence of inhalant use and associated factors among adolescents in Belo Horizonte, Brazil. Ciênc saúde coletiva. 2014 Mar;19(3):881–90.

150. Souza CDF, Rocha VS, Santos NF, Leal TC, Paiva JPS, Oliveira CCC, et al. Spatial clustering, social vulnerability and risk of leprosy in an endemic area in Northeast Brazil: an ecological study. J Eur Acad Dermatol Venereol. 2019 Aug;33(8):1581–90.

151. Souza CDF de, Oliveira DJ de, Silva LF da, Santos CD dos, Pereira MC, Paiva JPS de, et al. Tendência da Mortalidade por Doenças Cerebrovasculares no Brasil (1996-2015) e Associação com Desenvolvimento Humano e Vulnerabilidade Social (Cerebrovascular Disease Mortality Trend in Brazil (1996 To 2015) and Association with Human Development Index and Social Vulnerability). Arquivos Brasileiros de Cardiologia. 2021 Jan 27;116(1):89–99.

152. Souza KOC de, Fracolli LA, Ribeiro CJN, Menezes AF de, Silva GM, Santos AD dos. Quality of basic health care and social vulnerability: a spatial analysis. Rev esc enferm USP. 2021;55:e20200407.

153. Steinkamp L, Deuel D, Lucre M, Zavala P. The Interplay of Diversity, Equity, and Inclusion in Addressing Health Inequities. WMJ. 2021 Mar;120(S1):S54–8.

154. Strully KW, Yang TC. County Social Vulnerability and Influenza Vaccine Rates: National and Local Estimates for Medicare Recipients. American Journal of Preventive Medicine. 2022 Jan;62(1):e1–9.

155. Tellman B, Schank C, Schwarz B, Howe PD, de Sherbinin A. Using Disaster Outcomes to Validate Components of Social Vulnerability to Floods: Flood Deaths and Property Damage across the USA. Sustainability. 2020 Jul 27;12(15):6006.

156. Thakore N, Khazanchi R, Orav EJ, Ganguli I. Association of Social Vulnerability, COVID-19 vaccine site density, and vaccination rates in the United States. Healthcare. 2021 Dec;9(4):100583.

157. Troppy S, Wilt GE, Whiteman A, Hallisey E, Crockett M, Sharpe JD, et al. Geographic Associations Between Social Factors and SARS-CoV-2 Testing Early in the COVID-19 Pandemic, February–June 2020, Massachusetts. Public Health Rep. 2021 Nov;136(6):765–73.

158. Tummalapalli SL, Silberzweig J, Cukor D, Lin JT, Barbar T, Liu Y, et al. Racial and Neighborhood-Level Disparities in COVID-19 Incidence among Patients on Hemodialysis in New York City. JASN. 2021 Aug;32(8):2048–56.

159. Turek-Hankins LL, Hino M, Mach KJ. Risk screening methods for extreme heat: Implications for equity-oriented adaptation. McFadden J, editor. PLoS ONE. 2020 Nov 4;15(11):e0240841.

160. Upchurch DM, Wong MS, Yuan AH, Haderlein TP, McClendon J, Christy A, et al. COVID-19 Infection in the Veterans Health Administration: Gender-specific Racial and Ethnic Differences. Women’s Health Issues. 2022 Jan;32(1):41–50.

161. Vickers S. High Social Vulnerability and" Textbook Outcomes" after Cancer Operation. Journal of the American College of Surgeons. 2021;232(4).

162. Viegas CM, Scarpelli AC, Carvalho AC, Ferreira FM, Pordeus IA, Paiva SM. Predisposing factors for traumatic dental injuries in Brazilian preschool children. Eur J Paediatr Dent. 2010 Jun;11(2):59–65.

163. Vo A, Bhaskar R, Chi TY, Faddoul G. Identifying socially vulnerable regions with persistent low accessibility to emergency care through a spatial decision framework. Journal of Decision Systems. 2020 Oct 1;29(4):201–22.

164. Wanderley FSO, Montarroyos U, Bonfim C, Cunha-Correia C. Effectiveness of mass treatment of Schistosoma mansoni infection in socially vulnerable areas of a state in northeastern Brazil, 2011–2014. Arch Public Health. 2021 Dec;79(1):30.

165. Wang C, Li Z, Clay Mathews M, Praharaj S, Karna B, Solís P. The spatial association of social vulnerability with COVID-19 prevalence in the contiguous United States. International Journal of Environmental Health Research. 2022 May 4;32(5):1147–54.

166. Wang H, Xu R, Qu S, Schwartz M, Adams A, Chen X. Health inequities in COVID-19 vaccination among the elderly: Case of Connecticut. Journal of Infection and Public Health. 2021 Oct;14(10):1563–5.

167. Wigtil G, Hammer RB, Kline JD, Mockrin MH, Stewart SI, Roper D, et al. Places where wildfire potential and social vulnerability coincide in the coterminous United States. Int J Wildland Fire. 2016;25(8):896.

168. Wood NJ, Burton CG, Cutter SL. Community variations in social vulnerability to Cascadia-related tsunamis in the U.S. Pacific Northwest. Nat Hazards. 2010 Feb;52(2):369–89.

169. Yee CW, Cunningham SD, Ickovics JR. Application of the Social Vulnerability Index for Identifying Teen Pregnancy Intervention Need in the United States. Matern Child Health J. 2019 Nov;23(11):1516–24.

170. Yee V, Moten A. Critical Management of COVID-19: Social Determinants of Health and Public Health Preparedness. Critical Care Medicine. 2021;49(1).

171. Zachrison KS, Yan Z, Sequist T, Licurse A, Tan-McGrory A, Erskine A, et al. Patient characteristics associated with the successful transition to virtual care: Lessons learned from the first million patients. J Telemed Telecare. 2021 Jun 13;1357633X2110155.

172. Zarzar PM, Jorge KO, Oksanen T, Vale MP, Ferreira EF, Kawachi I. Association between binge drinking, type of friends and gender: A cross-sectional study among Brazilian adolescents. BMC Public Health. 2012 Dec;12(1):257.

173. Zhu J, Lu Y, Ren F, McBride JL, Ye L. Typhoon disaster risk zoning for China’s coastal area. Front Earth Sci. 2022 Jun;16(2):291–303.

174. Zottarelli LK, Sharif HO, Xu X, Sunil TS. Effects of social vulnerability and heat index on emergency medical service incidents in San Antonio, Texas, in 2018. J Epidemiol Community Health. 2020 Oct 14;jech-2019-213256.
