# Supplementary material for: Social vulnerability indices: a scoping review
Source: BMC Public Health. 2023 Jun 28;23:1253. doi: 10.1186/s12889-023-16097-6 (PMC10304642; doi:10.1186/s12889-023-16097-6)
Supplement: Supplementary file 3 — Additional file 3. Characteristics, composition and outcomes of original social vulnerability indices (in white), and characteristics and outcomes replicated social vulnerability indices (in grey). [file 12889_2023_16097_MOESM3_ESM.docx]

## Additional File 3. Characteristics, composition and outcomes of original social vulnerability indices (in white), and characteristics and outcomes replicated social vulnerability indices (in grey)

| **Author, Year**  **(Country)** | **Objective** | **Field of Study** | **Items From** | **Number of Items** | **Weighted Items?** | **Type of Scale Used in Analysis** | **Type of Scale Presented in Results** | **Outcome (if predictive)** | **Direction (if predictive)** |
| --- | --- | --- | --- | --- | --- | --- | --- | --- | --- |
| Abeliansky, 2021  (USA) | To gain insights on the impact of social vulnerability on physiological aging at the individual level and at the cohort level. | Health/ Medicine | representative survey | 49 | No | numeric | numeric | Frailty | Positive |
| Adger. 2005  (Multiple in Africa) | To outline the nature of uncertainty for the major elements of adaptive capacity and illustrate these issues with the example of a social vulnerability index for countries in Africa. | Climate/ Environment/ Disaster | mixed | 9 | Yes | numeric | numeric | NA | NA |
| Aksha, 2019  (Nepal) | To investigate social vulnerability in Nepal by adapting Social Vulnerability Index methods to the Nepali context. | Climate/ Environment/ Disaster | census or geographical data | 7 | No | numeric | ordinal | NA | NA |
| Andrew, 2014  (Canada) | To investigate many social factors in relation to one another and to survival among older adults using a social ecology perspective to measure social vulnerability among older adults. | Health/ Medicine | representative survey | 28 | No | numeric | numeric | Mortality | Positive |
| Andrew, 2008  (Canada) | To operationalize social vulnerability according to a deficit accumulation approach, to compare social vulnerability and frailty, and to study social vulnerability in relation to mortality. | Health/ Medicine | representative survey | 40 (1^st^ SVI)  23 (2^nd^ SVI) | No | numeric | numeric | Frailty, Mortality | Positive |
| Ouvrard, 2019  (France) | To assess the replicability of the SVI by confirming its association with mortality in the context of a French population-based study. | Health/ Medicine | - | - | - | - | - | Mortality | Positive |
| Andrew, 2010  (Canada) | To investigate whether social vulnerability is associated with cognitive decline in community-dwelling older adults aged 70 years and older. | Health/ Medicine | - | - | - | - | - | Cognitive decline | Positive |
| Armaș, 2013  (Romania) | To assess two multi-criteria methods for aggregating complex indicators: the social vulnerability index (SoVI model) and the spatial multi-criteria social vulnerability index (SEVI model). | Climate/ Environment/ Disaster | mixed | 18 | Yes | numeric | numeric | NA | NA |
| Armaş, 2016  (Romania) | To explore areas at risk and their spatial association by applying a social vulnerability index at the 2011 census. | Climate/ Environment/ Disaster | - | - | - | - | - | NA | NA |
| Armstrong, 2015  (USA) | To evaluate mortality risk in relation to social vulnerability across levels of frailty among a cohort of older Japanese-American men. | Health/ Medicine | other | 19 | No | numeric | numeric | Mortality | Positive |
| Armstrong, 2015  (USA) | To look at changes in cognition in relation to frailty, social vulnerability, and protective factors in the Honolulu-Asia Aging Study (HAAS). | Health/ Medicine | - | - | - | - | - | Changes in cognition | Positive |
| Aroca-Jimenez, 2017  (Spain) | To describe a methodological approach towards constructing the ISVI in urban areas prone to flash flooding in Castilla y León. | Climate/ Environment/ Disaster | mixed | 38 | No | numeric | ordinal | NA | NA |
| Aroca-Jiménez, 2020  (Spain) | To describe the construction of an Integrated Socio-Economic Vulnerability Index (ISEVI) at the regional scale. | Climate/ Environment/ Disaster | mixed | 60 | No | numeric | ordinal | NA | NA |
| Badmos, 2018  (Ghana) | To determine the social vulnerability index (SoVI) of households to climate change impacts for three identified locations in the Vea catchment, semi-arid Ghana. | Climate/ Environment/ Disaster | representative survey | 11 | Yes | numeric | numeric | NA | NA |
| Ballesteros, 2021  (Multiple in Africa) | To create an index of vulnerability to coastal change, integrating indices of social vulnerability and exposure to coastal hazards for East Africa to identify ‘areas of priority concern’ for risk reduction. | Climate/ Environment/ Disaster | census or geographical data | 8 | No | numeric | ordinal | NA | NA |
| Berrouet, 2019  (Colombia) | To present the conceptual framework for the vulnerability assessment of the social system on which the methodological proposal and index are based. To propose an index for evaluating social vulnerability to changes in the provision of ecosystem services. | Climate/ Environment/ Disaster | census or geographical data | 4 | No | numeric | numeric | NA | NA |
| Bjarnadottir, 2011  (USA) | To presents the development of the Coastal Community Social Vulnerability Index (CCSVI) in order to quantify the social vulnerability of hurricane-prone areas under various scenarios of climate change. | Climate/ Environment/ Disaster | census or geographical data | 17 | Yes | numeric | numeric | NA | NA |
| Brazilian Social Vulnerability Atlas, 2015  (Brazil) | To describe the Índice de Vulnerabilidade Social (IVS). | Mixed | census or geographical data | 16 | Yes | numeric | numeric | NA | NA |
| Andrade, 2022  (Brazil) | To assess the spatiotemporal distribution of mortality and lethality rates of COVID-19 in a region of high social vulnerability in Brazil. | Health/ Medicine | - | - | - | - | - | Covid-19 mortality | Positive |
| Baggio, 2021  (Brazil) | To analyse clinical-epidemiological variables, incidence rate, mortality rate, case fatality rate and the social indicators municipal human development index and social vulnerability index. | Health/ Medicine | - | - | - | - | - | Covid-19 incidence rate, mortality rate, case fatality rate | Positive |
| Brito, 2020  (Brazil) | To assess the dental caries experience and associated factors among 12-year-old children in the state of São Paulo, Brazil. | Health/ Medicine | - | - | - | - | - | Dentition | No association |
| Nascimento, 2020  (Brazil) | To compare spatial patterns of congenital syphilis (CS) with those of socioeconomic and medical variables in Paraíba Valley, São Paulo, between 2012 and 2016. | Health/ Medicine | - | - | - | - | - | NA | NA |
| Curi, 2021  (Brazil) | To analyze Brazilian coastal municipalities, based on two indexes: The Social Vulnerability Index and the Municipal Human Development Index. | Climate/ Environment/ Disaster | - | - | - | - | - | NA | NA |
| de Souza, 2021  (Brazil) | To analyze the mortality trend of cerebrovascular disease in Brazil and its association with Human Development Index and the Social Vulnerability Index. | Health/ Medicine | - | - | - | - | - | Cerebrovascular disease mortality | Negative |
| de Souza, 2019  (Brazil) | To examine the spatial and space-time distribution of leprosy and the influence of social vulnerability on the occurrence of the disease in an endemic area of Northeast Brazil. | Health/ Medicine | - | - | - | - | - | Leprosy transmission | Positive |
| de Souza, 2020  (Brazil) | To investigate the spatial distribution of congenital syphilis and its association to social vulnerability indexes in northeast Brazil. | Health/ Medicine | - | - | - | - | - | Congential syphillis | Positive |
| Lopes, 2019  (Brazil) | To assess what socioeconomic factors are associated to municipalities that had larger numbers of beneficiaries from lawsuits in health in the state of Minas Gerais, Brazil, from 1999 to 2009. | Health/ Medicine | - | - | - | - | - | NA | NA |
| Martins-Filho, 2021  (Brazil) | To estimate the incidence and mortality rates COVID-19 in Brazilian children and to analyze its relationship with socio-economic inequalities in a state-level analysis. | Health/ Medicine | - | - | - | - | - | COVID-19 incidence & mortality | Positive |
| Pascom, 2018  (Brazil) | To identify sociodemographic factors associated with attrition in the 3 steps of the HIV continuum of care related to the 90-90-90 targets – access to diagnosis, treatment initiation, and virologic suppression, in Brazilian adults in 2016. | Health/ Medicine | - | - | - | - | - | Access to HIV services | Positive |
| Ribeiro, 2021  (Brazil) | To analyse the spatiotemporal dynamics of visceral leishmaniasis cases to identify the temporal trends and high-risk areas for VL transmission, as well as the association of the disease with social vulnerability in Brazilian Northeast. | Health/ Medicine | - | - | - | - | - | Visceral leishmaniasis | Positive |
| Ribeiro, 2017  (Brazil) | To assess the effect of comorbidity and socioeconomic status on breast cancer survival in a large metropolitan area in Brazil with universal health care. | Health/ Medicine | - | - | - | - | - | Breast cancer survival | No association |
| Souza, 2021  (Brazil) | To analyze the association between quality of basic health care and social vulnerability in municipalities of the Brazilian northeast. | Health/ Medicine | - | - | - | - | - | Quality of basic health care | Positive |
| Wanderley, 2021  (Brazil) | To assess the effectiveness of mass treatment of Schistosoma mansoni infection in socially vulnerable endemic areas in northeastern Brazil. | Health/ Medicine | - | - | - | - | - | Schistosoma mansoni infection | Positive |
| Bronfman, 2021  (Chile) | To explore the spatial and temporal variations in social vulnerability in Chile for the last two decades. | Climate/ Environment/ Disaster | census or geographical data | 30 | Yes | numeric | ordinal | NA | NA |
| Bunt, 2017  (The Netherlands) | To adapt the Social Vulnerability Index to the Dutch language and culture for those purposes. | Health/ Medicine | clinical data | 32 | No | numeric | numeric | NA | NA |
| Burton, 2008  (USA) | To examine the spatial variability in the social vulnerability of residents to potential levee failures in the Sacramento Delta region. | Climate/ Environment/ Disaster | census or geographical data | 36 | No | numeric | numeric | NA | NA |
| Cerami, 2021  (Italy) | We assessed frailty and social vulnerability indices in 1,258 Italian residents during the first lockdown phase *via* an on-line survey. We compared indices taking into account age categories and gender. | Health/ Medicine | representative survey | 30 | No | numeric | numeric | Disease risk | Positive |
| Chakraborty, 2005  (USA) | To examine spatial variability in evacuation assistance needs as related to the hurricane hazard. | Climate/ Environment/ Disaster | census or geographical data | 10 | No | numeric | ordinal | NA | NA |
| Chau, 2014  (USA & Hong Kong) | To modify and use an SVI specifically designed to assess the vulnerability of older populations to emergencies and disasters across seven domains. | Health/ Medicine | administrative data | 7 | No | ordinal | ordinal | NA | NA |
| Chen, 2013  (China) | To replicate and test the applicability of the place-based Social Vulnerability Index developed for the United States in a Chinese cultural context. | Climate/ Environment/ Disaster | census or geographical data | 29 | No | numeric | ordinal | NA | NA |
| Zhu, 2021  (China) | In this study, typhoon disaster risk zoning is conducted for China’s coastal area, based on data at the county level. | Climate/ Environment/ Disaster | - | - | - | - | - | NA | NA |
| Chen, 2021  (China) | To combine flood hazard and social vulnerability index to capture the potential risk of flood. | Climate/ Environment/ Disaster | census or geographical data | 21 | No | numeric | ordinal | NA | NA |
| Cumberbatch, 2020  (Barbados) | A Social Vulnerability Index was developed for Barbados to identify geospatial variations in social vulnerability. | Climate/ Environment/ Disaster | census or geographical data | 8 | No | numeric | ordinal | NA | NA |
| Cutler, 2018  (USA) | To use multilevel statistical modeling to investigate individual- and geographic-level (e.g., census tract level and regional) social, economic, and biophysical influences on public perceptions of the adverse health impacts associated with heat waves. | Climate/ Environment/ Disaster | representative survey | 8 | No | numeric | numeric | Health risks of heat waves | Positive |
| Cutter, 2003  (USA) | County-level socioeconomic and demographic data were used to construct an index of social vulnerability to environmental hazards, called the Social Vulnerability Index (SoVI) for the United States | Climate/ Environment/ Disaster | census or geographical data | 11 | No | numeric | ordinal | NA | NA |
| Anderson, 2019  (USA) | Here, the Social Vulnerability Index (SoVI®) and the vulnerability component of the Global Delta Risk Index (GDRI) are applied at census tract level in the Mississippi Delta and visually and quantitatively compared. | Climate/ Environment/ Disaster | - | - | - | - | - | NA | NA |
| Barboza, 2019  (USA) | To quantify the spatiotemporal risk of child abuse and neglect in Los Angeles at the census tract level over a recent 4-year period, identify areas of increased risk, and evaluate the role of structural disadvantage in child maltreatment referrals. | Health/ Medicine | - | - | - | - | - | Child abuse and neglect | Positive |
| Burton, 2010  (USA) | To examine to what extent can a quantified measure of social vulnerability be incorporated into numerical hurricane impact modeling to improve loss prediction. | Climate/ Environment/ Disaster | - | - | - | - | - | NA | NA |
| Cutter, 2013  (USA) | This paper proposes a methodology for incorporating a known measure of social vulnerability, the Social Vulnerability Index (SoVI), into USACE civil works planning. | Climate/ Environment/ Disaster | - | - | - | - | - | NA | NA |
| de Oliveira Mendes, 2009  (Portugal) | The main objective is to expand the analysis of social vulnerability to technological and social risks, and to incorporate the Social Vulnerability Index as a planning tool. | Mixed | - | - | - | - | - | NA | NA |
| Ebert, 2009  (Honduras) | This article deals with the assessment of social vulnerability in conjunction with a new method based on contextual analysis of image and GIS data. | Climate/ Environment/ Disaster | - | - | - | - | - | NA | NA |
| Eid, 2017  (USA) | To present a decision-making framework for disaster recovery that uses a bottom-up approach to capture the needs of the impacted residents and decreases the social vulnerability of host communities. | Climate/ Environment/ Disaster | - | - | - | - | - | NA | NA |
| Ge, 2021  (China) | To conduct an assessment of the social vulnerability index by applying the projection pursuit cluster model. | Climate/ Environment/ Disaster | \| - \| - \| - \| - \| - \| \| --- \| --- \| --- \| --- \| --- \| | - | - | - | - | NA | NA |
| Georgantopoulos, 2020  (USA) | To use the Veterans Administration Medical System, which provides a unique means for studying prostate cancer epidemiology among diverse individuals with ostensibly equal access to healthcare. | Health/ Medicine | - | - | - | - | - | Prostate cancer risk | Positive |
| Hou, 2016  (China) | To calculate the social vulnerability index of geological disasters in China with the super-efficiency DEA (data envelopment analysis) model, carry out global and local autocorrelation tests for social vulnerability to geological disasters in each province in China and identify the characteristics of its spatial distribution pattern. | Climate/ Environment/ Disaster | - | - | - | - | - | Exposure degree and reaction and recovery ability | Positive |
| Huang, 2015  (China) | This article presents an initial study of the social vulnerability of the Beijing-Tianjin-Hebei Region in China. The goal is to replicate and test the applicability of the United States Social Vulnerability Index (SoVI) method. | Climate/ Environment/ Disaster | - | - | - | - | - | NA | NA |
| Lavoie, 2018  (USA) | This paper presents a ground-truthing effort to validate quantitative indices that characterize the well-being of Alaska fishing communities. | Climate/ Environment/ Disaster | - | - | - | - | - | NA | NA |
| Lottering, 2021  (South Africa) | The aim of this article was to assess and identify social vulnerability amongst small-scale farmers and rural communities in the uMsinga community in the KwaZulu Natal province of South Africa using an adapted social vulnerability index (SoVI). | Climate/ Environment/ Disaster | - | - | - | - | - | NA | NA |
| Maharani, 2016  (Indonesia) | This study proposes a computational method for data analysis in terms of the number of social vulnerability variables and samples of the case study in the Merapi proximal villages. | Climate/ Environment/ Disaster | - | - | - | - | - | NA | NA |
| Martinich, 2013  (USA) | To identify geographic areas in the contiguous United States that may be more likely to experience disproportionate impacts of sea level rise and to determine if and where socially vulnerable populations would bear disproportionate costs of adaptation. | Climate/ Environment/ Disaster | - | - | - | - | - | protected vs abonnement in relation inundation from. sea level rise | Positive |
| Mengal, 2021  (Pakistan) | This study presents the first social vulnerability index study for District Gwadar in relation to imminent earthquake-tsunami hazards and shows how SoVI concepts and indicators are adopted. | Climate/ Environment/ Disaster | - | - | - | - | - | NA | NA |
| Muyambo, 2017  (South Africa) | To assess and identify social vulnerability of communal farmers to drought in the O.R. Tambo district in the Eastern Cape province of South Africa using a survey data and social vulnerability index. | Climate/ Environment/ Disaster | - | - | - | - | - | NA | NA |
| Nafeh, 2020  (Algeria) | This paper investigates the social vulnerability and resilience level to natural hazards, with a specific focus on seismic risk, in the province of Blida, an important cultural and economic region in Northern Algeria. | Climate/ Environment/ Disaster | - | - | - | - | - | NA | NA |
| Phelos, 2021  (USA) | Our objective was to determine if these indices correlate with injury fatality rates in the US. | Climate/ Environment/ Disaster | - | - | - | - | - | Injury fatality rates (overall, firearm, and motor vehicle collision deaths) | Positive |
| Ratnapradipa, 2017  (USA) | The purpose of this study was to determine if a relationship existed between the SoVI and Lyme Disease incidence at the national level and regional division level in the United States. | Health/ Medicine | - | - | - | - | - | Lyme disease incidence | Mixed |
| Schmidtlein, 2011  (USA) | This paper examines the spatial linkage between social vulnerability and estimated earthquake losses for differing levels of event magnitude. | Climate/ Environment/ Disaster | - | - | - | - | - | Earthquake losses | Positive |
| Tellman, 2020  (USA) | This paper validates social vulnerability indicators using two flood outcomes: death and damage. | Climate/ Environment/ Disaster | - | - | - | - | - | Flood damage and death | Positive |
| Wigtil, 2016  (USA) | We evaluated place vulnerability to wildfire hazards in the coterminous US by developing a social vulnerability index. | Climate/ Environment/ Disaster | - | - | - | - | - | Wildfire potential | Mixed |
| Wood, 2010  (USA) | In order to assess social vulnerability to Cascadia tsunamis, we adjust a social vulnerability index to operate at the census-block level of geography and focus on community-level comparisons along the Oregon coast. | Climate/ Environment/ Disaster | - | - | - | - | - | NA | NA |
| Denver, 1988  (USA) | The purpose of this article is to identify areas of social vulnerability so that a more positive, long-term approach can be established to create justice in health care planning. | Health/ Medicine | census or geographical data | 13 | No | numeric | categorical | NA | NA |
| de Loyola Hummell, 2016  (Brazil) | This article provides a social vulnerability index (SoVI®) replication study for Brazil and shows how SoVI® concepts and indicators were adapted to the country. | Climate/ Environment/ Disaster | census or geographical data | 10 | No | numeric | numeric | NA | NA |
| Alem, 2021  (Brazil) | This paper addresses the design of a humanitarian supply chain to integrate logistics activities in an effective and efficient decision support system to cope with multiple disaster events over a dynamic time horizon. | Climate/ Environment/ Disaster | - | - | - | - | - | NA | NA |
| di Girasole, 2017  (Dominican Republic) | This article presents a methodology for the analysis of social vulnerability, defined and experimented in the context of the international cooperation project. | Climate/ Environment/ Disaster | mixed | 30 | Yes | numeric | ordinal | NA | NA |
| Dintwa, 2019  (Botswana) | This study applies the household social vulnerability methodology to measure social vulnerability to natural hazards in Botswana. | Climate/ Environment/ Disaster | census or geographical data | 11 | Yes | numeric | ordinal | NA | NA |
| Dintwa, 2019  (Botswana) | The study examined the applicability of the Place Vulnerability Model in Botswana, as well as to analyse underlying factors contributing to social vulnerability to natural hazards. | Climate/ Environment/ Disaster | census or geographical data | 11 | Yes | numeric | numeric | NA | NA |
| Dossou, 2021  (Benin) | The current study assessed the impact of agriculture on the Oueme basin. | Climate/ Environment/ Disaster | mixed | 13 | No | numeric | ordinal | NA | NA |
| Felsenstein, 2014  (Israel) | To assesses the socioeconomic consequences of extreme coastal flooding events. | Climate/ Environment/ Disaster | census or geographical data | 4 | Yes | numeric | ordinal | NA | NA |
| Flanagan, 2011  (USA) | This paper describes the development of a social vulnerability index (SVI), from 15 census variables at the census tract level, for use in emergency management. | Climate/ Environment/ Disaster | census or geographical data | 15 | No | numeric | numeric | NA | NA |
| Abbas, 2021  (USA) | To examine the association between patient race/ethnicity and county-level vulnerability relative to patterns of hospice utilization | Health/ Medicine | - | - | - | - | - | Hospice utilization | Negative |
| An, 2015  (USA) | To examine the relationship between residential county social vulnerability and leisure-time physical inactivity among US adults. | Health/ Medicine | - | - | - | - | - | Physical inactivity | Positive |
| Angelidou, 2021  (USA) | To ascertain the percentage of neonates who were born to mothers with positive SARS-CoV-2 test results during the birth hospitalization, the clinical and sociodemographic factors associated with neonatal test result positivity, and the clinical and virological outcomes for newborns during hospitalization and 30 days after discharge | Health/ Medicine | - | - | - | - | - | Neonates with positive SARS-CoV-2 test results | Positive |
| Arling, 2021  (USA) | To assess the performance of the CDC SVI in classifying counties according to their COVID-19 mortality rates | Health/ Medicine | - | - | - | - | - | COVID-19 mortality rates and disaster loss | No association |
| Azap, 2021  (USA) | To characterize receipt of surgery and chemotherapy among Medicare beneficiaries with a diagnosis of early-stage pancreatic adenocarcinoma cancer (PDAC) relative to race/ethnicity and social vulnerability | Health/ Medicine | - | - | - | - | - | Receipt of surgery and chemotherapy | Negative |
| Azap, 2020  (USA) | To characterize possible differences in "textbook outcome," a composite measure of quality, relative to social vulnerability index | Health/ Medicine | - | - | - | - | - | Surgical textbook outcome | Negative |
| Azap, 2021  (USA) | To understand the association of County-Level Vulnerability, Patient-Level Race/Ethnicity, and Receipt of Surgery for Early-Stage Hepatocellular Carcinoma | Health/ Medicine | - | - | - | - | - | Receipt of Surgery | Negative |
| Barry, 2021  (USA) | To ascertain whether inequities in COVID-19 vaccination coverage with respect to county-level SVI have persisted, overall and by urbanicity. | Health/ Medicine | - | - | - | - | - | inequities in COVID-19 vaccination coverage | Positive |
| Ibrahim, 2021  (USA) | To examine the relationship between neighborhood social vulnerability and cardiovascular risk (hypertension and obesity) among Black/African American women. | Health/ Medicine | - | - | - | - | - | Cardiovascular risk (hypertension and obesity) | Positive |
| Benin, 2021  (USA) | To evaluate if facility-level vaccination after an initial vaccination clinic was independently associated with COVID-19 incidence adjusted for other factors in January 2021 among nursing home residents. | Health/ Medicine | - | - | - | - | - | Covid-19 incidence | Positive |
| Biggs, 2021  (USA) | To examine the association between the Centers for Disease Control and Prevention (CDC)'s Social Vulnerability Index (SVI) and COVID-19 incidence among Louisiana census tracts. | Health/ Medicine | - | - | - | - | - | Covid-19 incidence | Positive |
| Bilal, 2021  (USA) | To explore the emergence of spatial inequities in COVID-19 testing, positivity, confirmed cases, and mortality in New York, Philadelphia, and Chicago during the first 6 months of the pandemic. | Health/ Medicine | - | - | - | - | - | COVID-19 testing, positivity, confirmed cases, and mortality | Positive |
| Bogart, 2022  (USA) | We examined the extent to which socio-demographic and health-related background characteristics, medical mistrust, the perceived need for vaccination, confidence in vaccine efficacy and safety, social norms for vaccination, and neighborhood-level social vulnerability factors were associated with intentions to get vaccinated for COVID-19. | Health/ Medicine | - | - | - | - | - | intention or willingness to get vaccinated | Positive |
| Bozorgi, 2021  (USA) | To describe the geographic variation of spatial accessibility to opioid treatment programs (OTPs) and identifies areas with poor access to care in South Carolina. | Health/ Medicine | - | - | - | - | - | NA | NA |
| Bruckhaus, 2022  (USA) | To characterize the scope of vaccine inequity in California counties through modeling the trends of vaccination using the Social Vulnerability Index. | Health/ Medicine | - | - | - | - | - | Covid-19 vaccine coverage | Mixed |
| Carmichael, 2019  (USA) | To compare cholecystectomy patients presenting emergently versus electively. | Health/ Medicine | - | - | - | - | - | Emergent vs elective surgery | Positive |
| Carmichael, 2020  (USA) | To assess the performance of the Social Vulnerability Index compared with three similar measures used in the surgical literature: Area Deprivation Index, Community Needs Index, and Distressed Communities Index. | Health/ Medicine | - | - | - | - | - | NA | NA |
| Carter, 2021  (USA) | To assess interest and ability to participate in the Living Donor Navigator Program by the degree of social vulnerability. | Health/ Medicine | - | - | - | - | - | Willingness to participate in living donor program | Negative |
| Chang, 2021  (USA) | To examine differences in telehealth use and barriers to adoption among primary care practices and how those differences are influenced by the socioeconomic characteristics of their communities | Health/ Medicine | - | - | - | - | - | Telehealth | Positive |
| Crook, 2021  (USA) | To examine the uptake of several newly allowable benefits in 2021 as well as geographic differences in benefit offerings between areas by urbanicity, MA penetration, and social vulnerability. | Health/ Medicine | - | - | - | - | - | Benefits | Positive |
| Cunningham, 2021  (USA) | A resource assignment framework is developed as a coupled-state transition and linear optimization model that assists planners in optimally allocating constrained resources and satisfying mental health recovery priorities post-disaster. | Mixed | - | - | - | - | - | NA | NA |
| Dalmacy, 2022  (USA) | To examine the association of social vulnerability with the likelihood of experiencing fragmentation of postoperative care (FPC) after hepatopancreatic surgery. | Health/ Medicine | - | - | - | - | - | Fragmented post operative care | Positive |
| Dargin, 2021  (USA) | This research uses point of interest visitations as location intelligence data provided by SafeGraph together with Social Vulnerability Index and historical flood data to examine the critical intersection of natural hazard planning and response and the COVID-19 pandemic to assess the risks of a compound hazard situation. | Mixed | - | - | - | - | - | NA | NA |
| Dasgupta, 2020  (USA) | County-level data on COVID-19 cases during June 1-July 25, 2020 and from the 2018 CDC social vulnerability index (SVI) were analyzed to examine associations between social vulnerability and hotspot detection and to describe incidence after hotspot detection. | Health/ Medicine | - | - | - | - | - | At risk area to COVID-19 outbreak | Positive |
| Dekker, 2021  (USA) | To examine the impact of telemedicine in the disadvantaged population. | Health/ Medicine | - | - | - | - | - | Show or no-show to health appt | Positive |
| Delanois, 2021  (USA) | To investigate the association between demographic data, health status, and SDOHs on 30-day length of stay (LOS) and TCOC after this procedure. | Health/ Medicine | - | - | - | - | - | Post surgical outcome | No association |
| Diaz, 2021  (USA) | To assess the association of county-level vulnerability with the probability of having a non-elective colon resection | Health/ Medicine | - | - | - | - | - | Elective versus non-elective operation | Positive |
| Diaz, 2021  (USA) | To define the impact of high- versus low-quality hospitals on the risk of adverse outcomes among patients undergoing hepatopancreatic surgery relative to social vulnerability | Health/ Medicine | - | - | - | - | - | Post surgical outcome | Positive |
| Diaz, 2021  (USA) | To determine the neighborhood level characteristics that may be associated with travel patterns and utilization of high-volume hospitals | Health/ Medicine | - | - | - | - | - | Operation at a high-volume hospital | Negative |
| Diaz, 2021  (USA) | To examine postoperative outcomes following resection of lung and colon cancer | Health/ Medicine | - | - | - | - | - | Postoperative outcomes | Positive |
| Diaz, 2021  (USA) | The probability that a patient received care at a high-volume hospital and postoperative outcomes stratified by the social vulnerability of the patient's county of residence was examined. | Health/ Medicine | - | - | - | - | - | Postoperative outcomes and operation at a high-volume hospital | Positive |
| Diaz, 2021  (USA) | To characterize the association between patient county-level vulnerability with postoperative outcomes. | Health/ Medicine | - | - | - | - | - | Postoperative outcomes | Positive |
| Estrella, 2021  (USA) | We examined whether social vulnerability is associated with increased incidence of perforated appendicitis. | Health/ Medicine | - | - | - | - | - | Incidence of perforated appendicitis | No association |
| Fergen, 2021  (USA) | We map four themes of social vulnerability for the GLB by using the Center for Disease Control’s Social Vulnerability Index (CDC SVI) for every county in the basin and compare mean scores for each sub-basin to assess inter-basin differences | Climate/ Environment/ Disaster | - | - | - | - | - | NA | NA |
| Freese, 2021  (USA) | To determine the association between the Center for Disease Control and Prevention's (CDC) Social Vulnerability Index (SVI) with the risk of COVID-19-related mortality. | Health/ Medicine | - | - | - | - | - | Mortality related to COVID-19 | Positive |
| Fu, 2021  (USA) | We applied a geographically and temporally weighted regression (GTWR) to analyze the spatiotemporal pattern of community stay-at-home behaviors against social vulnerability indicators at the census tract level in New York City from March to August 2020. | Health/ Medicine | - | - | - | - | - | NA | NA |
| Gay, 2016  (USA) | This study examined the utility of using the SVI to explain variation in youth fitness, including aerobic capacity and body mass index. | Health/ Medicine | - | - | - | - | - | Youth fitness (aerobic capacity and BMI) | Positive |
| Gharpure, 2021  (USA) | To assess vaccine uptake in these communities and identify characteristics that might impact uptake. | Health/ Medicine | - | - | - | - | - | Covid Vaccine uptake | Positive |
| Givens, 2021  (USA) | To investigate the association of the Social Vulnerability Index for each patient's residence during pregnancy, personal clinical risk factors, and preterm birth. | Health/ Medicine | - | - | - | - | - | Preterm birth | Positive |
| Godfrey, 2021  (USA) | To examine individual characteristics, motivations, and geographic locations of patients receiving abortion care through the Aid Access platform. | Health/ Medicine | - | - | - | - | - | NA | NA |
| Grunwell, 2022  (USA) | Composite measures of social determinants of health and readmission outcomes were evaluated in a large regional cohort of 1,403 school-age children admitted to a pediatric intensive care unit (PICU) for asthma. | Health/ Medicine | - | - | - | - | - | PICU admissions/Asthma outcomes | Positive |
| Harrison, 2021  (USA) | To examine the factors associated with EMS refusal in relation to COVID-19 cases, public health interventions, EMS responses, and prehospital deaths. | Health/ Medicine | - | - | - | - | - | rate of EMS transport refusals | Positive |
| Hathaway, 2021  (USA) | To comment on community risk of rural minority population during COVID-19. | Health/ Medicine | - | - | - | - | - | NA | NA |
| Hughes, 2021  (USA) | To examine equity in vaccine coverage | Health/ Medicine | - | - | - | - | - | Vaccine equity | Mixed |
| Hyer, 2021  (USA) | To characterize differences in "textbook outcomes" (TO) relative to social vulnerability among Medicare beneficiaries who underwent operations for cancer. | Health/ Medicine | - | - | - | - | - | Surgical textbook outcome | Negative |
| Hyer, 2021  (USA) | To characterize the role of patient social vulnerability relative to hospital racial/ethnic integration on postoperative outcomes among patients undergoing pancreatectomy. | Health/ Medicine | - | - | - | - | - | Surgical textbook outcome | Negative |
| Islam, 2021  (USA) | To examine incidence and mortality from COVID-19 | Health/ Medicine | - | - | - | - | - | Incidence and mortality from COVID-19 | Positive |
| Islam, 2021  (USA) | We examined the temporal association of county-level Social Vulnerability Index (SVI), a percentile-based measure of social vulnerability to disasters, its subcomponents and race/ethnic composition with COVID-19 incidence and mortality in the USA in the year starting in March 2020 | Health/ Medicine | - | - | - | - | - | COVID-19 incidence and mortality | Positive |
| Javalkar, 2021  (USA) | To characterize the socioeconomic and racial and/or ethnic disparities impacting the diagnosis and outcomes of multisystem inflammatory syndrome in children (MIS-C). | Health/ Medicine | - | - | - | - | - | MIS-C diagnosis and severity | Mixed |
| Johnson, 2021  (USA) | This study summarizes the results from fitting a Bayesian hierarchical spatiotemporal model to coronavirus disease 2019 (COVID-19) cases and deaths at the county level in the United States for the year 2020. | Health/ Medicine | - | - | - | - | - | Covid-19 cases and mortality | Positive |
| Johnson, 2018  (USA) | Our research presents a web-based tool for providing data to decision-makers in support of local and regional adaptation planning processes. | Climate/ Environment/ Disaster | \| - \| - \| - \| - \| - \| \| --- \| --- \| --- \| --- \| --- \| | - | - | - | - | NA | NA |
| Jones, 2020  (USA) | Using data from DC Health, the Washington, DC, department of public health, this study investigated associations between neighborhood social, built, and natural environments and rates of asthma-related healthcare encounters by ZIP code between 2014 and 2017. | Health/ Medicine |  |  |  |  |  | NA | NA |
| Karaye, 2020  (USA) | This study estimates the association between case counts of COVID-19 infection and social vulnerability in the U.S., identifying counties at increased vulnerability to the pandemic. | Health/ Medicine | - | - | - | - | - | COVID-19 case count | Positive |
| Karmakar, 2021  (USA) | To examine the association between county-level sociodemographic risk factors and US COVID-19 incidence and mortality. | Health/ Medicine | - | - | - | - | - | NA | NA |
| Khan, 2021  (USA) | To examine whether underlying social vulnerabilities of counties influence premature cardiovascular disease mortality is uncertain. | Health/ Medicine | - | - | - | - | - | NA | NA |
| Khazanchi, 2020  (USA) | To examine the relationship between social vulnerability and COVID-19 diagnosis and mortality in rural and urban communities remains unknown. | Health/ Medicine | - | - | - | - | - | NA | NA |
| Killian, 2021  (USA) | To determine if racial disparities persist in LDKT independent of community-level vulnerability. | Health/ Medicine | - | - | - | - | - | NA | NA |
| Killian, 2022  (USA) | To examine the association between social vulnerability and living donor navigator self-advocacy | Health/ Medicine | - | - | - | - | - | Self-advocacy | Positive |
| Lai, 2019  (USA) | We hypothesized that SVI is associated with SCD-related severity and utilization. | Health/ Medicine | - | - | - | - | - | Healthcare utilization | Positive |
| LeRose, 2021  (USA) | Our primary objective was to determine whether a correlation existed between the SVI and PPE supply shortages in Michigan SNFs. Additionally, we analyzed the potential relationship between the SVI and the number of COVID-19 cases and mortality rate. | Mixed | - | - | - | - | - | PPE shortages, number of COVID-19 cases, mortality. | Positive |
| Lotfata, 2019  (USA) | In this study, we analyze the spatial patterns of the social vulnerability index (SVI) in each flood zone within Louisiana parishes of East Baton Rouge, Ascension, and Livingston. | Climate/ Environment/ Disaster | - | - | - | - | - | NA | NA |
| McAlarnen, 2021  (USA) | We describe the utilization of virtual visits by patients with gynecologic malignancies and assess their social vulnerability. | Health/ Medicine | - | - | - | - | - | Telemedicine usage | No association |
| Mock, 2021  (USA) | We describe the utilization of virtual visits by patients with gynecologic malignancies and assess their social vulnerability. | Health/ Medicine | - | - | - | - | - | Telecare utilization | Negative |
| Morgan, 2020  (USA) | To examine if pediatric trauma patients high on the social vulnerability index would have significantly lower rates of rehab admission following admission to a hospital for traumatic injury. | Health/ Medicine | - | - | - | - | - | Rehab admission | Negative |
| Nayak, 2020  (USA) | To examine the association of Social Vulnerability Index (SVI), a percentile-based measure of county-level social vulnerability to disasters, and its sub-components (socioeconomic status, household composition, minority status, and housing type/transportation accessibility) with the case fatality rate (CFR) and incidence of COVID-19. | Health/ Medicine | - | - | - | - | - | COVID-19 incidence and case fatality rate | Positive |
| Neelon, 2021  (USA) | We examined temporal trends among counties with high and low social vulnerability to quantify disparities in trends over time. | Health/ Medicine | - | - | - | - | - | COVID-19 incidence and death rates. | Positive |
| Oates, 2021  (USA) | We assessed the relationship between social vulnerability and COVID-19 testing rates, test positivity, and incidence. | Health/ Medicine | - | - | - | - | - | COVID-19 testing, incidence, positivity. | Mixed |
| Papageorge, 2022  (USA) | We examined the effect of Medicaid expansion on the diagnosis of HCC and associations with county-level social vulnerability. | Health/ Medicine | - | - | - | - | - | Early-stage cancer diagnosis | Negative |
| Paro, 2021  (USA) | To identify distinct profiles of social vulnerability among Medicare beneficiaries and define the association of these profiles with postoperative outcomes. | Health/ Medicine | - | - | - | - | - | Postoperative outcomes | Positive |
| Phelos, 2021  (USA) | To determine if these indices correlate with injury fatality rates in the US. | Climate/ Environment/ Disaster | - | - | - | - | - | Fatality rates | Positive |
| Puvvula, 2021  (USA) | This study evaluated the association between atrazine in surface and groundwater, in relation to the incidence of pediatric cancer in Nebraska watersheds over 30 years. | Health/ Medicine | - | - | - | - | - | NA | NA |
| Regmi, 2021  (USA) | To use the parameters of social vulnerability index (SVI) to observe their association with the 30-day hospital readmissions in the heart failure population. | Health/ Medicine | - | - | - | - | - | 30-day hospital readmission | Positive |
| Rickless, 2021  (USA) | This study explored demographic indicators of vulnerability for patients from the Hurricane Harvey impact area who sought medical care in Houston and in DFW. | Mixed | - | - | - | - | - | Presenting to medical facility following natural disaster | Positive |
| Saia, 2020  (USA) | We compared spatial distributions of high-risk subbasins based on SWAT results, SVI results, and the integration of SWAT and SVI results using a risk matrix. | Climate/ Environment/ Disaster | - | - | - | - | - | Streamflow projections | Mixed |
| Sharareh, 2020  (USA) | To highlight areas in Utah vulnerable to a Hepatitis C virus (HCV) outbreak. | Health/ Medicine | - | - | - | - | - | HCV outbreaks among persons who inject drugs | Positive |
| Steinkamp, 2021  (USA) | The purpose of this study was to determine what these terms (equity, diversity and inclusion) mean with respect to health care, and whether we are manifesting them in our medical practices. | Health/ Medicine | - | - | - | - | - | NA | NA |
| Strully, 2022  (USA) | To inform vaccine equity interventions, this analysis investigates spatially varying associations between county social vulnerability and influenza vaccination rate among Medicare recipients. | Health/ Medicine | - | - | - | - | - | Influenza vaccination rates | Negative |
| Thakore, 2021  (USA) | To understand how strategic vaccine site placement may benefit high vulnerability populations. | Health/ Medicine | - | - | - | - | - | COVID-19 vaccine site density and vaccination rates | Mixed |
| Troppy, 2021  (USA) | To understand the spatial heterogeneity of associations between social determinants and the use of SARS-CoV-2 testing. | Health/ Medicine | - | - | - | - | - | COVID testing | Mixed |
| Tummalapalli, 2021  (USA) | To examine whether disparities in COVID-19 incidence related to race/ethnicity and socioeconomic factors exist in the hemodialysis population. | Health/ Medicine | - | - | - | - | - | Acquiring COVID-19 | Mixed |
| Turek-Hankins, 2020  (USA) | We examined racial/ethnic differences in COVID-19 incidence among patients on hemodialysis in New York City during the first wave of the COVID-19 pandemic and assessed if SVI explained racial/ethnic differences in COVID-19 incidence. | Climate/ Environment/ Disaster | - | - | - | - | - | Acquired COVID-19 | Positive |
| Upchurch, 2022  (USA) | [We investigated racial/ethnic differences by gender in correlates of COVID-19 infection among veterans seeking health care services at the Veterans Health Administration.](https://www-sciencedirect-com.ezproxy.library.dal.ca/topics/medicine-and-dentistry/veterans-health) | Health/ Medicine | - | - | - | - | - | Testing positive for COVID-19 | Mixed |
| Vickers, 2021  (USA) | To comment on individual patient vulnerability relative to surgical outcomes. | Health/ Medicine | - | - | - | - | - | NA | NA |
| Vo, 2020  (USA) | To measure the accessibility levels for three emergency response thresholds: zero to four minutes, four to eight minutes, and eight to fifteen minutes. | Mixed | - | - | - | - | - | Healthcare accessibility | Positive |
| Wang, 2022  (USA) | This study aims to examine the spatially varying relationships between social vulnerability factors and COVID-19 cases and deaths in the contiguous United States. | Health/ Medicine | - | - | - | - | - | COVID-19 cases and deaths | Positive |
| Wang, 2021  (USA) | The study aims to examine the vaccination inequities among different population groups for people aged 65+. | Health/ Medicine | - | - | - | - | - | Vaccination rates | Negative |
| Yee, 2019  (USA) | This ecological study explores the application of the SVI as a predictor of teen pregnancy rates across counties in the United States (U.S.) and identifies areas with greatest need for community-based interventions. | Health/ Medicine | - | - | - | - | - | Teen birth rate | Positive |
| Yee, 2021  (USA) | We aim to analyze the effects of social determinants of health on COVID-19 outcomes and public health responses. | Health/ Medicine | - | - | - | - | - | NA | NA |
| Zachrison, 2021  (USA) | To describe patient characteristics associated with successful transition from in-person to virtual care, and video vs audio-only participation. | Health/ Medicine | - | - | - | - | - | Patient characteristics by visit type | Mixed |
| Zottarelli, 2021  (USA) | To examine the effects of excessive heat and community-level social vulnerability on morbidity in San Antonio, Texas. | Climate/ Environment/ Disaster | - | - | - | - | - | Morbidity | Positive |
| Fraser, 2021  (Japan) | This paper introduces a methodology to create the Japanese Municipal Social Capital Index and Social Vulnerability Index, for every year from 2000 to 2017. | Climate/ Environment/ Disaster | census or geographical data | 19 | No | numeric | ordinal | NA | NA |
| Frigerio, 2016  (Italy) | To apply a proven method for assessing social vulnerability at the national scale, while considering the contribution of the socioeconomic and demographic factors that affect the Italian population. | Climate/ Environment/ Disaster | census or geographical data | 12 | No | numeric | ordinal | NA | NA |
| Frigerio, 2018  (Italy) | Evaluate the pattern of social vulnerability over time in Italy. | Climate/ Environment/ Disaster | census or geographical data | 16 | Yes | numeric | numeric | NA | NA |
| Frigerio, 2019  (Italy) | This paper seeks to identify those areas that proved socially vulnerable to the earthquake that struck central Italy on 24 August 2016. | Climate/ Environment/ Disaster | census or geographical data | 16 | Yes | numeric | ordinal | NA | NA |
| Gautam, 2017  (Nepal) | This study aims to quantify the social vulnerability on a local scale, considering all 75 districts using the available census. | Climate/ Environment/ Disaster | census or geographical data | 13 | Yes | numeric | numeric | NA | NA |
| Ge, 2017  (China) | This paper explored a new approach regarding social vulnerability to climate change. | Climate/ Environment/ Disaster | census or geographical data | 24 | Yes | numeric | numeric | NA | NA |
| Ge, 2017  (China) | A social vulnerability assessment trial was carried out for Chinese coastal cities at the county level. First, the 10 factors having the most influence on social vulnerability were identified. | Climate/ Environment/ Disaster | - | - | - | - | - | NA | NA |
| Ge, 2017  (China) | To propose a new conceptual framework for urban social vulnerability assessment based on network theory, where a new dimension of social vulnerability (connectivity) was added into the framework. | Climate/ Environment/ Disaster | mixed | 19 | No | numeric | numeric | NA | NA |
| Ge, 2013  (China) | This paper presents a new method for quantifying SV based on the projection pursuit cluster (PPC) model. A reference social vulnerability index (SVI) at the county level was created for the Yangtze River Delta area in China for 1995, 2000, 2005, and 2009. | Climate/ Environment/ Disaster | census or geographical data | 19 | No | numeric | numeric | NA | NA |
| Ge, 2019  (China) | This paper examines social vulnerability and inequality through a joint analysis of urban agglomerations. | Climate/ Environment/ Disaster | census or geographical data | 24 | Yes | numeric | numeric | NA | NA |
| Godin, 2019  (Canada) | We sought to understand the association between social vulnerability and the odds of long-term care (LTC) placement within 30 days of discharge following admission to an acute care facility and whether this association varied based on age, sex, or pre-admission frailty. | Health/ Medicine | clinical data | 18 | No | numeric | categorical | LTC placement | Positive |
| Grasso, 2014  (Samoa) | To investigate the notion of social vulnerability and measure its dimensions in Samoa through a specific index: the Samoa Social Vulnerability Index (SSVI). | Climate/ Environment/ Disaster | census or geographical data | 15 | No | numeric | numeric | NA | NA |
| Gu, 2018  (China) | This paper presents a hierarchical pattern of urban social vulnerability by a SoVI assessment of the 5432 neighborhoods (residential committee, or *juwei* in Chinese) in Shanghai metropolitan, China. | Climate/ Environment/ Disaster | census or geographical data | 17 | No | numeric | ordinal | NA | NA |
| Guo, 2020  (Hong Kong & China) | This study aims to explore the spatial variations in the elderly suicide rates and their correlates in Hong Kong. | Health/ Medicine | mixed | 7 | No | numeric | numeric | NA | NA |
| Gupta, 2020  (India) | [This study systematically analysed important components of vulnerability and mapped them by weight for four altitude zones in the Indian Himalayas.](https://www.sciencedirect.com/topics/earth-and-planetary-sciences/himalayas) | Climate/ Environment/ Disaster | representative survey | 15 | No | numeric | numeric | NA | NA |
| Hazards and Vulnerability Research Institute at the University of South Carolina, 2016  (USA) | To describe the Social Vulnerability Index (SoVI®) which measures the social vulnerability of U.S. counties to environmental hazards. | Climate/ Environment/ Disaster | census or geographical data | 29 | No | numeric | ordinal | NA | NA |
| Al Rifat, 2021  (USA) | To explore and understand the intersection of meteorological storm characteristics, physical characteristics of the areas impacted, and social-economic vulnerability variables as predictors of county-level inland property damage. | Climate/ Environment/ Disaster | - | - | - | - | - | Property damage or loss | No association |
| Jagarnath 2020  (South Africa) | To investigate current and projected future heat risk, expressed as a heat stress exposure index using high-resolution climate change projections, and a social vulnerability index, to identify areas of potential future heat stress risk in the Durban (eThekwini) metropolitan area, South Africa. | Climate/ Environment/ Disaster | census or geographical data | 20 | No | numeric | ordinal | NA | NA |
| Kamiohkawa, 2021  (Phillipines) | This study empirically investigated the social vulnerability of two municipalities of Laguna Province, Philippines, on the impacts of natural disasters associated with climate change. | Climate/ Environment/ Disaster | representative survey | 21 | Yes | numeric | numeric | NA | NA |
| Karunarathne, 2020  (Sri Lanka) | We shed light on an influential mechanism in order to measure social vulnerability to flooding in both rural and urban areas. We developed the multi-facet composite social vulnerability index (MFCSVI). | Climate/ Environment/ Disaster | representative survey | 31 | Yes | numeric | ordinal | NA | NA |
| Kim, 2020  (Indonesia) | This paper investigates social vulnerability to foods at the municipality level on Java. | Climate/ Environment/ Disaster | census or geographical data | 8 | No | numeric | ordinal | NA | NA |
| Kirby, 2019  (The Netherlands) | This study utilizes fine-scale data to construct a social vulnerability index for 147 districts of the Dutch province of Zeeland. | Climate/ Environment/ Disaster | mixed | 7 | Yes | numeric | ordinal | NA | NA |
| Koks, 2015  (The Netherlands) | This study shows how a joint assessment of hazard, exposure and social vulnerability provides valuable information for the evaluation of flood risk management strategies. | Climate/ Environment/ Disaster | census or geographical data | 8 | No | numeric | ordinal | NA | NA |
| Kumar, 2020  (India) | Vulnerability index of a community has to be calculated considering physical, social, economic and environmental factors associated with the community. This research paper tries to find out an integrated social vulnerability factor. | Climate/ Environment/ Disaster | mixed | 22 | Yes | numeric | numeric | NA | NA |
| Lawal, 2015  (Nigeria) | This study sought to develop a spatially explicit index of social vulnerability, thus addressing the dearth of research in this area in sub-Saharan Africa. | Climate/ Environment/ Disaster | mixed | 6 | No | numeric | ordinal | NA | NA |
| Lee, 2014  (Taiwan) | [To promote sustainable development, this study offers a case study of developmental planning in Chiayi, Taiwan and a review of the relevant literature to propose a framework of social vulnerability indicators at the township level.](https://www-sciencedirect-com.ezproxy.library.dal.ca/topics/earth-and-planetary-sciences/environmental-impact-assessment) | Climate/ Environment/ Disaster | administrative data | 13 | No | numeric | numeric | NA | NA |
| Letsie, 2015  (Lesotho) | To assess social vulnerability of communities to natural hazards by applying a place-based social vulnerability index developed for the United States, to the Lesotho context. | Climate/ Environment/ Disaster | mixed | 25 | No | numeric | ordinal | NA | NA |
| Lin, 2016  (Taiwan) | This study applied spatial autocorrelation statistics to analyze the spatial association of vulnerability among townships in Taiwan. | Climate/ Environment/ Disaster | mixed | 14 | Yes | numeric | ordinal | NA | NA |
| Liu, 2016  (China) | In this study, a household social vulnerability index (HSVI) to flood hazards was developed and used to assess the social vulnerability of rural households in western mountainous regions of Henan province, China. | Climate/ Environment/ Disaster | mixed | 8 | Yes | numeric | categorical | NA | NA |
| Liu, 2021  (Taiwan) | This study examined effects between SVI status and two genotypes, apolipoprotein E (ApoE) and Serotonin transporter genotyping (5-HTTLPR), on all-cause mortality. | Health/ Medicine | representative survey | 32 | No | numeric | ordinal | Mortality | Positive |
| Lixin, 2017  (China) | In this study, five social vulnerability indicators indexes (social network, community administration, community participation, community disaster prevention, and social support) are selected to build a community-based social vulnerability index (SoVI). | Climate/ Environment/ Disaster | representative survey | 21 | Yes | numeric | ordinal | NA | NA |
| Lixin, 2014  (China) | This paper presents a mathematical model to establish a model of social vulnerability index (SoVI), which includes 12 social variables, and the regional social vulnerability to natural hazards was formulated by them. | Climate/ Environment/ Disaster | mixed | 12 | Yes | numeric | ordinal | NA | NA |
| Maharani, 2017  (South Korea) | This study utilized SoVI and SOM to examine social vulnerability in the South Korea | Climate/ Environment/ Disaster | administrative data | 12 | Yes | numeric | ordinal | NA | NA |
| Martínez, 2020  (Chile) | An event with characteristics similar to those of the 1730 earthquake (Mw 9.1) was modelled considering the worst-case scenario for the coast of central Chile | Climate/ Environment/ Disaster | census or geographical data | 23 | Yes | numeric | ordinal | NA | NA |
| Mavhura, 2017  (Zimbabwe) | The study demonstrates an accessible means to assessing the spatial variation of social vulnerability to flood hazards and related for the context of Muzarabani district in northeast Zimbabwe. | Climate/ Environment/ Disaster | mixed | 17 | Yes | numeric | ordinal | NA | NA |
| Mavhura, 2018  (Zimbabwe) | to analyse the overall and subcomponents of resilience to identify wards that needed policy intervention. | Climate/ Environment/ Disaster | - | - | - | - | - | NA | NA |
| Mavhura, 2021  (Zimbabwe) | to quantify social vulnerability and model its underlying drivers respectively. | Climate/ Environment/ Disaster | - | - | - | - | - | NA | NA |
| Mavromatidi, 2018  (France) | The aim of this study is the superimposition of two widely used empirical indexes – the Coastal Sensitivity Index and the Social Vulnerability Index. | Climate/ Environment/ Disaster | administrative data | 10 | No | numeric | ordinal | NA | NA |
| de Medeiros, 2016  (Brazil) | To map areas of social vulnerability and natural hazards in Natal, taking into account the interrelationships between social vulnerabilities and differentiated exposure to natural hazards. | Climate/ Environment/ Disaster | census or geographical data | 26 | No | numeric | numeric | NA | NA |
| Berra, 2017  (Brazil) | To analyze the spatial risk of tuberculosis mortality and to verify associations in high-risk areas with social vulnerability. | Health/ Medicine | - | - | - | - | - | Tuberculosis mortality | Positive |
| Moyano, 2021  (Argentina) | To analyze the relationship between social vulnerability and the healthy use of leisure time in children and adolescents in urban contexts of Argentina. | Health/ Medicine | representative survey | 10 | Yes | numeric | ordinal | Use of leisure time | Mixed |
| Nahas, 2000  (Brazil) | To develop a map of social exclusion. | Health/ Medicine | census or geographical data | 11 | Yes | numeric | numeric | NA | NA |
| Bendo, 2010  (Brazil) | To describe the prevalence of traumatic dental injury (TDI) and associated factors in the permanent incisors of Brazilian schoolchildren. | Health/ Medicine | - | - | - | - | - | TDI | SVI covariate (no association reported) |
| Bendo, 2010  (Brazil) | To investigate the association between treated/untreated TDI and the impact on the quality of life of 11-to-14-year-old Brazilian schoolchildren. | Health/ Medicine | - | - | - | - | - | TDI | SVI covariate (no association reported) |
| Bendo, 2012  (Brazil) | To test the association between social vulnerability and the prevalence of TDI. | Health/ Medicine | - | - | - | - | - | Untreated TDI | Negative |
| Fernandes Bolina, 2019  (Brazil) | To verify the occurrence and factors associated to social, individual and programmatic vulnerability among older adults. | Health/ Medicine | - | - | - | - | - | Education, living conditions and income | Negative |
| Freire-Maia, 2015  (Brazil) | To describe the association of oral health-related quality of life (OHRQoL) and domains (oral symptons, functional limitation, emotional- and social-well-being) of children with individual and contextual variables. | Health/ Medicine | - | - | - | - | - | OHRQoL | Negative |
| Jorge, 2015  (Brazil) | To determine the prevalence of tobacco, use and its association with types of friendship networks, socioeconomic status and gender among Brazilian adolescents. | Health/ Medicine | - | - | - | - | - | Tobacco use | Positive |
| Jorge, 2009  (Brazil) | To assess the epidemiology of traumatic dental injuries (TDI) to primary teeth in infants and toddlers between 1 and 3 years of age and investigate whether TDI was related to biological and social factors. | Health/ Medicine | - | - | - | - | - | Dental injury | Positive |
| Jorge, 2012  (Brazil) | To investigate the prevalence of dental trauma, etiological factors, predisposing factors, and associations with socioeconomic status and the risk of alcohol and illicit drug use among adolescents in the city of Belo Horizonte, Brazil. | Health/ Medicine | - | - | - | - | - | NA | NA |
| Jorge, 2018  (Brazil) | To evaluate the prevalence of alcohol consumption, binge drinking and their association with social capital and socioeconomic factors among Brazilian adolescents students. | Health/ Medicine | - | - | - | - | - | Binge-drinking behavior. | Positive |
| Martins, 2019  (Brazil) | To determine the prevalence of malocclusion in adolescents and to test its association with social vulnerability. | Health/ Medicine | - | - | - | - | - | Dental crowding / malocclusion | Positive |
| Martins, 2014  (Brazil) | To assess caries experience among Brazilian children aged eight to 10 years and determine social factors of this disease, through a multilevel approach. | Health/ Medicine | - | - | - | - | - | Dental caries | Positive |
| Martins, 2015  (Brazil) | To evaluate the impact of dental caries and social determinants in the Oral Health Related Quality of Life (OHRQoL) of children in Belo Horizonte, Brazil. | Health/ Medicine | - | - | - | - | - | OHRQoL | Negative |
| Martins-Oliveira, 2016  (Brazil) | To evaluate the possible alcohol dependence and related problems among adolescents and determined possible associations with socioeconomic factors and gender. | Health/ Medicine | - | - | - | - | - | Consumption of alcohol | Positive |
| Serra-Negra, 2013  (Brazil) | To analyze the association between children's tasks, personality traits and sleep bruxism. | Health/ Medicine | - | - | - | - | - | NA | NA |
| Serra-Negra, 2010  (Brazil) | To investigate the prevalence of sleep bruxism in Brazilian schoolchildren. | Health/ Medicine | - | - | - | - | - | NA | NA |
| Serra-Negra, 2009  (Brazil) | To assess the prevalence of sleep bruxism in children and the influence of psychosocial factors. | Health/ Medicine | - | - | - | - | - | Bruxism | No association |
| Serra-Negra, 2011  (Brazil) | To compare self-reported dental fear among dental students and patients at a School of Dentistry in Belo Horizonte, Brazil. | Health/ Medicine | - | - | - | - | - | NA | NA |
| Silva-Oliveira, 2014  (Brazil) | To establish the prevalence of inhalant use among adolescents and its association with marijuana use, alcohol consumption, socioeconomic status and gender. | Health/ Medicine | - | - | - | - | - | Marijuana use | No association |
| Viegas, 2010  (Brazil) | To assess the prevalence of traumatic dental injury (TDI) in primary teeth and determine predisposing factors. | Health/ Medicine | - | - | - | - | - | TDI | No association |
| Zarzar, 2012  (Brazil) | To examine the prevalence of binge drinking/alcohol consumption and its association with different types of friendship networks, gender and socioeconomic status among students in Belo Horizonte, Minas Gerais, Brazil. | Health/ Medicine | - | - | - | - | - | Binge drinking | Positive |
| Nelson, 2015  (USA) | This paper describes and illustrates a hybrid method for creating a social vulnerability index (SVI) at a tax parcel level by utilizing supplementary information about tax parcels to link cadastral dasymetric mapping techniques and established social vulnerability indexing methods. | Climate/ Environment/ Disaster | census or geographical data | 37 (1^st^ SVI)  30 (2^nd^ SVI) | Yes | numeric | ordinal | NA | NA |
| Nguyen, 2017  (Vietnam) | This paper proposes an approach to social vulnerability assessment using new empirical definitions of Social Vulnerability Index (SVI) components and new mechanism to aggregate and account for causal relationships among these components. | Climate/ Environment/ Disaster | other | 26 | Can't tell | numeric | numeric | NA | NA |
| Nguyen, 2019  (Canada) | To describe social vulnerability, to examine its correlation with the number of chronic conditions, and to investigate which chronic conditions were significantly associated with the most socially vulnerable state in patients with multimorbidity. | Health/ Medicine | clinical data | 19 | No | numeric | ordinal | Chronic conditions | Positive |
| Nicholson, 2019  (USA) | This paper presents a new social vulnerability index construction approach that utilizes geographically weighted local regression modeling and spatial clustering to determine location-specific weights of vulnerability indicators | Climate/ Environment/ Disaster | census or geographical data | 24 | Yes | numeric | numeric | NA | NA |
| Ogie, 2020  (Australia) | To present a strength-based social vulnerability index that identifies the strengths that communities have that help minimise disaster risk exposure. | Climate/ Environment/ Disaster | census or geographical data | 18 | No | numeric | numeric | NA | NA |
| Oulahen, 2015  (Canada) | To describe the process of ground truthing a social vulnerability index with practitioners working in five municipalities in Metro Vancouver and how the index was then revised to reflect their input. | Climate/ Environment/ Disaster | census or geographical data | 20 | Yes | numeric | numeric | NA | NA |
| Prabhu, 2022  (Kenya) | To adapt a Social Vulnerability Index (SVI) originally developed in Canada for use in a study of older women living with or without HIV infection in Mombasa, Kenya. | Health/ Medicine | other | 16 | No | numeric | numeric | NA | NA |
| Quezeda-Hofflinger, 2019  (Peru & Chile) | To develop the Response Time by Social Vulnerability Index and to provide an example of the application of ReTSVI in a potential case of a severe flood event in Huaraz, Peru. | Climate/ Environment/ Disaster | representative survey | 20 | No | numeric | numeric | Evacuation time | Positive |
| Reckien, 2018  (USA) | To investigate the outcome of the variable addition—both with and without weighting of single vulnerability factors—and the variable reduction approach/model on social vulnerability indices calculated for New York City. | Climate/ Environment/ Disaster | mixed | 10 (1^st^ SVI)  9 (2^nd^ SVI) | Yes | numeric | numeric | NA | NA |
| Rifat, 2021  (USA) | To explore spatial distributions and patterns of COVID-19 case rates (cases/100,000 people) and mortality rates (deaths/100,000 people) and their disparities between urban and rural counties in the contiguous US. | Health/ Medicine | census or geographical data | 28 | No | numeric | numeric | Covid-19 case and mortality rates | Positive |
| Roder, 2017  (Italy) | To show the application of the SoVI to the floodplain of northern Italy, based on the use of 15 census variables. | Climate/ Environment/ Disaster | mixed | 12 | Yes | numeric | ordinal | NA | NA |
| Rodriquez, 2018  (Palestine) | To assess the social vulnerability and resilience level of the city of Nablus, an important urban center in Palestine | Climate/ Environment/ Disaster | census or geographical data | 28 | No | numeric | numeric | NA | NA |
| Roncancio, 2020  (Colombia) | To understand the pre-existing social vulnerability throughout the territory as a first step in national disaster risk reduction and climate change adaptation planning. | Climate/ Environment/ Disaster | census or geographical data | 29 | Yes | numeric | ordinal | NA | NA |
| Sánchez-Garrido, 2021  (Mexico) | To analyze the association of the SVI with mortality and disability in Mexican middle-aged and older adults | Health/ Medicine | administrative data | 42 | No | numeric | numeric | Mortality, disability | Positive |
| São Paulo Índice Paulista de Vulnerabilidade Social, 2010  (Brazil) | To describe the Indice Paulista de Vulnerabilidade Social (IPVS) | Health/ Medicine | census or geographical data | 9 | No | numeric | numeric | NA | NA |
| Alves, 2020  (Brazil) | To analyze the association of cases of childhood tuberculosis with social vulnerability. | Health/ Medicine | - | - | - | - | - | Tuberculosis cases in children | Positive |
| Andrade, 2022  (Brazil) | To analyse the spatiotemporal dynamics of human visceral leishmaniasis (HVL) in an endemic state in the Northeast Region of Brazil and its spatial correlation with the Social Vulnerability Index (SVI) and the Municipal Human Development Index. | Health/ Medicine | - | - | - | - | - | HVL incidence rat. | Positive |
| Arroyo, 2017  (Brazil) | To identify spatial and space-time clusters of risk for tuberculosis and to characterize them according to social vulnerability. | Health/ Medicine | - | - | - | - | - | NA | NA |
| Baquero, 2018  (Brazil) | To evaluate the association between interpersonal violence notifications, animal abuse notifications and an index of social vulnerability in São Paulo City, on a geographic scale, using Bayesian spatial models. | Other | - | - | - | - | - | NA | NA |
| da Cunha, 2017  (Brazil) | To investigate how social vulnerability and oral-health status factors affect QoL in 15–19 years olds who participated in the “SB São Paulo 2015” state survey. | Health/ Medicine | - | - | - | - | - | Oral health status | No association |
| de Jesus, 2018  (Brazil) | To analyze the relationship between of frailty and the family social relationships of the elderly in a context of social vulnerability. | Health/ Medicine | - | - | - | - | - | NA | NA |
| de Jesus, 2020\  (Brazil) | To report the impact of the 12-year rotavirus vaccine program on diarrhea mortality and hospitalizations and their correlation to socioeconomic indicators. | Health/ Medicine | - | - | - | - | - | Vaccination coverage | Mixed |
| Franco, 2021  (Brazil) | To characterize the food environment within subway stations in São Paulo and describe the availability of food and drinks according to the social vulnerability of the area where the stations are located. | Health/ Medicine | - | - | - | - | - | NA | NA |
| Freitas, 2016  (Brazil) | To evaluate the contextual effects of social vulnerability over anthropometric indexes related to global and central obesity in adults living in Ribeirão Preto, Brazil, in 2006. | Health/ Medicine | - | - | - | - | - | BMI and waist circumference | Positive |
| Macedo, 2015  (Brazil) | To analyse situations of socio-environmental vulnerability on intra-urban scale, in a group of 62 municipalities in the three main metropolitan regions of the Macro-metropolis of São Paulo State. | Climate/ Environment/ Disaster | - | - | - | - | - | NA | NA |
| Martinez, 2011  (Brazil) | To investigate the spatial association between teenage pregnancy rates and socioeconomic characteristics of municipalities in São Paulo State | Health/ Medicine | - | - | - | - | - | Teenage pregnancy | Negative |
| Nakamura, 2016  (Brazil) | To verify association between public and private places for engaging in different types of physical activity in adults of Rio Claro City, Brazil. | Health/ Medicine | - | - | - | - | - | Physical activity and physical leisure time | Positive |
| Sedrez, 2019  (Brazil) | To understand spatial segregation in the city of São Paulo (a city known by its social inequality and urban poverty) by scraping social media tags of emotions. | Other | - | - | - | - | - | NA | NA |
| Schmitz, 2001  (Austria) | To establish a 6-point scale on the basis of the patient's history to estimate prognosis of a patient. | Health/ Medicine | clinical data | 6 | No | numeric | categorical | Diagnosis, duration of therapy and first contact with Community Mental Health Care Units | Positive |
| Shaji, 2021  (India) | To compute CSoVI for the coast of Thiruvananthapuram | Climate/ Environment/ Disaster | census or geographical data | 11 | No | numeric | numeric | NA | NA |
| Sharma, 2018  (USA) | To identify vulnerable neighborhoods in the City of Chicago. | Climate/ Environment/ Disaster | census or geographical data | 28 | Yes | ordinal | ordinal | NA | NA |
| Sharma, 2021  (India) | To identify socially vulnerable wards (administrative units) using a Social Vulnerability Index (SVI), developed based on 16 indicators using Principal Component Analysis. | Health/ Medicine | census or geographical data | 16 | Yes | numeric | numeric | NA | NA |
| Shega, 2012  (Canada) | To delineate the relationship between noncancer pain and cognitive impairment with social vulnerability. | Health/ Medicine | census or geographical data | 39 | No | numeric | numeric | Cognitive impairment, pain | Positive |
| Siagian, 2014  (Indonesia) | To quantify the social vulnerability of Indonesian districts to natural hazards, determining its driving factors and mapping its variations. | Climate/ Environment/ Disaster | mixed | 10 | Yes | numeric | ordinal | NA | NA |
| Snyder, 2020  (USA) | To develop a hierarchical socio-ecological vulnerability index that compares counties in the contiguous United States to capture a range of factors that might contribute to community vulnerability to Covid-19. | Mixed | census or geographical data | 18 | No | numeric | categorical | NA | NA |
| Solangaarachchi, 2012  (Australia) | To analyze the relative levels of social vulnerability of communities at the urban–bush interface in the Blue Mountains and Ku-ring-gai local council areas in New South Wales. | Climate/ Environment/ Disaster | census or geographical data | 29 | Yes | numeric | numeric | NA | NA |
| Stanturf, 2015  (Liberia) | To characterize vulnerability at the smallest scale practicable using the best available data while providing a national-scale assessment to highlight vulnerability “hot spots” in Liberia. | Health/ Medicine | census or geographical data | 18 | No | numeric | numeric | NA | NA |
| Su, 2015  (China) | To develop a composite index to measure social vulnerability of coastal cities. | Climate/ Environment/ Disaster | census or geographical data | 17 | No | numeric | numeric | NA | NA |
| Tanir, 2021  (USA) | To investigate spatiotemporal Socioeconomic flood vulnerability of the agricultural communities in the Potomac River Watershed (PRW) | Climate/ Environment/ Disaster | census or geographical data | 13 | Yes | numeric | categorical | NA | NA |
| Tascon-Gonzalez, 2020  (Spain) | To propose a methodology for the analysis of social vulnerability to floods based on the integration and weighting of a range of exposure and resistance (coping capacity) indicators, and to demonstrate the feasibility of the method using a study case. | Climate/ Environment/ Disaster | mixed | 24 | Yes | numeric | numeric | NA | NA |
| Tasnuva, 2021  (Bangladesh) | To construct a household-level social vulnerability at the microscale in the nine wards of Chalna Municipality (CM), Dacope upazila, in southwest coastal Bangladesh by employing the social vulnerability index. | Climate/ Environment/ Disaster | mixed | 33 | No | numeric | numeric | NA | NA |
| Tate, 2016  (USA) | To investigate post-flood property acquisition from the perspectives of cost effectiveness and social equity. | Climate/ Environment/ Disaster | census or geographical data | 12 | Yes | numeric | numeric | NA | NA |
| Toké, 2014  (USA) | To examine the social condition within regions of significant seismic hazard. | Climate/ Environment/ Disaster | census or geographical data | 20 | No | numeric | numeric | NA | NA |
| Török, 2017  (Romania) | To contribute to the ongoing research on vulnerability by quantifying the social vulnerability of Romanian settlements in the face of natural disasters. To facilitate decision making process and planning efforts to increase resilience of local communities. | Climate/ Environment/ Disaster | census or geographical data | 38 | No | numeric | numeric | NA | NA |
| Török, 2018  (Romania) | To investigates local-scale social vulnerability to flood hazards in Romania, aiming to identify the most vulnerable social and demographic groups across a wide range of geographical locations. | Climate/ Environment/ Disaster | census or geographical data | 28 | Yes | numeric | categorical | NA | NA |
| Török, 2021  (Romania) | To improve the existing methodology by quantifying the effects of climate change on social vulnerability by developing a set of vulnerability indicators. | Climate/ Environment/ Disaster | census or geographical data | 35 | Yes | numeric | numeric | NA | NA |
| Tragaki, 2018  (Greece) | To assess the physical and social vulnerability of the Peloponnese (Greece) to coastal hazards. | Climate/ Environment/ Disaster | census or geographical data | 6 | Yes | numeric | numeric | NA | NA |
| Varughese, 2021  (Canada) | To create a solid organ transplant frailty index (FI) and a social vulnerability index from assessment data and to evaluate associations between the FI and assessment, waitlist, and posttransplant outcomes. | Health/ Medicine | clinical data | 10 | No | numeric | numeric | Composite endpoint: death/delisting on transplant waitlist and death posttransplant | Mixed |
| Vincent, 2004  (Zambia) | To create an index to empirically assess relative levels of social vulnerability to climate change-induced variations in water availability and allow cross-country comparison in Africa. | Climate/ Environment/ Disaster | mixed | 9 | No | numeric | numeric | NA | NA |
| Wallace, 2015  (Multiple countries in Europe) | To investigate the SVI in relation to mortality and disability, independent of frailty, in middle-aged and older European adults, and examine how this relationship differs across countries. | Health/ Medicine | representative survey | 32 | No | numeric | numeric | mortality, disability | Positive |
| Godin, 2019  (Multiple countries in Europe) | To understand the association between social vulnerability and the odds of long-term care placement. | Health/ Medicine | - | - | - | - | - | Long term care placement | Mixed |
| Waly, 2021  (Egypt) | To analyse the social vulnerability of Alexandria city. | Climate/ Environment/ Disaster | mixed | 14 | Yes | numeric | ordinal | NA | NA |
| Ware, 2021  (South Africa) | To determine if parity is a predictor of social vulnerability in a large random sample of young women from an urban African township and (2) assess the relationships between parity, health, social vulnerability and household food insecurity. | Health/ Medicine | representative survey | 8 | No | numeric | ordinal | Household food insecurity | Positive |
| Yang, 2015  (China) | To quantify regional social vulnerability to natural hazards and map its temporal–spatial distribution in China. | Climate/ Environment/ Disaster | census or geographical data | 31 | Yes | numeric | ordinal | NA | NA |
| Yuan, 2020  (Multiple countries in Africa) | To identify which factor of social vulnerability predominantly affects infant mortality. | Health/ Medicine | census or geographical data | 6 | unclear | numeric | ordinal | Infant mortality rate | Positive |
| Zarghami, 2021  (Australia) | To develop a hybrid model to aggregate vulnerability indicators and to construct a social vulnerability index which combines F’ANP and applies it to a real world case study in a developing country. | Climate/ Environment/ Disaster | census or geographical data | 5 | Yes | numeric | numeric | NA | NA |
| Zebardast, 2013  (Iran) | To assess social vulnerability (SV) to earthquake hazards, this paper presents the development of a hybrid factor analysis and analytic network process model for aggregating vulnerability indicators into a composite index of SV to earthquake hazards. | Climate/ Environment/ Disaster | administrative data | 27 | Yes | numeric | numeric | NA | NA |
| Zhang, 2013 (China) | To analyze the social vulnerability to hazards and the sensitivity of each influencing factors, to achieve risk prevention and mitigation and to elaborate the plan of effective risk response strategies in Beijing. | Climate/ Environment/ Disaster | census or geographical data | 26 | Yes | numeric | numeric | NA | NA |
| Zhang, 2017  (China) | To assess social vulnerability to earthquake disaster. | Climate/ Environment/ Disaster | census or geographical data | 27 | No | numeric | numeric | NA | NA |
| Zhang, 2014  (China) | To create an index system of social vulnerability to floods constructed from three dimensions: population, economy, and flood prevention. | Climate/ Environment/ Disaster | census or geographical data | 36 | No | numeric | numeric | NA | NA |
| Zhou, 2014  (China) | To investigate the county-level spatial and temporal patterns in social vulnerability in China from 1980 to 2010. | Climate/ Environment/ Disaster | census or geographical data | 18 | Yes | numeric | numeric | NA | NA |
| Zhu, 2014  (China) | To assess the spatial distribution of health vulnerability to heat waves . | Climate/ Environment/ Disaster | census or geographical data | 13 | Yes | numeric | numeric | NA | NA |
