# Supplementary material for: Social vulnerability indices: a scoping review
Source: BMC Public Health. 2023 Jun 28;23:1253. doi: 10.1186/s12889-023-16097-6 (PMC10304642; doi:10.1186/s12889-023-16097-6)

## Additional File 4a. Frequency and proportion of replications, in descending order

| **Reference (Original SVI)** | **All Replications** | | **Environment or Disaster** | | **Health or Medicine** | | **Other** | |
| --- | --- | --- | --- | --- | --- | --- | --- | --- |
| Total number of studies | 174 | | 36 | | 130 | | 8 | |
|  | **n** | **%** | **n** | **%** | **n** | **%** | **n** | **%** |
| Flanagan BE, Gregory EW, Hallisey EJ, Heitgerd JL, Lewis B. A Social Vulnerability Index for Disaster Management. Journal of Homeland Security and Emergency Management. 2011 Jan 5 | 90 | 51.7 | 7 | 19.4 | 78 | 60.0 | 5 | 62.5 |
| Cutter SL, Boruff BJ, Shirley WL. Social Vulnerability to Environmental Hazards. Social Science Quarterly. 2003 Jun;84(2):242–61. | 24 | 13.8 | 20 | 55.6 | 3 | 2.3 | 1 | 12.5 |
| Nahas, M.I.; Ribeiro, C.; Esteves, O.; Moscovitch, S.; Martins, V.L. The map of social exclusion in Belo Horizonte: Methodology of building an urban management tool. Cad. Cienc. Soc. 2000, 7, 75–88. | 20 | 11.5 | 0 | 0.0 | 20 | 15.4 | 0 | 0.0 |
| Brazilian Social Vulnerability Atlas (<http://ivs.ipea.gov.br/index.php/pt/>)  IPEA (2015). Atlas da Vulnerabilidade Social nos Municípios Brasileiros. Brasília: Editorial IPEA | 15 | 8.6 | 1 | 2.8 | 14 | 10.8 | 0 | 0.0 |
| São Paulo Índice Paulista de Vulnerabilidade Social. Fundação Seade. Distribuição da população, segundo grupos do IPVS.  São Paulo: Fundação Seade; 2010. | 13 | 7.5 | 1 | 2.8 | 10 | 7.7 | 2 | 25.0 |
| Mavhura, E., Manyena, B., & Collins, A. E. (2017). An approach for measuring social vulnerability in context: The case of flood hazards in Muzarabani district, Zimbabwe. Geoforum, 86, 103-117. | 2 | 1.1 | 2 | 5.6 | 0 | 0.0 | 0 | 0.0 |
| Andrew MK, Mitnitski A, Rockwood K. Social Vulnerability, Frailty and Mortality in Elderly People. PLoS ONE. 2008;3(5). | 2 | 1.1 | 0 | 0.0 | 2 | 1.5 | 0 | 0.0 |
| Armaș, I., & Gavriș, A. (2013). Social vulnerability assessment using spatial multi-criteria analysis (SEVI model) and the Social Vulnerability Index (SoVI model)–a case study for Bucharest, Romania. Natural hazards and earth system sciences, 13(6), 1481-1499. | 1 | 0.6 | 1 | 2.8 | 0 | 0.0 | 0 | 0.0 |
| Armstrong JJ, Andrew MK, Mitnitski A, Launer LJ, White LR, Rockwood K. Social vulnerability and survival across levels of frailty in the Honolulu-Asia Aging Study. Age and Ageing. 2015 Jul;44(4):709–12. | 1 | 0.6 | 0 | 0.0 | 1 | 0.8 | 0 | 0.0 |
| Chen W, Cutter SL, Emrich CT, Shi P. Measuring social vulnerability to natural hazards in the Yangtze River Delta region, China. Int J Disaster Risk Sci. 2013 Dec;4(4):169–81. | 1 | 0.6 | 1 | 2.8 | 0 | 0.0 | 0 | 0.0 |
| de Loyola Hummell BM, Cutter SL, Emrich CT. Social Vulnerability to Natural Hazards in Brazil. Int J Disaster Risk Sci. 2016 Jun;7(2):111–22. | 1 | 0.6 | 1 | 2.8 | 0 | 0.0 | 0 | 0.0 |
| Ge, Y., Dou, W., & Dai, J. (2017). A new approach to identify social vulnerability to climate change in the Yangtze River delta. Sustainability, 9(12), 2236. | 1 | 0.6 | 1 | 2.8 | 0 | 0.0 | 0 | 0.0 |
| Hazards and Vulnerability Research Institute at the University of South Carolina. SoVI®: Social Vulnerability Index for the United States 2010–14 [Internet]. 2016 [cited 2022 Jan 27]. Available from: https://www.sc.edu/study/colleges_schools/artsandsciences/centers_and_institutes/hvri/data_and_resources/sovi/ | 1 | 0.6 | 1 | 2.8 | 0 | 0.0 | 0 | 0.0 |
| de Medeiros, M. D., & de Almeida, L. Q. (2016). Vulnerabilidade socioambiental no município de Natal, RN, BR. REDE-Revista Eletrônica do PRODEMA, 9(2). | 1 | 0.6 | 0 | 0.0 | 1 | 0.8 | 0 | 0.0 |
| Wallace, L.M.K., Theou, O., Pena, F. et al. Social vulnerability as a predictor of mortality and disability: cross-country differences in the survey of health, aging, and retirement in Europe (SHARE). Aging Clin Exp Res 27, 365–372 (2015). | 1 | 0.6 | 0 | 0.0 | 1 | 0.8 | 0 | 0.0 |

## Additional File 4b. Geographic Distribution of SVIs


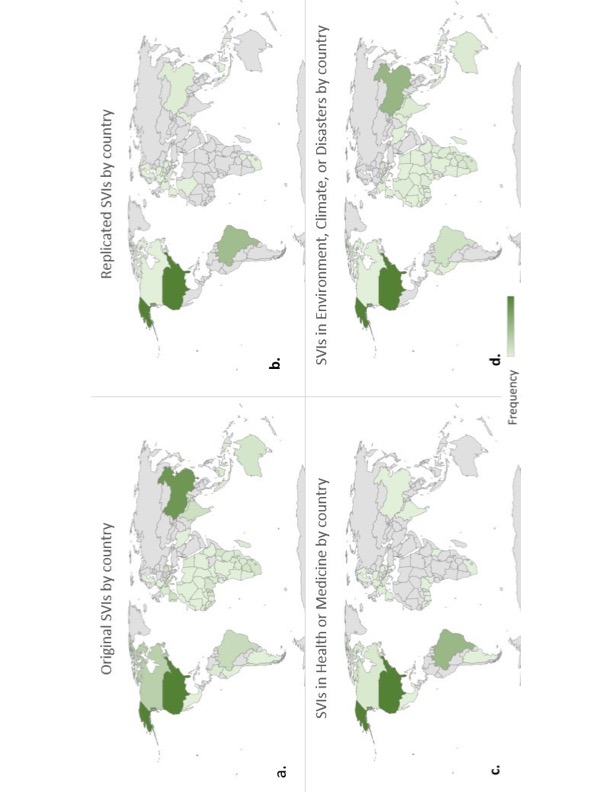

Supplement: Supplementary file 4 — Additional file 4. a. Frequency and proportion of replications, in descending order. b. Geographic Distribution of SVIs. [file 12889_2023_16097_MOESM4_ESM.docx]
