# Supplementary material for: Social vulnerability indices: a scoping review
Source: BMC Public Health. 2023 Jun 28;23:1253. doi: 10.1186/s12889-023-16097-6 (PMC10304642; doi:10.1186/s12889-023-16097-6)
Supplement: Supplementary file 5 — Additional file 5. Items (proportion of the domain) and domains (proportion of all SVIs). [file 12889_2023_16097_MOESM5_ESM.docx]

## Additional File 5. Items (proportion of the domain) and domains (proportion of all SVIs)

| Domain | Item | All  (n = 121) | | Environment, Climate or Disaster  (n = 92) | | Health or Medicine  (n = 27) | | Other  (n = 2) | |
| --- | --- | --- | --- | --- | --- | --- | --- | --- | --- |
|  |  | n | % | n | % | n | % | n | % |
|  | |  |  |  |  |  |  |  |  |
| **At risk populations** | | **92** | **76.0** | **80** | **87.0** | **11** | **40.7** | **1** | **50.0** |
|  | Seniors or Elderly | 78 | 84.8 | 73 | 91.3 | 4 | 36.4 | 1 | 100.0 |
|  | Children | 76 | 82.6 | 71 | 88.8 | 4 | 36.4 | 1 | 100.0 |
|  | Dependents | 50 | 54.3 | 44 | 55.0 | 6 | 54.5 | 0 | 0.0 |
|  | Institutionalized | 14 | 15.2 | 12 | 15.0 | 2 | 18.2 | 0 | 0.0 |
|  | Child Laborers | 3 | 3.3 | 2 | 2.5 | 0 | 0.0 | 1 | 100.0 |
|  | Teen Pregnancy | 2 | 2.2 | 0 | 0.0 | 1 | 9.1 | 1 | 100.0 |
|  | Victims of Domestic Violence | 1 | 1.1 | 0 | 0.0 | 1 | 9.1 | 0 | 0.0 |
|  |  |  |  |  |  |  |  |  |  |
| **Education** | | **90** | **74.4** | **68** | **73.9** | **20** | **74.1** | **2** | **100.0** |
|  |  |  |  |  |  |  |  |  |  |
| **Micro Level Socioeconomic Status** | | **80** | **66.1** | **58** | **63.0** | **20** | **74.1** | **2** | **100.0** |
|  | Income or Wealth | 75 | 93.8 | 54 | 93.1 | 19 | 70.4 | 2 | 100.0 |
|  | Income Assistance | 19 | 23.8 | 16 | 27.6 | 3 | 11.1 | 0 | 0.0 |
|  | Land Size | 11 | 13.8 | 11 | 19.0 | 0 | 0.0 | 0 | 0.0 |
|  | Savings or Debt | 5 | 6.3 | 5 | 8.6 | 0 | 0.0 | 0 | 0.0 |
|  | Food Insecurity | 3 | 3.8 | 2 | 3.4 | 1 | 3.7 | 0 | 0.0 |
|  | Access to Banking | 1 | 1.3 | 0 | 0.0 | 1 | 3.7 | 0 | 0.0 |
|  |  |  |  |  |  |  |  |  |  |
| **Household Composition** | | **75** | **62.0** | **54** | **58.7** | **20** | **74.1** | **1** | **50.0** |
|  | Size of Household | 50 | 66.7 | 46 | 85.2 | 4 | 20.0 | 0 | 0.0 |
|  | Single Parent or Female-Headed Household | 35 | 46.7 | 31 | 57.4 | 3 | 15.0 | 1 | 100.0 |
|  | Lives Alone | 17 | 22.7 | 3 | 5.6 | 14 | 70.0 | 0 | 0.0 |
|  | Child-Headed Household | 2 | 2.7 | 2 | 3.7 | 0 | 0.0 | 0 | 0.0 |
|  |  |  |  |  |  |  |  |  |  |
| **Employment** | | **74** | **61.2** | **63** | **68.5** | **9** | **33.3** | **2** | **100.0** |
|  | Unemployment | 62 | 83.8 | 53 | 84.1 | 7 | 77.8 | 2 | 100.0 |
|  | Occupation | 39 | 52.0 | 36 | 57.1 | 3 | 33.3 | 0 | 0.0 |
|  |  |  |  |  |  |  |  |  |  |
| **Housing** | | **68** | **56.2** | **56** | **60.9** | **12** | **44.4** | **0** | **0.0** |
|  | Housing Materials or Condition | 31 | 45.6 | 27 | 48.2 | 4 | 33.3 | 0 | 0.0 |
|  | House Ownership | 29 | 42.6 | 25 | 44.6 | 4 | 33.3 | 0 | 0.0 |
|  | House Without Necessities | 22 | 32.4 | 20 | 35.7 | 2 | 16.7 | 0 | 0.0 |
|  | Housing Type | 22 | 32.4 | 17 | 30.4 | 5 | 41.7 | 0 | 0.0 |
|  | Housing Price | 9 | 13.2 | 9 | 16.1 | 0 | 0.0 | 0 | 0.0 |
|  | Housing Vacancy | 4 | 5.9 | 4 | 7.1 | 0 | 0.0 | 0 | 0.0 |
|  | Group Housing | 3 | 4.4 | 1 | 1.8 | 2 | 16.7 | 0 | 0.0 |
|  | Homelessness | 1 | 1.5 | 0 | 0.0 | 1 | 8.3 | 0 | 0.0 |
|  |  |  |  |  |  |  |  |  |  |
| **Population Health Statistics** | | **67** | **55.4** | **56** | **60.9** | **9** | **33.3** | **2** | **100.0** |
|  | Migration | 30 | 44.8 | 27 | 48.2 | 3 | 33.3 | 0 | 0.0 |
|  | Average Age | 17 | 25.4 | 14 | 25.0 | 2 | 22.2 | 1 | 50.0 |
|  | Population Growth | 16 | 23.9 | 15 | 26.8 | 1 | 11.1 | 0 | 0.0 |
|  | Total Population | 12 | 17.9 | 12 | 21.4 | 0 | 0.0 | 0 | 0.0 |
|  | Birth Rate | 6 | 9.0 | 5 | 8.9 | 1 | 11.1 | 0 | 0.0 |
|  | Mortality Rate | 6 | 9.0 | 2 | 3.6 | 3 | 33.3 | 1 | 50.0 |
|  | Life Expectancy | 2 | 3.0 | 2 | 3.6 | 0 | 0.0 | 0 | 0.0 |
|  |  |  |  |  |  |  |  |  |  |
| **Gender or Sex** | | **60** | **49.6** | **56** | **60.9** | **3** | **11.1** | **1** | **50.0** |
|  |  |  |  |  |  |  |  |  |  |
| **Density** | | **57** | **47.1** | **53** | **57.6** | **3** | **11.1** | **1** | **50.0** |
|  | Population Density | 47 | 82.5 | 44 | 83.0 | 2 | 66.7 | 1 | 100.0 |
|  | Urban or Rural | 20 | 35.1 | 19 | 35.8 | 1 | 33.3 | 0 | 0.0 |
|  | Building Density | 14 | 24.6 | 13 | 24.5 | 0 | 0.0 | 1 | 100.0 |
|  |  |  |  |  |  |  |  |  |  |
| **Micro Level Socioeconomic Status** | | **51** | **42.1** | **43** | **46.7** | **7** | **25.9** | **1** | **50.0** |
|  | Community Poverty or Standard of Living | 45 | 88.2 | 37 | 86.0 | 7 | 100.0 | 1 | 100.0 |
|  | Gross Domestic Product or Community Finances | 6 | 11.8 | 6 | 14.0 | 0 | 9.0 | 0 | 0.0 |
|  | Trade | 2 | 3.9 | 2 | 4.7 | 0 | 9.0 | 0 | 0.0 |
|  |  |  |  |  |  |  |  |  |  |
| **Healthcare Infrastructure** | | **49** | **40.5** | **40** | **43.5** | **8** | **29.6** | **1** | **50.0** |
|  | Healthcare Facilities | 32 | 65.3 | 26 | 65.0 | 5 | 62.5 | 1 | 100.0 |
|  | Medical Staff | 21 | 42.9 | 20 | 50.0 | 1 | 12.5 | 0 | 0.0 |
|  | Health Insurance | 14 | 28.6 | 10 | 25.0 | 3 | 37.5 | 1 | 100.0 |
|  | Public Health | 6 | 12.2 | 5 | 12.5 | 1 | 12.5 | 0 | 0.0 |
|  | Basic Services | 4 | 8.2 | 2 | 5.0 | 2 | 25.0 | 0 | 0.0 |
|  | Health Expenditure | 2 | 4.1 | 2 | 5.0 | 0 | 0.0 | 0 | 0.0 |
|  | Avoidable Hospital Admissions | 1 | 2.0 | 1 | 2.5 | 0 | 0.0 | 0 | 0.0 |
|  |  |  |  |  |  |  |  |  |  |
| **Transport** | | **40** | **33.1** | **31** | **33.7** | **9** | **33.3** | **0** | **0.0** |
|  | Transport Infrastructure | 23 | 57.5 | 19 | 61.3 | 4 | 44.4 | 0 | 0.0 |
|  | Road Infrastructure | 12 | 30.0 | 11 | 35.5 | 1 | 11.1 | 0 | 0.0 |
|  | Access to Railways, Roads or Transit (community) | 8 | 20.0 | 7 | 22.6 | 1 | 11.1 | 0 | 0.0 |
|  | Able to Get Places (Individual) | 4 | 10.0 | 0 | 0.0 | 4 | 44.4 | 0 | 0.0 |
|  |  |  |  |  |  |  |  |  |  |
| **Ethnicity or Race** | | **39** | **32.2** | **36** | **39.1** | **2** | **7.4** | **1** | **50.0** |
|  |  |  |  |  |  |  |  |  |  |
| **Water and Waste** | | **32** | **26.4** | **29** | **31.5** | **2** | **7.4** | **1** | **50.0** |
|  | Water Infrastructure & Safety | 26 | 81.3 | 24 | 82.8 | 2 | 100.0 | 0 | 0.0 |
|  | Waste Infrastructure and Collection | 24 | 75.0 | 21 | 72.4 | 2 | 100.0 | 1 | 100.0 |
|  |  |  |  |  |  |  |  |  |  |
| **Social connection and capital** | | **26** | **21.5** | **10** | **10.9** | **16** | **59.3** | **0** | **0.0** |
|  | Relationships with Family | 15 | 57.7 | 1 | 10.0 | 14 | 87.5 | 0 | 0.0 |
|  | Relationships with Friends | 15 | 57.7 | 2 | 20.0 | 13 | 81.3 | 0 | 0.0 |
|  | General Relationships | 12 | 46.2 | 4 | 40.0 | 8 | 50.0 | 0 | 0.0 |
|  | Emotional Support Available | 10 | 38.5 | 0 | 0.0 | 10 | 62.5 | 0 | 0.0 |
|  | General Support Available to Help | 7 | 26.9 | 1 | 10.0 | 6 | 37.5 | 0 | 0.0 |
|  | Relationships with Neighbours | 7 | 26.9 | 1 | 10.0 | 6 | 37.5 | 0 | 0.0 |
|  | Telephone Use | 6 | 23.1 | 0 | 0.0 | 6 | 37.5 | 0 | 0.0 |
|  | Ability to Give | 5 | 19.2 | 3 | 30.0 | 2 | 12.5 | 0 | 0.0 |
|  | Specific Task Support Available | 5 | 19.2 | 0 | 0.0 | 5 | 31.3 | 0 | 0.0 |
|  | Help Availiable in a Crisis | 4 | 15.4 | 0 | 0.0 | 4 | 25.0 | 0 | 0.0 |
|  | Relationships with Children | 4 | 15.4 | 1 | 10.0 | 3 | 18.8 | 0 | 0.0 |
|  | Community Social Support | 3 | 11.5 | 2 | 20.0 | 1 | 6.3 | 0 | 0.0 |
|  | Loving Support Availiable | 3 | 11.5 | 0 | 0.0 | 3 | 18.8 | 0 | 0.0 |
|  | Relationships with Community | 3 | 11.5 | 2 | 20.0 | 1 | 6.3 | 0 | 0.0 |
|  | Relationships with Spouse | 3 | 11.5 | 0 | 0.0 | 3 | 18.8 | 0 | 0.0 |
|  |  |  |  |  |  |  |  |  |  |
| **Individual Communication** | | **25** | **20.7** | **13** | **14.1** | **12** | **44.4** | **0** | **0.0** |
|  | Ability to Communicate (Oral or Written) | 24 | 96.0 | 13 | 100.0 | 11 | 91.7 | 0 | 0.0 |
|  | Sensory Problems | 1 | 4.0 | 0 | 0.0 | 1 | 8.3 | 0 | 0.0 |
|  |  |  |  |  |  |  |  |  |  |
| **Disaster Preparedness** | | **23** | **19.0** | **20** | **21.7** | **3** | **11.1** | **0** | **0.0** |
|  | Access to Internet, Phone or Radio | 20 | 87.0 | 17 | 85.0 | 3 | 100.0 | 0 | 0.0 |
|  | Community Disaster Resources | 7 | 30.4 | 7 | 35.0 | 0 | 0.0 | 0 | 0.0 |
|  | First Responders | 3 | 13.0 | 3 | 15.0 | 0 | 0.0 | 0 | 0.0 |
|  |  |  |  |  |  |  |  |  |  |
| **Marital Status** | | **22** | **18.2** | **10** | **10.9** | **12** | **44.4** | **0** | **0.0** |
|  |  |  |  |  |  |  |  |  |  |
| **Land Use** | | **21** | **17.4** | **21** | **22.8** | **0** | **0.0** | **0** | **0.0** |
|  | General Land Use | 12 | 57.1 | 12 | 57.1 | 0 | 0.0 | 0 | 0.0 |
|  | Farming or Soil Use | 11 | 52.4 | 11 | 52.4 | 0 | 0.0 | 0 | 0.0 |
|  | Forest | 5 | 23.8 | 5 | 23.8 | 0 | 0.0 | 0 | 0.0 |
|  | Green Space | 3 | 14.3 | 3 | 14.3 | 0 | 0.0 | 0 | 0.0 |
|  | Ecological Land Use | 1 | 4.8 | 1 | 4.8 | 0 | 0.0 | 0 | 0.0 |
|  |  |  |  |  |  |  |  |  |  |
| **Social Engagement** | | **19** | **15.7** | **5** | **5.4** | **14** | **51.9** | **0** | **0.0** |
|  | Clubs or Community Centers | 10 | 52.6 | 1 | 20.0 | 9 | 64.3 | 0 | 0.0 |
|  | Golf, Physical Leisure or Walking | 10 | 52.6 | 0 | 0.0 | 10 | 71.4 | 0 | 0.0 |
|  | Church or Religion | 9 | 47.4 | 0 | 0.0 | 9 | 64.3 | 0 | 0.0 |
|  | Amount of Social Engagement | 9 | 47.4 | 2 | 40.0 | 7 | 50.0 | 0 | 0.0 |
|  | Volunteering | 8 | 42.1 | 2 | 40.0 | 6 | 42.9 | 0 | 0.0 |
|  | Feelings Towards Social Engagement | 6 | 31.6 | 2 | 40.0 | 4 | 28.6 | 0 | 0.0 |
|  | Activities Around the Home (i.e. Garden) | 5 | 26.3 | 0 | 0.0 | 5 | 35.7 | 0 | 0.0 |
|  | Cards or Games | 5 | 26.3 | 0 | 0.0 | 5 | 35.7 | 0 | 0.0 |
|  | Hobby, Project or Further Education | 3 | 15.8 | 0 | 0.0 | 3 | 21.4 | 0 | 0.0 |
|  | Pets | 1 | 5.3 | 0 | 0.0 | 1 | 7.1 | 0 | 0.0 |
|  |  |  |  |  |  |  |  |  |  |
| **Power Sources** | | **19** | **15.7** | **18** | **19.6** | **1** | **3.7** | **0** | **0.0** |
|  | Power and Electricity Infrastructure | 15 | 78.9 | 14 | 77.8 | 1 | 100.0 | 0 | 0.0 |
|  | Biomass | 5 | 26.3 | 5 | 100.0 | 0 | 0.0 | 0 | 0.0 |
|  |  |  |  |  |  |  |  |  |  |
|  |  |  |  |  |  |  |  |  |  |
| **Personal Attitudes and Expectations** | | **13** | **10.7** | **1** | **1.1** | **12** | **44.4** | **0** | **0.0** |
|  | Control | 10 | 76.9 | 0 | 0.0 | 10 | 83.3 | 0 | 0.0 |
|  | Expectations of Self and Others | 8 | 61.5 | 1 | 100.0 | 7 | 58.3 | 0 | 0.0 |
|  | Satisfaction with Life | 7 | 53.8 | 0 | 0.0 | 7 | 58.3 | 0 | 0.0 |
|  | Attitude Towards Life | 6 | 46.2 | 0 | 0.0 | 6 | 50.0 | 0 | 0.0 |
|  | Self Worth or Self Esteem | 3 | 23.1 | 0 | 0.0 | 3 | 25.0 | 0 | 0.0 |
|  | Major Life Events | 1 | 7.7 | 0 | 0.0 | 1 | 8.3 | 0 | 0.0 |
|  | Hope for the Future | 0 | 0.0 | 0 | 0.0 | 0 | 0.0 | 0 | 0.0 |
|  |  |  |  |  |  |  |  |  |  |
| **Industry** | | **13** | **10.7** | **13** | **14.1** | **0** | **0.0** | **0** | **0.0** |
|  | Tourism or Hospitality | 8 | 61.5 | 8 | 61.5 | 0 | 0.0 | 0 | 0.0 |
|  | Specific Industries (e.g. Cotton) | 3 | 23.1 | 3 | 23.1 | 0 | 0.0 | 0 | 0.0 |
|  | General Industries (e.g. Primary) | 3 | 23.1 | 3 | 23.1 | 0 | 0.0 | 0 | 0.0 |
|  |  |  |  |  |  |  |  |  |  |
| **Environment and Climate Events** | | **13** | **10.7** | **12** | **13.0** | **0** | **0.0** | **1** | **50.0** |
|  | Flood | 7 | 53.8 | 7 | 58.3 | 0 | 0.0 | 0 | 0.0 |
|  | Extreme Weather | 7 | 53.8 | 6 | 50.0 | 0 | 0.0 | 1 | 100.0 |
|  | Rainfall or Drought | 5 | 38.5 | 5 | 41.7 | 0 | 0.0 | 0 | 0.0 |
|  | Landslides | 2 | 15.4 | 2 | 16.7 | 0 | 0.0 | 0 | 0.0 |
|  |  |  |  |  |  |  |  |  |  |
| **Government Aptitude and Investments** | | **9** | **7.4** | **9** | **7.4** | **0** | **0.0** | **0** | **0.0** |
|  | School Infrastructure | 6 | 66.7 | 6 | 66.7 | 0 | 0.0 | 0 | 0.0 |
|  | Capacity for Governance | 2 | 22.2 | 2 | 22.2 | 0 | 0.0 | 0 | 0.0 |
|  | Corruption | 2 | 22.2 | 2 | 22.2 | 0 | 0.0 | 0 | 0.0 |
|  | Research and Development Infrastructure | 1 | 11.1 | 1 | 11.1 | 0 | 0.0 | 0 | 0.0 |
|  |  |  |  |  |  |  |  |  |  |
| **Isolation or Loneliness** | | **8** | **6.6** | **0** | **0.0** | **8** | **29.6** | **0** | **0.0** |
|  |  |  |  |  |  |  |  |  |  |
| **Health Conditions** | | **8** | **6.6** | **5** | **5.4** | **2** | **7.4** | **1** | **50.0** |
|  | Chronic Health Conditions or their Risk Factors | 4 | 50.0 | 2 | 40.0 | 1 | 50.0 | 1 | 100.0 |
|  | HIV / AIDS | 3 | 37.5 | 3 | 60.0 | 0 | 0.0 | 0 | 0.0 |
|  | Poor Mental Health | 2 | 25.0 | 1 | 20.0 | 1 | 50.0 | 0 | 0.0 |
|  | Specific Disease Incidence | 1 | 12.5 | 1 | 20.0 | 0 | 0.0 | 0 | 0.0 |
|  | Specific Disease after Flood | 1 | 12.5 | 1 | 20.0 | 0 | 0.0 | 0 | 0.0 |
|  | Adherence to Medical Advice | 1 | 12.5 | 0 | 0.0 | 1 | 50.0 | 0 | 0.0 |
|  |  |  |  |  |  |  |  |  |  |
| **Political Stability** | | **6** | **5.0** | **3** | **3.3** | **3** | **11.1** | **0** | **0.0** |
|  | Refugees Displaced | 5 | 83.3 | 3 | 100.0 | 2 | 66.7 | 0 | 0.0 |
|  | Political Armed Conflict | 1 | 16.7 | 0 | 0.0 | 1 | 33.3 | 0 | 0.0 |
|  |  |  |  |  |  |  |  |  |  |
| **Noise or Air Pollution** | | **3** | **2.5** | **0** | **0.0** | **2** | **7.4** | **1** | **50.0** |

* In this table, the unit is individual SVI (total studies remain 118)
